# Brain Gamma-Stimulation: Mechanisms and Optimization of Impact

**DOI:** 10.3390/biology14121722

**Published:** 2025-12-01

**Authors:** Konstantin V. Lushnikov, Dmitriy A. Serov, Maxim E. Astashev, Valeriy A. Kozlov, Alexander Melerzanov, Maria V. Vedunova

**Affiliations:** 1 Institute of Biology and Biomedicine, Lobachevsky State University of Nizhny Novgorod, 23 Gagarin Avenue, 603022 Nizhny Novgorod, Russia; kolushn1@yandex.ru (K.V.L.); mvedunova@yandex.ru (M.V.V.); 2 Prokhorov General Physics Institute of the Russian Academy of Sciences, Vavilov Str. 38, 119991 Moscow, Russia; dmitriy_serov_91@mail.ru (D.A.S.); astashev@yandex.ru (M.E.A.); 3 Federal Research Center “Pushchino Scientific Center for Biological Research of the Russian Academy of Sciences”, Institute of Cell Biophysics of the Russian Academy of Sciences, 3 Institutskaya St., 142290 Pushchino, Russia; 4 Department of Fundamental Sciences, Bauman Moscow State Technical University, 5 2nd Baumanskaya St., 105005 Moscow, Russia; 5 Institute of General Pathology and Pathophysiology, 8 Baltiyskaya St., 125315 Moscow, Russia; m83071@gmail.com

**Keywords:** gamma stimulation, visual stimulation, neural network, Alzheimer’s disease, dementia

## Abstract

The γ-rhythm plays a key role in coordinating the activity of the major brain systems and facilitating higher-level neurological processes. Several pathological conditions are associated with impaired generation or regulation of γ-oscillations. Modulating the γ-rhythm using periodic signals is considered a potential way to halt and/or treat such neurodegenerative processes. Despite the extensive knowledge gained in this field over the last 70 years, a unified theory linking the effectiveness of γ-stimulation to the characteristics of the stimulus and the stimulated remains elusive. In this review, we conducted a quantitative analysis of these relationships. The γ-stimulation effectiveness depends on species, age, frequency, and stimulus type. Here, we found with our analysis that experiments using white light were more effective than red and infrared. The range of effective central frequencies depends on age. We also showed that AD patients and mouse models respond differently to γ-stimulation, so the careful selection of study subjects is essential when assessing therapeutic potential. This review also provides an overview of the mechanisms of γ-stimulation and makes recommendations for optimizing the method based on these mechanisms. Our findings may be useful to understanding -stimulation mechanisms, planning future experiments for research groups and identifying potential therapeutic γ-stimulation regimens.

## Simple Summary

Changes in the brain’s electrical rhythm at a frequency of 30–60 Hz (γ-rhythm) play an important role in cognitive activity, learning, problem solving, and memory. Changes in γ-rhythm are associated with a wide range of nervous system diseases (Alzheimer’s disease, dementia, and others). The use of periodic stimuli (light, sound, and their combination) affects γ-rhythm and is considered a potential means of preventing and/or treating nervous system disorders. A large amount of data has now been accumulated on this issue, and the existing knowledge needs to be systematized. This paper analyses the effectiveness of periodic stimuli on the γ-rhythm depending on the frequency of stimulation, its nature (light, sound), additional properties (color, method of exposure), species affiliation, and age of the ‘test subjects’. This article provides an overview of the ways in which periodic stimulation changes the γ-rhythm and suggests ways to increase the effectiveness of γ-rhythm stimulation in humans.

## 1. Introduction

Non-invasive physical methods are becoming increasingly prevalent in a variety of sectors, including industry, agriculture, and medicine. These modern approaches are being incorporated into our lives and are gradually replacing traditional chemical methods [1,2,3]. In this instance, the development of personalized, non-invasive medicine occupies a significant position. As our species name, *Homo sapiens*, suggests, the primary factor contributing to an individual’s quality of life is mental well-being and the capacity to maintain high cognitive function throughout life [4,5]. In cases of severe age-related changes and neurodegenerative diseases, this issue goes beyond mere comfort to encompass the fulfillment of fundamental needs and the preservation of life itself. Cognitive decline associated with aging is becoming an increasingly serious public health problem. It is estimated that, by the year 2050, 152.8 million people worldwide will be living with dementia [6]. Although there are numerous treatment options for cognitive impairment and dementia, a comprehensive cure remains elusive [7]. Consequently, there is an urgent need to develop additional treatments that can help to alleviate the personal, social and economic burdens associated with the increasing number of dementia diagnoses [8]. Rhythmic oscillations in the brain can be detected using local field potential, electrocorticography, electroencephalography, and magnetoencephalography. These oscillations are generated by the activity of different neuronal populations (neuronal ensembles) in various brain regions. These oscillations occur at the following frequencies: delta (1–4 Hz), theta (4–8 Hz), alpha (8–12 Hz), beta (15–30 Hz), gamma (30–90 Hz), and high gamma (>50 Hz) [9,10]. They control (or are a product of) the synchronization of neural impulses at the micro level, and at the macro level they ensure communication between different parts of the cortex, coordinating the temporal and spatial connectivity of the brain [11,12]. The γ-rhythm ensures the integrative activity of the brain during sensory, cognitive, and executive processes in humans and animals [13,14,15]. It is well established that the γ-rhythm plays a role in the processing of information during synchronization in the 35–85 Hz range, a process that occurs between distant regions of the brain [16,17].

**Figure 1:**
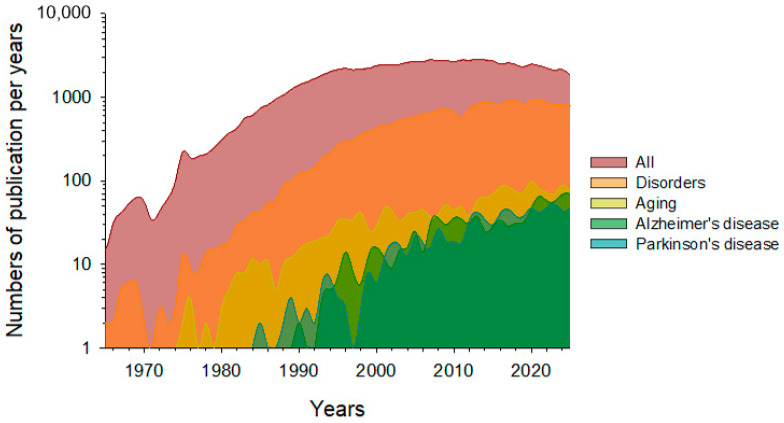
The dynamics of global publication activity on specific topics in gamma stimulation research. The search results for the following keywords are presented: ‘gamma stimulation’ (maroon line and fill), ‘gamma stimulation’ + ‘disorders’ (orange line and fill), ‘gamma stimulation’ + ‘aging’ (yellow line and fill), ‘gamma stimulation’ + ‘Alzheimer’s disease’ (green line and fill) and ‘gamma stimulation’ + ‘Parkinson’s disease’ (blue line and fill). The data were extracted from PubMed (https://pubmed.ncbi.nlm.nih.gov; accession date: 22 October 2025).

The use of periodic stimuli to trigger the γ-rhythm has been the subject of extensive research for a long time. The first studies of this phenomenon date back to the 1950s. The following diagram illustrates the current rate of global publications concerning γ-stimulation (Figure 1).

Overall publication activity saw active growth from 1980 to 2000. The number of published papers remained steady between 2010 and 2020, before declining gradually between 2020 and 2025. This observed trend may highlight several important points. Firstly, the empirical knowledge base on this topic may have reached saturation. Secondly, there is a lack of general quantitative patterns that could accurately predict and select the most promising areas. Despite the overall decline in publication activity, specific areas of γ-stimulation application continue to experience active growth. Notably, the number of publications focusing on the using of γ-stimulation to prevent age-related brain changes, or as a potential therapy for neurodegenerative diseases is increasing. Papers devoted to neurodegenerative diseases currently account for up to a third of the total pool of papers on γ-stimulation, indicating growth in applied research in this area.

There is sparse data in the global literature on the effectiveness of γ-stimulation. However, there are review articles devoted to systematizing the available data, identifying the relationship between effectiveness and stimulus characteristics, and analyzing side effects [18,19]. Furthermore, a significant body of work has been published on the molecular and cellular mechanisms of action, as well as on individual aspects that increase their effectiveness. However, no systematic studies have been conducted to describe clear, quantitative relationships between the effectiveness of γ-stimulation and characteristics such as the central frequency of the stimulus, the stimulus type, the wavelength for visual stimulation, the age of the subjects, and the species (when studying other animals).

This review aims to partially fill this gap. The aim of the study was to identify key parameters determining the effectiveness of γ-stimulation, and to assess the contribution of these parameters to the overall effectiveness of the intervention. Individual aspects of γ-stimulation’s effectiveness were also assessed. This review will also provide an overview of γ-stimulation methods, the molecular and cellular mechanisms of γ-stimulation, the relationship between the γ-rhythm and key brain systems, and its potential therapeutic effects. It will also propose avenues for optimizing γ-stimulation.

## 2. General Functions of γ-Rhythms, Mechanism of Regulations

**Figure 2:**
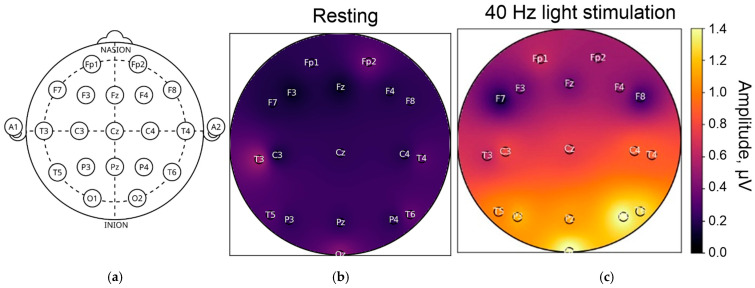
Brain response to 40 Hz stimulation during visual stimulation, according to our own experimental data: diagram of the electrode placement during EEG-recording (**a**), EEG in the frequency range of 39–41 Hz in resting state without stimulation (**b**) and brain response on 40 Hz visual stimulation (**c**). Resting and response amplitudes were recorded in equal conditions and shown in μV.

Gamma oscillations, characterized by a fast rhythm, allow excitation in a neural network to temporarily escape subsequent inhibition. This increases the efficiency, precision and selectivity of communication between different areas of the brain, a phenomenon known as interareal coherence [20]. Studies have shown that interneurons expressing parvalbumin (PV+) and somatostatin (SST+) play a key role in maintaining gamma activity in the cerebral cortex and hippocampus. The frequency of gamma oscillations is primarily determined by GABA receptor-induced inhibitory postsynaptic currents (Figure 2) [21,22,23].

It is currently suggested that, in different areas of the brain, there are fundamental mechanisms of the neural network responsible for storing and processing information, as well as supporting attention, cognition, and memory [12,19,24,25,26]. These rhythms have been recorded and studied in the cerebral cortex, the hippocampus, the amygdala, the olfactory bulb, the striatum, and the brainstem. Their role in controlling visual attention, object perception, sensory processing, short-term memory, retaining relevant information in working memory, encoding words, making cross-modal semantic comparisons, movement, and emotion has been demonstrated [12,19]. Figure 2 shows the results of our own experimental studies. It is clear that the parietal and occipital regions respond most actively to stimulation. In our own studies (as yet unpublished), we also tested the brain’s response to visual stimulation in the range of 30 to 50 Hz and found that different EEG leads recorded responses to different frequencies (32, 35, 42, 47 Hz, etc.). The response is highly individual. Therefore, the patterns of response of different brain regions to gamma stimulation at different frequencies remain to be fully described and elucidated.

By contrast, disruption to gamma oscillations leads to abnormal neural activity and brain dysfunction [4]. The γ-oscillations are altered in various brain diseases, including Alzheimer’s disease (AD), Parkinson’s disease, stroke, schizophrenia, and autism spectrum disorders [27,28,29,30,31,32]. Studies suggest that gamma oscillations may serve as potential biomarkers of neural imbalance or interneuron dysfunction, reflecting the underlying pathophysiological mechanisms of critical neural functions in neuropsychiatric diseases such as Alzheimer’s and Parkinson’s diseases [12,19,33,34,35]. Restoring normal gamma activity could be a therapy to improve higher-order cognitive functions, sensorimotor integration, working memory, attention, perceptual binding and network synchronization. Considerable evidence suggests that γ-synchronization affects neural circuit function and behavior [34,36,37,38]. γ-synchronization, whether performed by non-invasive or invasive methods, has been shown to have a powerful neuroprotective effect in brain diseases [39,40,41].

Neural synchronization is defined as the process by which the neural activity of a subject aligns with the frequency of repetitive sensory rhythms. This phenomenon can be observed as an increase in the electroencephalogram (EEG) spectrum power at the excitation frequency, otherwise referred to as the steady-state response [42]. At present, a variety of stimulation methods are employed for the purpose of inducing the γ-rhythm. These include sensory stimulation (auditory, visual, and tactile), optogenetics, transcranial electrical or magnetic stimulation, and deep brain electrical stimulation [19,35].

For over three decades, electrophysiologists have been aware of the relationship between the severity of Alzheimer’s disease (AD) and changes in the amplitude of electroencephalogram (EEG) oscillations at a frequency of 40 Hz [43]. The interest in intrinsic, induced, and 40 Hz-evoked oscillatory neural potentials and their relationship with cognitive functions arose even earlier [16,44,45,46,47,48,49,50,51,52]. In the following decades, and until recently, a significant number of publications described the results of studies that confirmed the importance of oscillations with a frequency of 40 Hz.

Furthermore, visual stimulation at 40 Hz has been demonstrated to enhance reaction time in a target detection task [53]. Subsequent research revealed a preference for frequencies of 10, 20, 40, and 80 Hz on the part of neural oscillators when human synchronous oscillations were measured in response to stimulation at 1–100 Hz [54]. In 2009, significant data were obtained on the importance of rapidly excitable PV+ interneurons for the emergence and maintenance of γ-oscillations, primarily in the 40 Hz range [53,54]. Later, the role of neuronal plasticity of CP-AMPARs- and mGluRs-dependent calcium signaling of PV+ interneurons and their interaction with SST+ interneurons in maintaining γ-oscillations was clarified [55,56,57].

The utilization of neurostimulation at 40 Hz as a therapeutic modality for the remediation of spectral aberrations in Alzheimer’s disease (AD) has emerged as a promising avenue for research. In 2016, researchers began to propose the use of γ-training using sensory stimulation (GENUS system, Gamma ENtrainment Using Sensory Stimuli) in the form of visual stimulation with stroboscopic flickering at 40 Hz for the treatment of AD [24,58]. The GENUS system utilized white light-emitting diodes (LEDs) with a correlated color temperature of 4000 Kelvin (K), exhibiting flicker at a frequency of 40 hertz (Hz) and a duty cycle of 50 per cent, with the objective of providing visual stimulation. The temporal modulation is of a stroboscopic nature, meaning that the flickering occurs with a modulation depth of 100%. In a series of experiments involving validated mouse models of AD, in which the light characteristics remained constant, GENUS therapy demonstrated a favorable neuroprotective effect [19,24,25,26,30,58,59,60,61]. Its utilization has been demonstrated to curtail amyloid production and enhance its elimination from the hippocampus and prefrontal cortex. Moreover, it has been shown to augment recognition and visual–spatial memory subsequent to “therapy” comprising daily 1 h sessions for one or more weeks [37,62].

Synchronized light and sound stimulation at 40 Hz has been shown to effectively induce corresponding brain activity at the same frequency [12,61]. Overall, 40 Hz brain stimulation is associated with reduced neuroinflammation, enhanced synaptic transmission, and increased expression of genes associated with synaptic plasticity. These effects improve cognitive function by activating intracellular signaling pathways [63,64,65,66,67,68,69]. GENUS at 40 Hz has been shown to increase cytokine expression in microglia, normalize circadian rhythms and decrease the amount of amyloid plaques [58,63,70]. Furthermore, other studies have demonstrated that multisensory stimulation at 40 Hz enhances the glymphatic clearance rate of Aβ via the glial clearance pathway [70].

Research is currently underway to identify the most effective treatment protocols and determine the mechanisms of gamma stimulation. It is important to carefully consider the treatment parameters.

This review will not provide detailed information on the types of γ-stimulation and their effects on various diseases. These are described in great detail in a recent comprehensive review [19]. Here, our aim is to focus on the general quantitative dependence of gamma stimulation effectiveness on stimulus and response system characteristics, mechanisms of action, and issues of gamma stimulation optimization.

## 3. Literature Data Analysis Protocol

### 3.1. Criteria for Including Publications in the Analysis

The work represents a quantitative synthesis or meta-analytical review based on previously published data. A literature search was performed using the publicly available databases PubMed (https://pubmed.ncbi.nlm.nih.gov/), Google Scholar (https://scholar.google.com/), and the websites of major publishers: Nature (https://www.nature.com/), MDPI (https://www.mdpi.com/), and others. The search was performed using the keywords ‘gamma stimulation’, ‘gamma frequency stimulation’, or ‘50 Hz brain rhythm stimulation’. To refine the search, we used combinations with additional keywords, such as: ‘gamma stimulation’ + ‘disorders’ (Alzheimer’s disease, Parkinson’s disease, etc.), ‘gamma stimulation’ + ‘aging’, ‘gamma stimulation’ + ‘sleeping’, ‘gamma stimulation’ + ‘sound’ or ‘light’, ‘gamma stimulation’ + ‘human’ or ‘animals’, etc. When including articles in the analysis, preference was given to articles published in the last 10 years (2015–2025) and 20 years (2005–2025). The shares of these works were 62% and 89%, respectively. Works published earlier (before 2005) were included if they contained pioneering data, important results for understanding the mechanisms of gamma rhythm functioning, or results that were not published in later periods. Publications that included completely identical experimental conditions to those already added, or those with insufficiently well-described experimental conditions (duration, frequency, nature of the stimulus, and its characteristics) were excluded from the analysis.

### 3.2. Data Processing

This review used a quantitative analysis that included papers which clearly specified the following stimulus characteristics: central frequency; stimulus source; exposure time; participant age; and status (healthy participants, patients, or animal models, with detailed inclusion and exclusion criteria). Since the effects of gamma stimulation vary in nature, direction and amplitude, comparing them with each other or constructing quantitative diagrams is significantly complicated. Additionally, it is impossible to plot gene expression (a.u.), dendrite branch length (μm) and field potential (mV) on a single graph. To overcome these limitations, we employed a well-established approach [71]. All quantitative changes described in the papers were expressed as percentages relative to the control, and then the absolute value of this was taken. Since some effects could be in the single percent range while others could be in the hundreds, the resulting values were log-transformed (see Appendix A).

As described previously, to enable comparison of disparate effects, we first converted to dimensionless quantities and calculated the effects of the treatments. We calculated the effects using Formula (1):

(1) $$Effect\;modulus={log}_{10}\left(\left|\left(\frac{A-\Delta A}{A}\right)\times 100\%\right|\right),$$

where *A* is the initial value of the measured characteristic (EEG potential, proportion of differentiated cells, mental performance scores, etc.), and *ΔA* is the value of the same parameter after γ-stimulation (in the same units as *A*). Expressing the values as percentages allows us to convert to dimensionless quantities and compare the effectiveness of γ-stimulation for different targets (see below, Section 4.5). Taking the absolute values allows us to adequately average the contributions of disparate effects. Further logarithmization allowed us to adequately assess the significance of statistical differences between groups, where effect sizes vary from units to hundreds of percent. The initial quantitative values of *A* and *ΔA* for the analysis were taken from the texts and/or graphs of the analyzed manuscripts.

The calculated data were processed using non-parametric statistics. We used a Kruskal–Wallis ANOVA with a post hoc Dunn’s test to evaluate the significance of statistical differences. If the differences in median values between treatment groups were greater than would be expected by chance (*p* < 0.05), we proceeded with a pairwise multiple comparison using Dunn’s test (all pairwise comparisons without control). *p*-values were provided for the results of the Dunn’s test. To assess the significance of the data in the case of constructing 3D maps, we calculated z-scores according to the Formula (2)

(2) $$z_{i}=\left(x_{i}-µ\right)/\sigma$$

where *z_i_* is z-score of individual *i* value, *x* is individual *i* value, μ is mean, σ is standard deviation. Statistically significant differences are those in which *z_i_* ≥ 2.

## 4. Dependence of the Effectiveness of γ-Stimulation on the Characteristics of the Stimulus and Responding Systems

### 4.1. Dependence of the Effectiveness of γ-Stimulation on Species

**Figure 3:**
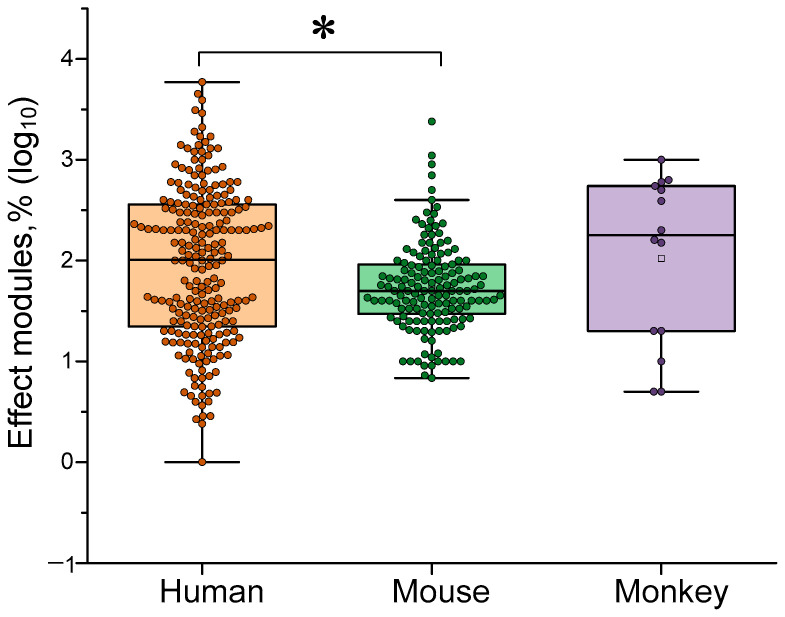
A comparison of the efficacy of gamma stimulation in different species: humans, mice and marmosets. The data are presented as median values and percentiles of 10%, 25%, 75%, and 90%. Each point corresponds to an experimental value taken from the literature. Each article may contain at least one experimental point. A total of 408 experimental values from 40 published papers were analyzed. *—*p* < 0.05, Kruskal–Wallis ANOVA with post hoc Dunn’s test.

In the first stage of our analysis, we analyzed published data on the general effectiveness of the γ-stimulation method in various animal species. The effects of γ-stimulation were most frequently assessed in humans (61% of the analyzed data) and mice (35%). Results obtained in rhesus macaques were described less frequently (4% of the analyzed data). Due to the high variability in the direction and amplitude of the effects, we opted for a logarithmic scale (log_10_), expressed in absolute terms. According analyzes γ-stimulation was generally more effective in humans than in mice and marmosets (see Figure 3). This may indicate the presence of interspecies peculiarities in the generation and control of the γ-rhythm. The cognitive abilities of humans and other animals differ significantly. These differences may also affect the effectiveness of γ-stimulation. Secondly, behavioral and cognitive tests are easier to implement in humans and survey methods can be used. Physiological measurement methods (skin microhemodynamics, etc.) are also much simpler to implement in humans as immobilization and/or anesthesia are not required. We believe that these two factors enable us to obtain more accurate results in humans than in other animals.

### 4.2. Dependence of the Efficiency of γ-Stimulation on the Type of Stimulus

**Figure 4:**
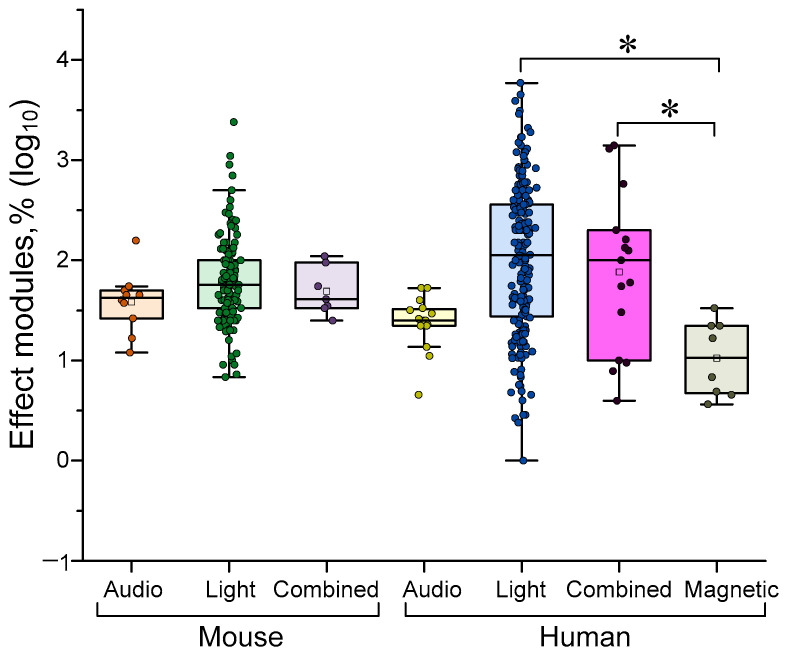
A comparison of the efficacy of gamma-stimulation methods for different species. Data are presented as median values and percentiles at the 10th, 25th, 75th and 90th percentiles. Each point corresponds to an experimental value taken from the literature. Each article may contain at least one experimental point. A total of 393 experimental values from 38 published studies were analyzed. *—*p* < 0.05, Kruskal–Wallis ANOVA with post hoc Dunn’s test.

As previously mentioned, the main methods of delivering gamma stimulation are light (presentation of a visual stimulus or exposure to a flickering light source), sound stimuli, or a combination of both. As the effectiveness of γ-stimulation differs between humans and animals, we analyzed all quantitative data separately for humans and mice, as these are the most accessible and popular methods. Magnetic stimulation can sometimes be used in humans. The results of a comparison of different γ-stimulation methods are shown in Figure 4 below.

In mice, no difference in the effectiveness of gamma stimulation with light, sound or a combination of the two. Stimulation with sound stimuli was generally more effective than magnetic stimulation (see Figure 4). Light stimulation tended to be more effective than sound stimulation without light.

**Figure 5:**
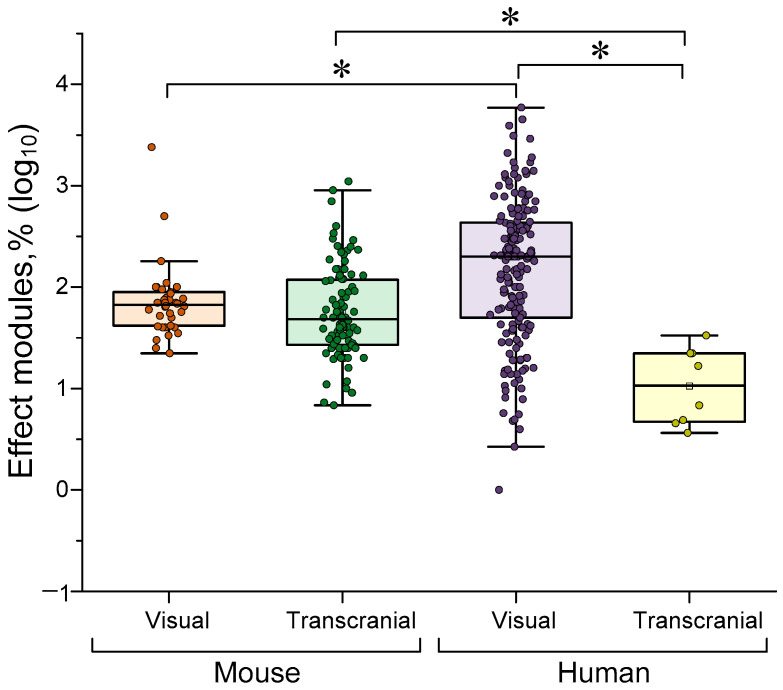
A comparison of the efficacy of different methods of exposure to light γ-stimulation. The data are presented as median values and percentiles of 10%, 25%, 75% and 90%. Each point corresponds to an experimental value taken from the literature. Each article may contain at least one experimental point. A total of 323 experimental values from 38 published studies were analyzed. *—*p* < 0.05, Kruskal–Wallis ANOVA with post hoc Dunn’s test.

Light stimulation comes in two forms: visual stimulation via the visual analyser or transcranial stimulation. The second method involves irradiating selected brain regions with infrared radiation, which can penetrate cranial tissue, or with visible light through a transparent window created in the skull (for mice only). A comparison of the effectiveness of light γ-stimulation by exposure method is shown in Figure 5 below.

No differences were found between transcranial and visual γ-stimulation in mice. This lack of difference may be due to the paucity of visual stimulation for rodents, which typically consists of only periodic blinks. In humans, visual stimulation is significantly more effective than transcranial stimulation (~10 times). Visual γ-stimulation is almost ~5 times more effective in humans than in mice. The differences may be due to methodological considerations. Firstly, the skull thicknesses of humans and mice differ, making transcranial stimulation difficult to achieve in humans. Secondly, as noted above, visual stimulation in mice is typically limited to simple periodic blinks. More complex (and likely more effective) stimuli may be used in humans.

**Figure 6:**
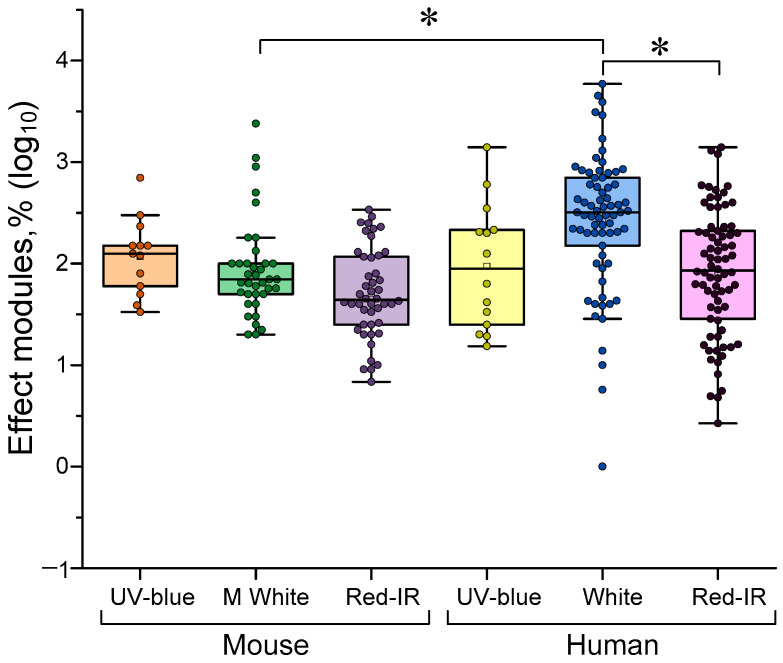
The efficiency of light γ-stimulation depending on the wavelength range used: 360–470 nm (UV–blue), 500–600 nm (white) and ≥600 nm (red–IR). The data are presented as median values and percentiles of 10%, 25%, 75% and 90%. Each point corresponds to an experimental value taken from the literature. At least one experimental point can be taken from each article. A total of 323 experimental values from 38 published studies were analyzed. *—*p* <0.05, Kruskal–Wallis ANOVA with post hoc Dunn’s test.

Three types of LED are typically used: broadband white with peaks in the 500–600 nm range, short-wavelength UV-blue (360–450 nm) and red/IR (600–1000 nm and above). There is evidence that the effectiveness of γ-stimulation depends on the wavelength of the stimulating light. Our analysis confirmed this concept. Light with a central wavelength of 500–600 nm was most effective for humans compared to deep red (>600 nm) and IR light (see Figure 6). Interestingly, visual stimulation with conventional white light is less effective for mice than for humans. The reduced efficacy of blue light for gamma stimulation compared to white light is consistent with classical concepts [36,72]. However, traditionally, white and far-red colors are considered to be equally effective for γ stimulation. Our data indicate the greater efficacy of white light (sometimes containing near-red) compared to red. This phenomenon is easily explained by the higher sensitivity of the human eye to yellow-green light compared to red. Moreover, this sensitivity is expressed not only at the quantum level of the eye’s receptor pigments, but also in the sensitivity of the central nervous system and its responses to color stimulation [73,74,75].

**Figure 7:**
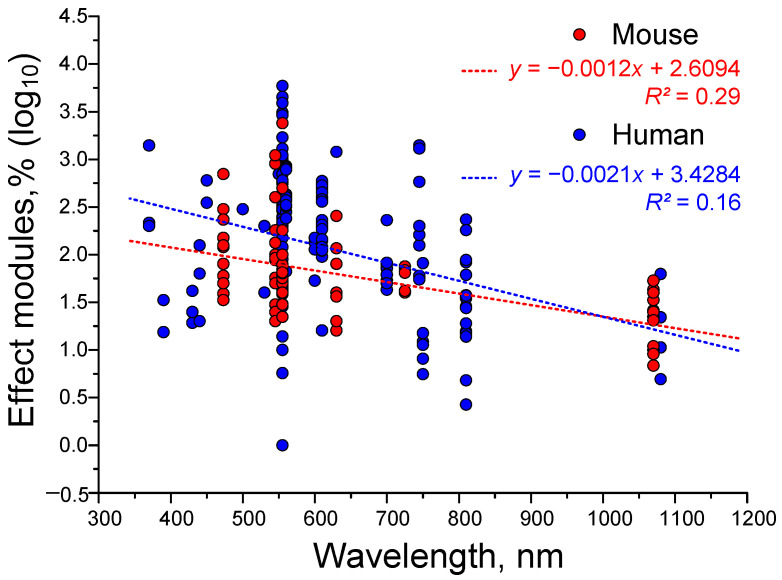
The dependence of the efficiency of light γ-stimulation on wavelength in humans (blue dots) and mice (red dots). Each dot represents an experimental value taken from the literature. Each article may contain at least one data point. The dotted lines correspond to straight lines of approximation for humans (blue) and mice (red). A total of 323 experimental values from 38 published papers were analyzed.

To further illustrate the relationship between gamma stimulation effectiveness and light wavelength, we attempted to plot straight lines of approximation (see Figure 7). We detected a trend of decreasing gamma stimulation effectiveness with increasing wavelength. This trend was observed for both mice and humans. However, the R^2^ value was too small to consider the ‘wavelength-effectiveness’ relationship significant. Perhaps we failed to detect a significant correlation because wavelength is not the only key factor. The age of the subjects is also very important. In the next step, we attempted to assess the dependence of gamma stimulation effectiveness on age.

### 4.3. Dependence of the Effectiveness of γ-Stimulation on Age

**Figure 8:**
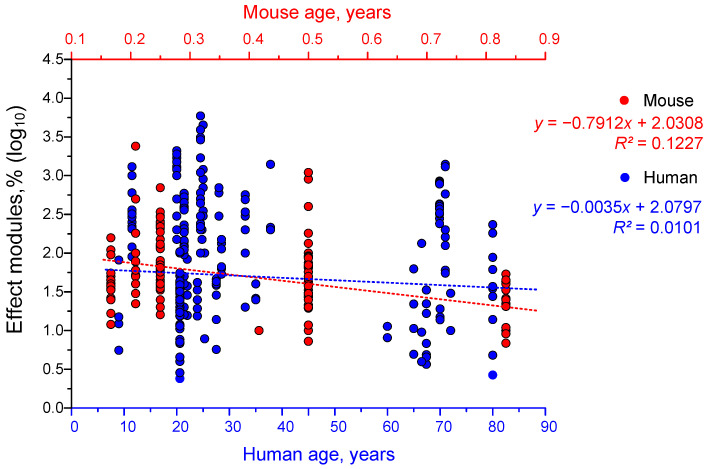
The dependence of the effectiveness of γ-frequency light stimulation on age in humans (blue dots) and mice (red dots) in years. Each dot corresponds to one experimental value taken from the literature. Each article may contain at least one experimental data point. The dotted lines correspond to the straight lines of approximation for humans (blue) and mice (red). A total of 393 experimental values from 38 published papers were analyzed.

We observed a weak trend towards decreased effectiveness of gamma stimulation with increasing age in mice (Figure 8). In humans, however, the effectiveness of gamma stimulation decreased only very slightly with age.

**Figure 9:**
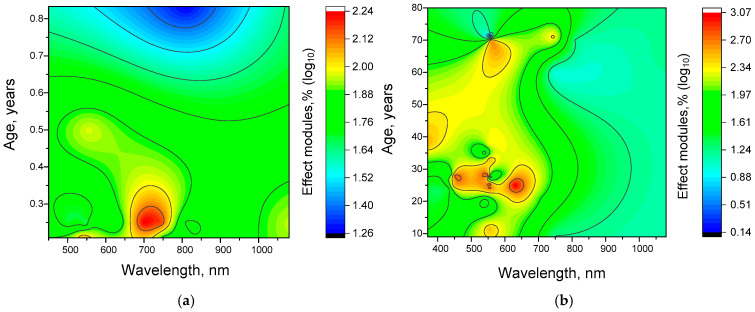
Three-dimensional maps of the dependence of the effectiveness of gamma stimulation effects on the wavelength of the stimulation light (abscissa) and age (ordinate). Reconstructions were constructed using the Kriging correlation method based on the points shown in Figure 4 and Figure 5. (**a**) Mouse, (**b**) Human.

We next constructed 3D maps to determine the combined effects of age and wavelength on the efficacy of γ-light stimulation in humans (Figure 9a) and mice (Figure 9b).

The analyses performed showed significant differences in the distribution patterns of γ-stimulation efficiency, which depended on both age and wavelength. For mice, a fairly uniform region of high efficiency was found at frequencies of 500–600 nm and at an age of 4–5 months (see Figure 9). We believe that this high homogeneity is due to the consistency of the experimental studies and the methodological conditions. There are also additional regions of high efficiency. The first of these is found at an increased age (6 months) and at short wavelengths (below 500 nm). The second is at 2 months and in the infrared (≥1000 nm) range.

The following trends were detected in the case of humans (Figure 9). Firstly, there was a slight increase in the effective wavelength, from 500–600 nm to 600–700 nm. This is consistent with literature data on the increased effectiveness of gamma stimulation with increasing wavelength. Secondly, there is an increase in the effectiveness of gamma stimulation with decreasing age. This can be explained by increased brain plasticity in young people. Thirdly, there is a shift in effective wavelengths towards the short-wave region at the age of 40–50 years. This trend has not yet been clearly explained. One possible explanation is that it is a consequence of the complex nature of age-related changes in color perception [76]. In particular, sensitivity to green-blue, blue-green, and green colors decreases relatively quickly with age. However, the sensitivity of cones in the blue-violet range decreases more slowly with age [77]. It is possible that the shift in effective wavelengths towards the blue-violet region in middle age is due to the preservation of visual sensitivity in this range compared to others.

To assess the statistical significance of the observed dependencies of the effect on age and wavelength, we calculated z-scores for each effect value. According to our calculations, for mice, the log_10_ threshold for effects that differed significantly in z-score was 2.1. For humans, this value was slightly higher at 2.4. In both cases, the orange and red colors on the 3D maps correspond to areas with a z-score ≥ 2 (*p* < 0.05). Consequently, the main patterns are statistically significant.

Overall, the more complex relationship between frequency, age and efficiency in humans compared to mice may be due to humans’ longer lifespan.

### 4.4. Dependence of the Efficiency of γ-Stimulation on the Frequency of Stimulating Action

**Figure 10:**
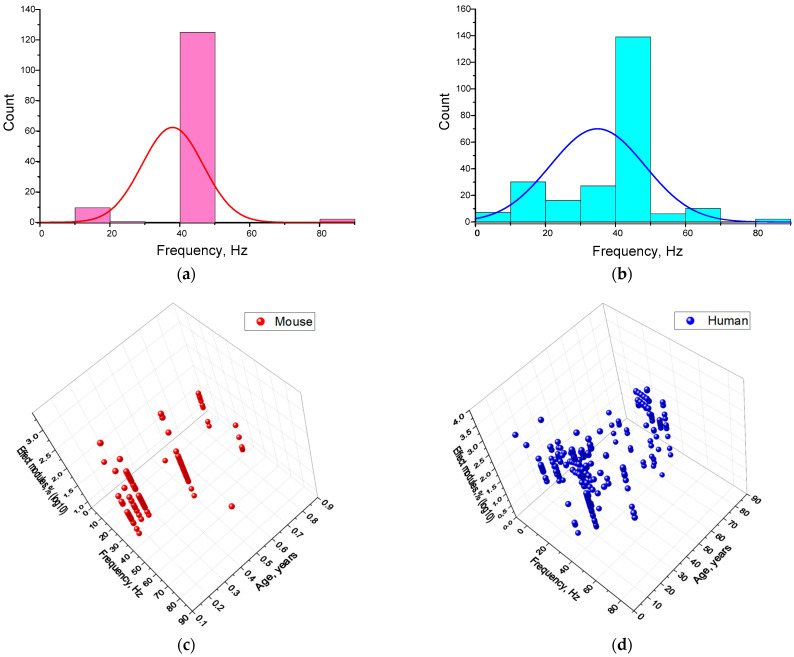

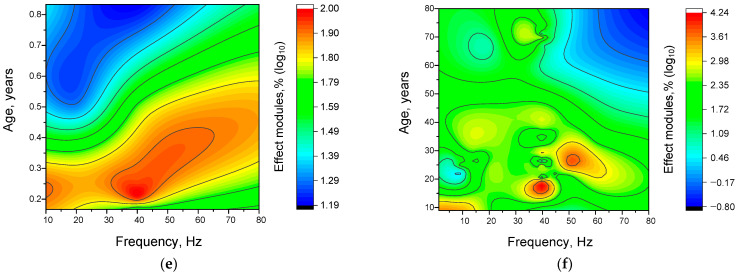
The efficiency of γ-stimulation effects depends on the frequency of stimulation. Frequency distributions used for γ-stimulation in mice (**a**) and humans (**b**). The bar graphs show the actual, discrete distribution of frequencies used in the analyzed studies. The lines represent the fitted theoretical normal distribution for each diagram. Three-dimensional scatter plots (**c**,**d**) and 3D maps (**e**,**f**) showing the dependence of the γ-stimulation effect modules (*z*-axis) on frequency (abscissae) and age (ordinate) for mice (**c**,**e**) and humans (**d**,**f**). Each point in graphs (**c**,**e**) corresponds to one experimental value from the analyzed studies. Each article may contain at least one experimental point. Three-dimensional reconstructions were constructed using the Kriging correlation method based on the points. A total of 408 experimental values from 40 published studies were analyzed (144 points for mice and 249 for humans).

By definition, gamma oscillations have a frequency of 30–60 Hz. Therefore, the majority of authors have assumed that γ-rhythm stimulation should be performed at this frequency. However, resonance phenomena, harmonic generation and other effects are also possible, meaning that the enhancement of γ-rhythm generation by rhythmic stimuli with frequencies outside of the 30–60 Hz range cannot be ruled out. The vast majority of analyzed results were obtained using a periodic stimulus at 40 Hz: over 90% and 60% of studies for mice and humans, respectively (see Figure 10a,b). Frequencies below 40 Hz (usually 10–20 Hz) and, in isolated cases, above 40 Hz (60–80 Hz) were used much less frequently. Studies on humans revealed a wider range of effective frequencies, particularly in the low-frequency region. It is possible that the limited range of effective frequencies in mice is due to the methodological features of animal experiments.

However, although many studies in mice investigated frequencies of 10 and 20 Hz, statistically significant results were not obtained. Therefore, these results are likely due to differences in species between mice and humans. Our results for humans are consistent with previous studies. In several studies, gamma oscillations were induced by stimulation at lower frequencies. Stimulation at any of the frequencies 10, 20, 40 or 80 Hz activates EEG rhythms across the entire frequency range. When stimulation occurs at 40 Hz, a response is recorded for 10, 20 and 80 Hz simultaneously. Stimulation at 20 Hz produces a response for both 10 and 40 Hz. Furthermore, stimulation at any frequency leads to the same doubling effect in both directions. This process is very similar to generating the second and subsequent even harmonics, which often occurs in periodic processes. Harmonic oscillations can also be generated in the EEG, as described in detail in the literature [54,78]. Furthermore, global harmonic synchronization between rhythms and the influence of heart rate and other systemic processes in the human body have been described [78]. Firstly, the generation of harmonics is possible. This phenomenon was first described using human EEG rhythms a long time ago. Secondly, rhythms do not exist in isolation in the brain; changes in the frequency response of one rhythm can cause changes in the frequency response of another. For instance, an alteration in the amplitude of the alpha rhythm can result in a change in the amplitude of the γ-rhythm [79].

Due to the limited and highly specific frequency ranges used for gamma stimulation, we did not analyze the relationship between gamma stimulation effectiveness and frequency separately. However, considering that the gamma rhythm frequency response can change with age, we decided to analyze the ‘frequency–age–effectiveness’ relationship (see Figure 10f). For mice, a fairly uniform pattern of increase in the effective γ-stimulation frequency from 10–40 Hz to 40–60 Hz with increasing age was obtained (Figure 10). Three regions of interest have been ground for human:

The first is a wide frequency range of 40–60 Hz and an age range of 15–30 years. This can also include scattered areas with frequencies of 10–30 Hz and ages of 20–40 years. This pattern is consistent with the literature on age-related changes in the central frequency of γ-rhythms in humans [72]. The presence of subgroups responding to high- or low-frequency signals can be explained by the presence of two γ-rhythms, one with fast frequencies of 45–70 Hz and one with slow frequencies of 25–45 Hz [80]. It is likely that the expression of fast and slow gamma rhythms varies between individuals. Individual differences could be reflected in the distribution of young and middle-aged people according to their effective stimulation frequencies.

The second region is narrow, with a central frequency of ~10 Hz and an age of ~10 years. The presence of this region is consistent with data on age-related changes in the expression and responsiveness of the gamma rhythm to periodic stimulation. At school age (7–12 years), there is an increase in the effective central frequency [81]. It is likely that, at younger ages, sensitivity to frequencies below 40 Hz, including 10 Hz, predominates. In general, data on the effectiveness of gamma stimulation in children is relatively scarce, making it difficult to draw conclusions about this subgroup.

The third is a narrowly localized region with a central frequency of around 40 Hz and an age of around 60 years. It is noteworthy that a ‘dip’ appears in the frequency range of 45–80 Hz for people over 60 years of age. This is consistent with data on the age-related decrease in GABA concentration in visual, sensorimotor, frontal and prefrontal cortex areas [82,83,84,85,86,87]. A decrease in the central stimulating frequency to ≤36 Hz has also been widely described in the literature [80,88]. The position of the third region in the 30–40 Hz range is partially consistent with these data.

To assess the statistical significance of the observed effect dependences on age and wavelength, we calculated z-score values for each effect modules value (as described above). In this case, z-scores > 2 were achieved for log10 effect sizes ≥ 1.95 (red) and ≥2.95 (yellow, orange, and red) for mice and humans, respectively. Therefore, the main relationships and patterns, shown in Figure 9, are statistically significant.

### 4.5. Dependence of the Efficiency of γ-Stimulation on the Target

**Figure 11:**
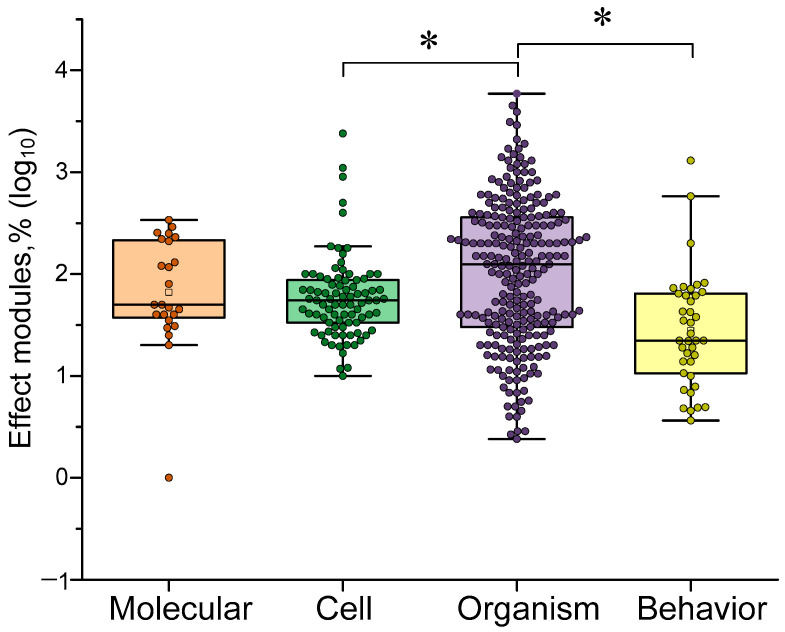
The comparison of sensitivity of different aspects (molecular, cellular, organismal and behavior) of γ-stimulation. The data are presented as median values and percentiles of 10%, 25%, 75%, and 90%. Each point corresponds to an experimental value taken from the literature. Each article may contain at least one experimental point. A total of 408 experimental values from 40 published papers were analyzed. *—*p* < 0.05, Kruskal–Wallis ANOVA with post hoc Dunn’s test.

In addition to investigating the effectiveness of γ-stimulation in relation to stimulus characteristics (frequency, type and color), we also examined which responses γ-stimulation enhances more or less strongly. First, we chose to study the sensitivity of different levels of organization in living things. γ-stimulation: molecular, cellular and organismal levels. We also identified behavioral and cognitive responses, as these are of particular interest. Overall, that γ-stimulation was most effective in influencing organismal responses (see Figure 11). These responses included changes in cerebral blood flow velocity, frequency response and EEG rhythm coherence. However, γ-stimulation was less effective in influencing behavioral responses and cognitive and behavioral test performance. This highlights one of the main issues with this approach: the loose connection between electrical changes recorded in the brain and actual changes in subjects’ quality of life.

Our results suggest that relying solely on EEG frequency response and rhythm synchronization assessments, without conducting additional cognitive testing, could produce false positive results. To obtain the most reliable results and draw adequate conclusions, we recommend that future studies adhere to the following research design. First, use EEG, the most sensitive method, to test a wide range of gamma stimulation conditions (frequency, amplitude, signal shape, wavelength for light or frequency for sound, etc.) and select several optimal ones. Then, conduct cognitive or behavioral studies to validate the EEG results and select an effective stimulation protocol.

No differences were observed between changes at molecular and organismal levels. This may indicate a strong link between molecular signaling processes and manifestations at the organismal level, such as EEG and microcirculation. The effects of gamma stimulation are less pronounced at the cellular level than at the organismal level. This may be due to the greater plasticity of the physiological responses studied (e.g., EEG and vascular response) compared to cellular-level processes (which require time for signaling and functional proteins to be expressed) [89,90].

### 4.6. Dependence of the Effectiveness of γ-Stimulation on Pathology

**Figure 12:**
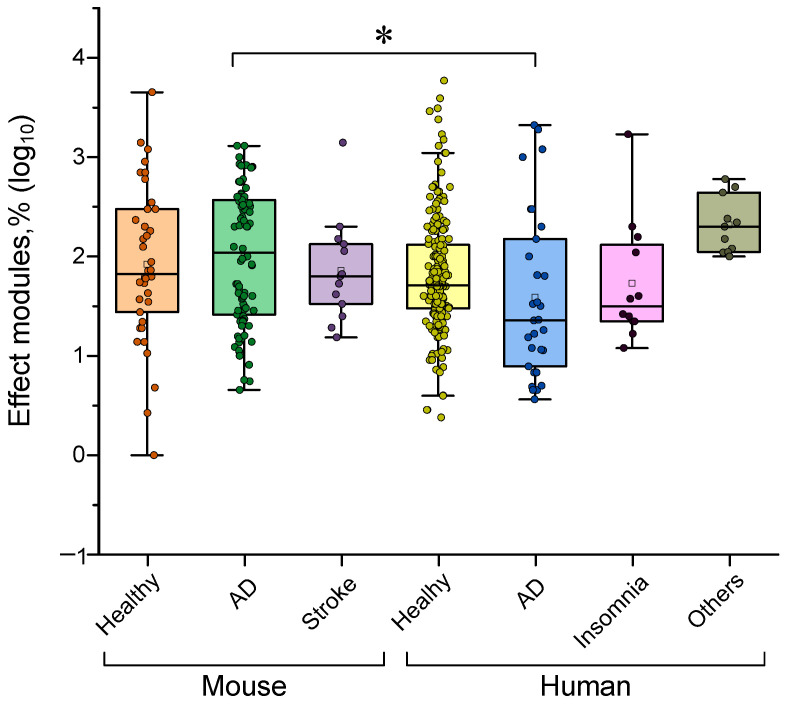
γ-stimulation efficiency in cases involving different species and the pathology under study. The data are presented as median values and percentiles of 10%, 25%, 75% and 90%. Each point corresponds to an experimental value taken from the literature. Each article may contain at least one experimental point. A total of 393 experimental values from 38 published studies were analyzed. *—*p* < 0.05, Kruskal–Wallis ANOVA with post hoc Dunn’s test.

Next, we evaluated whether the effectiveness of γ-stimulation differs between healthy subjects and individuals with pathological conditions. Based on the analyzed data, we selected the following groups: healthy subjects; patients with Alzheimer’s disease (AD); AD model animals; a stroke model; and patients with insomnia or other mental disorders. All analyzed literature data were divided by species: mice or humans. Overall, γ-stimulation was found to be equally effective in healthy humans and mice (see Figure 12). This suggests that animal models are suitable for fundamental research into the effects of gamma rhythm modulation. When considering each species separately, the effectiveness of γ-stimulation was found to be the same for healthy animals or subjects as for pathological cases. Animals with AD or a stroke, as well as intact animals, responded equally effectively to γ-stimulation. In patients with AD, insomnia and dementia, the effectiveness of gamma stimulation was comparable to that observed in healthy volunteers. This result further confirms the validity of the γ-stimulation approach in correcting pathological conditions.

However, there is a caveat. Significant differences in effectiveness are observed when evaluating the response to γ-stimulation in mice and humans with AD. Mice with AD showed a more pronounced response to γ-stimulation than patients with the disease did. Therefore, caution and accuracy are required when extrapolating data obtained from the AD mouse model to patients with the disease. Perhaps differences in sensitivity to γ-stimulation between species have slowed down research in this area. To obtain results that can be reliably transferred to humans, it is likely that additional pathology models must be created and/or identified. These new models should respond to γ-stimulation in a manner similar to that observed in real patients.

## 5. Mechanisms of γ-Stimulation Action

In order to understand the mechanisms of γ-stimulation, it is necessary to consider the external signal pathway, synchronization and subsequent effects in sequence. We will examine synchronization in detail, using visual stimulation as an example.

### 5.1. Visual Stimulation

The visual analyzer plays a direct role in the brain’s γ-activity. Electrophysiological data obtained from macaques showed that induced gamma synchronization between the primary visual cortex (V1) and the higher visual cortex (V4) facilitated the transmission of sensory signals for motor reactions, reducing reaction time [91]. It is well established that rhythms of electrical brain activity can differ in both frequency and localization [92]. The discovery of three types of narrow-band gamma rhythm in the primary visual cortex (V1) contributes to a deeper understanding of how information is processed by gamma oscillations. These three rhythms, known as low-frequency (25–40 Hz), mid-frequency (40–65 Hz) and high-frequency (65–85 Hz) γ-rhythms, process signals with different spatial frequencies and therefore convey distinct aspects of visual information in response to the original stimulus. However, they are actually generated by different neural networks [93]. In the field of multisensory cross-talk, it is widely believed that cross-modal matching of sensory signals depends on direct interactions between sensory cortical areas. Studies of human EEG with γ-stimulation (40 Hz) have confirmed that synchronized γ-oscillations in the cerebral cortex modulate multisensory communication during a visual–tactile matching task [94].

It has been demonstrated that brighter light results in greater rhythm synchronization [72,95,96]. Higher-amplitude light energy can cause more significant changes in the electrochemical properties of retinal photoreceptors and nerve conduction in the visual pathways [97].

Additionally, stimulation with longer waves elicited a stronger response than short-wave stimulation [98,99]. The cones in the retina are responsible for color vision, and in humans, cones sensitive to long wavelengths are denser than those sensitive to medium and short wavelengths [100,101]. Red light primarily excites medium- and long-wavelength cones (M- and L-cones), whereas white light also activates short-wavelength cones (S-cones). Studies have shown that stimulating L-cones strongly enhances the gamma rhythm in the visual cortex [102], whereas stimulating S-cones with shorter wavelengths fails to do so [103,104].

In practice, longer wavelengths are generally more effective at stimulating γ-rhythms [96]. However, the difference between white and red light flashes for young adults is insignificant. Moreover, a study involving older adults revealed no differences [72]. The lack of difference in sensitivity to white and red stimulation in older adults can be explained by two factors. Firstly, the spectral intensity of the white light used in this study consists mainly of longer wavelengths. Secondly, the age-related decrease in lens transparency reduces transmittance for shorter wavelengths [105,106]. These factors result in a decreased proportion of shorter wavelengths in white light. These factors may have influenced the results by reducing the difference in the effect of red and white light on γ-stimulation. Consequently, white light (peaking at 612 nm), which has a longer wavelength, may be as effective as red light (peaking at 614 nm) for γ-synchronization in older adults [15].

Visual γ-stimulation therefore primarily activates medium-wavelength (M) cones and long-wavelength (L) cones, meaning that longer-wavelength radiation has a greater effect on the γ-rhythm. This effect also depends directly on light intensity.

### 5.2. Modulation of GABAergic Interneuron Activity

The main type of neuron in the neocortex is the pyramidal neuron, accounting for 80–90% of all neurons. Those activated by gamma-aminobutyric acid (GABA) play a special role. GABAergic interneurons constitute 10–20% of the total number of neurons in the hippocampus and neocortex, and are characterized by high heterogeneity [15,67]. Extensive subclasses of GABAergic interneurons have been identified in both the hippocampus and the neocortex based on the expression of biomarkers such as parvalbumin (PV+), somatostatin (SST+) and vasoactive intestinal peptide (VIP+). The main source of excitation of inhibitory neurons in the cerebral cortex is the thalamus [107]. Typically, GABAergic neurons receive the greatest number of signals from thalamic areas that are most functionally connected to their own area of the cerebral cortex. For instance, in the primary auditory and visual cortices, inputs from the medial and lateral geniculate bodies of the thalamus, respectively, elicit excitatory postsynaptic currents in PV+, SST+ and VIP+ interneurons [107,108]. These areas are involved in visual and auditory γ-stimulation.

**Figure 13:**
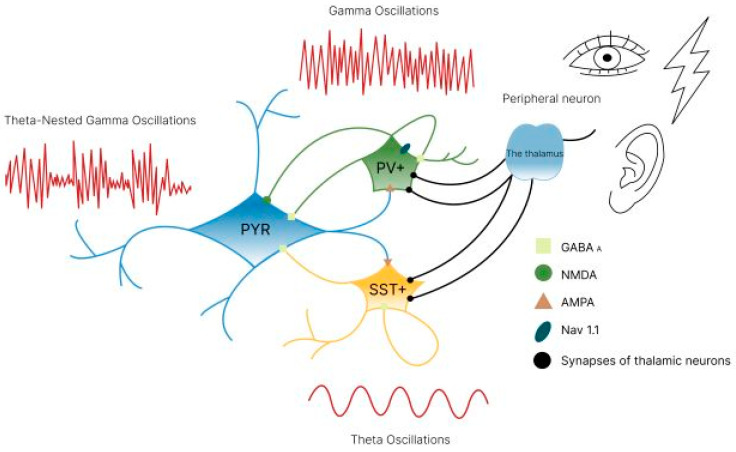
The mechanism of γ-oscillations. The functional connection between pyramidal neurons (PYR) and inhibitory interneurons expressing parvalbumin (PV+) and somatostatin (SST+) dynamically organizes synchronous oscillations in the θ- and γ-ranges via γ-oscillation mechanisms in the pyramidal neuron-interneuron network (PING) or in the interneuron network (ING) (references in the text).

In this way, the entire synchronization pathway of gamma oscillations can be traced using external stimuli. Sensory stimuli and behavioral responses are reflected in the thalamus, which subsequently modulates the activity of interneurons. This establishes a dynamic functional connection between inhibitory interneurons and pyramidal cells, enabling the latter’s activity to be controlled flexibly in real time. This connection ensures the short-term plasticity of synaptic inhibition and consequently endogenous γ-oscillations in response to external sensory input (see Figure 13).

Furthermore, PV+ interneurons are responsible for generating γ-oscillations, whereas SST+ interneurons generate θ-oscillations. These two types of oscillation typically exist together as θ-nested gamma oscillations, where θ-rhythms are responsible for the temporal characteristics of the transmission of information in packets, and nested γ-rhythms are responsible for the transmission of information itself [109]. This ensures synchrony between brain regions, which is important for working memory, information retrieval, cognitive integration and processing information. Therefore, the correct activity of inhibitory interneurons is essential for forming certain brain states and ensuring healthy cognitive functioning, and this activity can be influenced by external stimuli.

At a higher organizational level, interneurons combine to form specific networks. Gamma rhythms, which are generated by interconnected GABAergic inhibitory interneurons, regulate the global balance of excitation and inhibition in the visual cortex [110,111,112]. Synchronous oscillations in the gamma range are generated by two types of interneuron network: PING (excitatory–inhibitory networks of neurons) and ING (purely inhibitory populations of neurons) [113,114]. The power and frequency of γ-oscillations are modulated by stimulus characteristics (e.g., direction, speed, contrast) [16,115].

The frequency of γ-oscillations is determined primarily by inhibitory postsynaptic currents induced by GABA receptors. In the ING network, interneurons mutually inhibit each other via GABA receptors (Figure 2) to quickly achieve synchrony in phase 0. During the PING mechanism, pyramidal cells induce rapid excitation of interneurons via AMPA receptors, which in turn ensures inhibition via GABA receptors and triggers γ-oscillations.

Meanwhile, brain properties such as resting GABA levels and the size of the affected cortical area also play a role. The relationship between the frequency used for visual synchronization and the excitability of GABAergic neurons is of interest, as this depends on the level of GABA production in the human body. It has been noted that, for the most effective synchronization with γ-rhythms in mature individuals, a frequency of about 32–34 Hz is necessary, which is less than 40 Hz [72]. This age-related decrease in the optimal flicker frequency may be due to a corresponding decrease in the central frequency of γ-rhythms in older individuals. The central frequency is the frequency at which power changes most strongly in response to external visual stimulation. Notably, even within a single area (the hippocampus), the presence of two γ-rhythms with distinct frequencies is possible: one fast (45–70 Hz) and one slow (25–45 Hz) [80]. These rhythms can be stimulated differently by the same stimulus, and their response depends on stimulus orientation, contrast, drift velocity and amplitude [80]. With increasing age, the amplitude of fast gamma oscillations decreases more significantly than that of slow ones [88]. According to some studies, the central frequency of the stimulus increases monotonically with the intensity of the visual stimulus: visual contrast [116,117,118], movement speed [16,115] and positively correlates with the GABA level in the visual cortex [116,118]. Moreover, the central frequency of the stimulus increases as the tonic excitability of GABAergic inhibitory interneurons increases [119,120]. The central frequency gradually decreases with age (by 0.16 Hz per year in the high γ-activity range of 36 Hz and above and by 0.08 Hz per year in the low γ-activity range below 36 Hz) [80,88]. It has been shown that GABA levels decrease in the visual, sensorimotor, frontal and prefrontal areas of the cortex with age in humans [82,83], which may impair inhibitory intracortical circuits [84,85,86,87].

Studies [120,121] have documented age-related differences in central frequency during visual γ-stimulation in humans. This age-related decrease in central frequency may be associated with a reduction in the excitability of GABAergic inhibitory interneurons, which is caused by a decrease in GABA production [83,122]. Thus, the effect of γ-stimulation is realized through GABAergic interneurons and depends on the level of GABA production, which determines the excitability of these neurons and the central frequency of the response to external stimuli. Moreover, as people age, the central frequency may decrease, meaning that the flickering rhythm for effective γ-stimulation must be selected on an individual basis. This rhythm may ultimately differ from 40 Hz.

### 5.3. Acetylcholine Mechanism of γ-Oscillation Activation

It has been established that including cognitive tasks such as attention, memorisation, counting and searching for inconsistencies in gamma stimulation sessions promotes the spread of the gamma rhythm to additional areas of the brain, including the frontal and deep lobes such as the hippocampus [123,124]. Additionally, coherence and cortico-cortical interactions between different brain regions are enhanced [125,126,127]. These effects can be explained by the fact that, in addition to GABAergic transmission (used in both ING and PING, see above), the PING network with asynchronous excitation can generate γ-oscillations in response to impulses of acetylcholine (ACh) through muscarinic ACh receptors (mAChRs). This ensures the network can adapt dynamically during tasks that require high levels of concentration [128]. Signal detection in behavioral attention tasks depends on cholinergic-driven γ-oscillations in the frontal cortex, to which both mAChRs and nAChRs make a significant contribution [129]. Therefore, cognitive attention tasks enable the involvement of additional neural populations (PING) and the spread of γ-oscillations induced by external stimuli to be much broader and deeper.

In addition, N-Methyl-D-aspartic acid (NMDA) receptors are involved in the coherence and amplification effects of the synchronized γ-signal. When external stimuli induce γ-synchronization in the network, functional NMDA receptors are activated in the excitatory feedback synapses between CA1 pyramidal cells and parvalbumin-positive (PV+) interneurons. This improves and stabilizes neuronal assemblies [130,131]. NMDA receptors in PV+ cells generate a relatively slow postsynaptic current and are involved in controlling spontaneous and induced γ-oscillations. This explains why NMDA receptor blockers (ketamine, MK-801, phencyclidine, etc.) affect γ-oscillations [130,132]. In addition, the involvement of sodium channels Nav1.1 (Figure 13), a subunit of voltage-gated sodium channels, is important. t is expressed in interneurons following neddylation to maintain the excitability and the excitatory/inhibitory balance of GABAergic interneurons [131,133,134]. Nav1.1 has been observed to promote behavior-dependent γ-oscillations [134].

Thus, to expand γ-synchronization areas, it is important to use a cognitive task (see below) alongside visual or auditory stimulation. This activates a parallel mechanism associated with γ-oscillation activation and acetylcholine. Voltage-gated sodium channels are also involved in this process. NMDA receptors stabilize and enhance the resulting neural coalitions (assemblies), ultimately achieving interareal coherence.

### 5.4. Modulation of Neural Network Activity

The current neuroscientific paradigm is that cognitive tasks are not performed by discrete brain regions operating in isolation, but by networks consisting of several such regions that are said to be ‘functionally connected’ [135,136,137].

As mentioned above, gamma oscillations coordinate communication between different parts of the cortex, ensuring the brain’s temporal and spatial connectivity [11,138]. The set of interconnected brain regions forming large-scale networks varies depending on the cognitive function [139,140]. Large-scale brain networks are identified by their functions and grouped into self-organizing coalitions. The number and composition of these coalitions vary depending on the algorithm and parameters of the problem being solved [135,141,142,143].

Surprisingly, despite the significant importance of understanding that the brain functions as a network and that gamma oscillations mediate communication between different brain regions, we found no studies in the literature on the effects of gamma stimulation on the activity of known neural networks. The few literature sources we were able to find demonstrating the possibility of such an effect do not yet provide a comprehensive picture and are quite sparse. We would like to draw the attention of researchers to this extremely interesting area of research. Here we present information on three of the most studied neural networks and our findings on their relationship with γ-stimulation.

#### 5.4.1. Basic Modes of Brain Function and γ-Stimulation

Abnormalities in activity in various networks are associated with neuropsychiatric disorders such as depression, Alzheimer’s disease, autism spectrum disorders, schizophrenia, ADHD, and bipolar disorder [135,136,144,145,146]. Numerous functional magnetic resonance imaging (fMRI) studies have shown that Alzheimer’s disease is associated with atypical patterns of functional connectivity in large-scale brain networks [147,148,149,150,151,152,153,154,155]. During the default mode, various brain centers remain functionally connected, and the characteristics of network connectivity in this state can depend on factors such as gender, age, motor asymmetry profile, overall EEG pattern, and the presence of neuronal pathologies [156]. In particular, the functional connectivity of the brain’s default mode network is altered in Alzheimer’s disease. It is weakened in the hippocampus and medial lumbo-parieto-occipital regions [157,158]. Disruption of the default mode network is associated with the deterioration of cognitive functions in elderly individuals [159]. Recently, researchers have paid more attention to inter-network connectivity patterns in the insula to better understand the pathological changes in the organization of brain function in Alzheimer’s disease [160,161,162].

**Figure 14:**
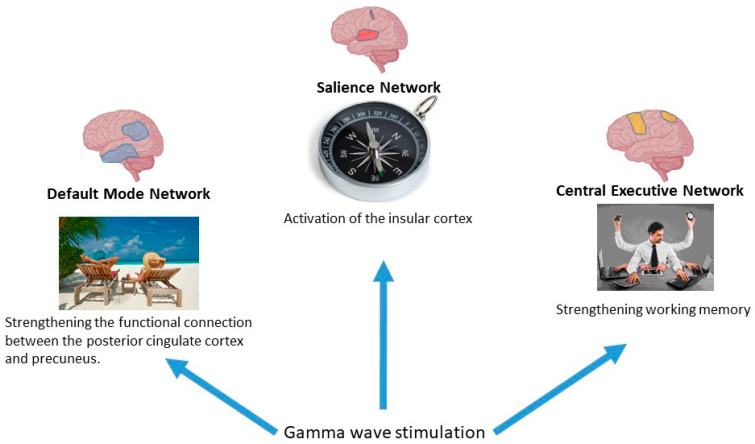
The most studied neural networks of the brain.

The three largest-scale networks that are responsible for cognitive functions are the default mode network, the executive network and the salience network. Together, these form the so-called ‘triple network model’ of Menon [11,135,147,163]. General neuronal network and γ-stimulation effects are shown at Figure 14.

#### 5.4.2. The Default Mode Network

Default mode network (DMN), also known as the neural network of operational rest, passive mode of brain function network, is a network of interacting brain areas that becomes active when a person is not performing any task related to the outside world, but rather is inactive, resting or ‘immersed in themselves’ [164].

The DMN includes several anatomically separate but functionally interconnected areas of the brain: the ventromedial prefrontal cortex; the dorsomedial prefrontal cortex; the lateral parietal cortex; and the posterior cingulate cortex with adjacent parts of the precuneus [165,166,167]. The DMN often includes the entorhinal cortex, which is associated with the hippocampus [168]. Significant connections have been shown between the DMN and the hippocampus and the thalamus, but not with the basal nuclei [169].

##### Functions of the DMN

Classically, the functions of the DMN are the processing of information about the internal world, including thoughts, memories, and emotions [170]. It also integrates past, present, and future [171,172]. It integrates information about the past retrieved from memory, signals perceived by the senses, and plans and images of the future. The DMN brings them together and facilitates understanding of what is happening in the current moment. It connects the points of the human life timeline and performs the following functions:

- Maintaining flexibility in thinking is the ability to switch from one task to another [173,174].
- Supporting autobiographical memory and integrating memories. It establishes deep connections with both the inner self and the surrounding world [175,176].
- Social thinking: thinking about the intentions and feelings of others [177,178].
- Supporting creative self-expression. The DMN has the ability to establish connections between individual areas of the brain, thus enabling unique associations and the development of a person’s individuality. This enables spontaneous and spontaneous action [179,180,181].
- Promotes clearer manifestation of vague memories. The DMN engages memory beyond the boundaries of attention, extracting information that cannot be retrieved by other means. [182,183].

Dysfunction of DMN or abnormal changes in the functional connections between brain regions have been found in people with depression, bipolar disorder, schizophrenia and other mental health conditions [184]. For example, altered activity and disrupted communication in the frontal cortex, hippocampus, and dorsomedial prefrontal cortex have been observed in depression [185,186,187,188]. Changes to these structures have been associated with the onset or intensification of rumination, the deterioration of cognitive function and the impaired processing of emotional stimuli. In patients with schizophrenia, hyperactivation of the DMN has been observed, which interferes with the processing of information from external stimuli [189,190].

##### Effects of γ-Stimulation on the Functioning of the DMN

Chronic (8 weeks daily) audiovisual γ-stimulation increased functional connectivity between the posterior cingulate cortex and precuneus (leading regions of the DMN) in patients with AD [191]. A decrease in cortical atrophy and an improvement in DMN function were also observed in patients with mild AD with long-term (3 months) audiovisual stimulation [192]. Results obtained using auditory stimulation at a frequency of 40 Hz showed an increase in neuronal activity in the parietal and prefrontal regions where the DMN is located [193,194]. In another study, audiovisual stimulation (3 months of daily stimulation) did not lead to changes in connectivity in the DMN, but significantly increased the average functional connectivity in the motor cortex of patients with mild AD [62].

#### 5.4.3. Central Executive Network

##### Central Executive Network Functions

This neural network provides a solution of task that requires concentration, planning or problem-solving needs to be completed [195,196]. It is in a reciprocal relationship with the DMN. It consists of the dorsolateral prefrontal cortex and the anterior cingulate cortex.

Some functions of the executive network include:

- Concentration and attention: maintaining focus on the task.
- Working memory involves retaining and manipulating information in the mind.
- Planning and control involves developing a strategy of actions and controlling their implementation.
- Inhibiting impulses and preventing rash actions.

##### Effects of γ-Stimulation on Central Executive Network

It has been demonstrated that the activation of working memory, one of the primary functions of the executive network, enhances the amplitude of gamma-band oscillations and strengthens their coherence across various regions of the cerebral cortex [197,198]. Furthermore, transcranial electrical stimulation of two distant areas within the gamma band has been shown to enhance working memory task performance [199]. These results suggest that γ-band coherence probably plays a significant role in maintaining working memory.

#### 5.4.4. Salience Network Executive Network

##### Salience Network Functions

This network comprises several structures, including the bilateral anterior insula, the dorsal anterior cingulate cortex, and three subcortical structures: the ventral striatum, the substantia nigra, and the ventral tegmental area [200,201]. This network plays a pivotal role in monitoring the significance of both external stimuli and internal brain activity [202,203,204]. In particular, salience network helps direct attention by identifying important biological and cognitive events [205]. Salience network regulates the activity of the aforementioned two networks, activating them depending on the environment.

##### Effects of γ-Stimulation on the Salience Network

Studies have shown that visual stimulation at 40 Hz activates the cortex, the hippocampus and the insular cortex, which is part of the salience network [123]. Thus, the enhancement of cognitive abilities during gamma synchronization is realized through modulation of the activity of various brain networks responsible for different cognitive functions, such as DMN, the central executive network, etc.

### 5.5. Activation of Neurogenesis

Long-term exposure to 40 Hz flickering light (one hour per day for 30 days) has been shown to significantly improve spatial learning and neurogenesis in the dentate gyrus of the mouse hippocampus, with no detrimental behavioral side effects observed [206]. As discussed above, this effect is based on the activation of PV+ interneurons and the GABAergic support of immature neurons. This demonstrates the long-term benefits of this treatment for neurological diseases. Mechanistically, the stimulation did not alter regional microvascular blood flow, but it did significantly increase PV+ interneuron excitability, GABA levels and inhibitory transmission in the dentate gyrus of the hippocampus. Blocking GABA receptors abolished the improvements in spatial learning and neurogenesis. These data demonstrate that long-term exposure to 40 Hz flickering light improves spatial learning through PV-dependent adult neurogenesis, a process that requires elevated GABA levels to support neurogenesis in adult mice.

In adult mice, neurogenesis predominantly occurs in the granule cell region of the hippocampus and the subventricular zone of the lateral ventricles. The formation of new neurons in the granule cell region during adulthood is critical for learning and memory formation. Neurogenesis occurs predominantly in the granule cell region of the hippocampus and the subventricular zone of the lateral ventricles in the adult mouse brain. The formation of new neurons in the granule cell region during adulthood is critical for learning and memory formation [207,208,209,210].

The precise regulation of neurogenesis in the hippocampus largely depends on localized neural circuits that integrate individual experience, neuronal activity, and the regulatory mechanisms of neurogenesis [211]. Within these circuits, two main types of GABAergic interneuron play a vital role in supporting the development of new neurons. The first are rapidly excitable PV+ interneurons, and the second are SST+ interneurons. Both types of interneuron inhibit granule cell dendrites. PV interneurons are typically located closer to the granule cell layer, providing lateral and reciprocal inhibition and promoting continuous GABA release [212,213]. Synaptic connections between PV interneurons and neonatal neurons can be observed as early as seven days after birth, i.e., earlier than connections established by SST+ interneurons [213]. Previous studies have shown that optogenetic inhibition of PV+ interneurons, but not SST+ interneurons, effectively suppresses neurogenesis in the granule cell region [214].

The activation of PV+ interneurons can serve as a sensor of circuit activity, providing local signals that activate various components in the hippocampal neurogenic niche. Notably, GABA influences immature neurons by increasing their resting potential through its side effects and subsequent synaptic release, making them more receptive to action potentials [215,216,217,218]. This excitatory effect of the inhibitory neurotransmitter GABA on neonatal neurons is explained by the high expression of NKCC1 chloride channels, which depolarize the chloride ion equilibrium potential in neonatal neurons, particularly during the first three weeks after birth [213,219,220].

In order for inhibitory transmission to support neuronal maturation in the dentate gyrus of adult mice, GABA release must be enhanced by the activation of interneurons. This can be achieved through prolonged exposure to 40 Hz flickering light [206]. Thus, one of the key effects of PV+ GABAergic neuron activation is the stimulation of neurogenesis in the hippocampus, which is certainly beneficial for the long-term positive effects of γ-synchroenzyme.

### 5.6. Adenosine Hypothesis

An alternative mechanism by which γ-stimulation exerts its effects is the enhancement of adenosine production [221]. The main sources of this production are glutamatergic and GABAergic neurons. In this case, however, astrocyte activity does not play a decisive role in the production of extracellular adenosine. Additionally, an increase in the calcium signal was observed in the V1 area of mice. Furthermore, the calcium channel blocker feldopidine (10 mg/kg, intraperitoneally) almost completely eliminated the increase in extracellular adenosine levels induced by 40 Hz flicker, as compared with the placebo group. This is an important consideration when using γ-stimulation in humans, since many drugs affect calcium channels and can therefore reduce the effectiveness of the stimulation.

Light-induced neural activity (e.g., spiking activity or γ-oscillations) is particularly energy-consuming and requires coordinated mitochondria. It also results in intense oxygen consumption and hemodynamic changes. This is evidenced by the dependence of signal frequency responses in the cerebral cortex on blood oxygen concentration [222,223]. As adenosine is the main signaling molecule resulting from increased energy expenditure [224,225,226], it is highly likely that light-induced neural activity represents a form of energy-consuming neural stimulation resulting in rapid adenosine production. Extracellular adenosine, which is closely associated with energy metabolism, can act as a modulator of neurotransmission and synaptic plasticity, as well as regulating metabolic homeostasis, motility, proliferation and vasodilation in the brain [227,228,229].

Thus, prolonged exposure to a single frequency (40 Hz) is extremely energy-consuming and activates energy metabolism with the production of significant amounts of adenosine, which has significant biological effects (hypnotic effect, increased neuroplasticity, modulation of neurotransmission, vasodilation, etc.) [230].

### 5.7. Effect of γ-Stimulation on Microglia and Inflammation

The occurrence of abnormal γ-oscillations alongside neuroinflammation indicates disruption to the microenvironment of the CNS neural network. In the CA3 region of the hippocampus, aged *nfkb−/−* mice, which exhibit low-grade sterile inflammation with aging, demonstrate reduced γ-frequency oscillation power [231]. Various levels of neural network dysfunction are caused by microglial activation, primarily due to the excessive release of reactive oxygen species and nitric oxide via Toll-like receptors (TLRs), rather than proinflammatory cytokines [232]. IFN-γ-activated microglia enhance the expression of inducible nitric oxide synthase (iNOS) and subsequently produce large amounts of nitric oxide, leading to a decrease in γ-frequency oscillations and an increase in neuronal death in hippocampal slices in situ [233]. Combined with lipopolysaccharide and interferon-γ stimulation, hippocampal cells exhibit a significant loss of PV+ neurons and reduced or absent γ oscillations [233]. Rodent studies and clinical trials have demonstrated that anti-inflammatory therapy can restore gamma rhythms and enhance cognitive function [234,235]. These results highlight the sensitivity of γ oscillations to disruption of CNS homeostasis and the role of microglia, the brain’s resident immune cells that provide dynamic surveillance, in maintaining γ oscillations in disorders associated with various microglial functions, such as the immune response, reactive oxygen species production and synaptic remodeling.

Visual stimulation at 40 Hz has been shown to significantly reduce neuronal loss in the visual cortex (V1), the CA1 region of the hippocampus, the somatosensory cortex and the cingulate cortex in mice with neurodegeneration. It has also been shown to reduce the loss of CA1 excitatory neurons in mice with ischemic stroke. However, the mechanism of this effect is not fully understood. Studies have shown that gamma stimulation suppresses the expression of inflammatory genes and reduces DNA damage. A γ-stimulation has also been shown to protect mice with P301S and CK-p25 mutations from severe neuronal loss and brain atrophy [62]. Interestingly, restoration of neuronal survival following ischemia does not appear to depend on changes in cerebral blood flow or the response of microglia, suggesting that visual stimulation may directly affect neurons.

The immune function of the brain is generally attributed to microglia, the brain’s primary phagocytes. This is because they engulf pathogens and release cytokines and other extracellular signaling molecules that maintain neuronal health [236,237,238]. In a healthy adult brain, the processes of microglia move dynamically to explore the surrounding synapses. They acquire an elongated shape in order to scan the environment and monitor neuronal activity (hyperbranching). Upon detecting pathogens, the microglia rapidly changes their phenotype. They pass through intermediate stages where their processes shorten and their bodies enlarge (hypobranching), transforming into an amoeboid form that may lack processes entirely [237].

Depending on the nature of the pathological condition and the resulting changes to the microenvironment, different types of microglia can be activated [239]. The two extreme types are polarized M1 and M2 cells, as described for peripheral macrophages [240]. M1 microglia play a vital role in combating infection and injury, the majority of factors they secrete are toxic to neuronal cell cultures. Conversely, in the presence of IL-4, IL-13 or IL-10, microglia differentiate into M2 or alternative microglia. These are characterized by the expression of IL-10, heparin-binding lectin (Ym1), the cysteine-rich protein FIZZ1 and arginase 1 [241]. Although M1 microglia plays an important role in combating infection and injury, most factors secreted by M1 microglia are toxic to neuronal cell cultures. On the other hand, in the presence of IL-4, IL-13, or IL-10, microglia differentiate into M2 microglia or alternative microglia, which are characterized by the expression of IL-10, heparin-binding lectin (Ym1), the cysteine-rich protein FIZZ-1, and arginase 1 [241]. The anti-inflammatory M2 phenotype exhibits neuroprotective properties and is involved in resolving inflammation, phagocytosis, and tissue repair.

The M1/M2 microglial activation paradigm is being studied more and more in the context of neurodegenerative and neurological diseases, where an imbalance towards the M1 form is observed [242,243,244]. Thus, normalizing the imbalance between M1 and M2 differentiation has been proposed as a therapeutic target for treating CNS diseases associated with neuroinflammation. In this context, IL-10 combined with IL-13 has been shown to enhance the secretion of activin-A by microglia. Activin-A is a neuroprotective member of the TGF-β superfamily that promotes oligodendrocyte differentiation [245].

It was found that flickering at 20 and 40 Hz leads to different patterns of cytokine expression in healthy mice [67]. It was previously established that microglia change their morphology within a few minutes [246]. One hour of flickering at 40 or 20 Hz was found to alter microglial morphology consistent with their different functional roles, and also alter the transcription of genes controlling cytokine expression [67,236]. Flickering at 40 Hz caused a significant increase in somatic area (hypobranching) compared to 20 Hz, while 20 Hz caused a significant increase in process length and branching compared to 40 Hz. Thus, 40 Hz stimulation results in a more amoeboid morphology with fewer branches, consistent with ‘engulfing’ microglia phenotypes, while 20 Hz stimulation results in a more branched morphology with longer processes, consistent with ‘surveilling’ microglia phenotypes [37,236,247].

Flickering at 40 Hz increased the production of a wide range of cytokines. Some cytokines, including the anti-inflammatory cytokine IL-13 and the chemokine macrophage inflammatory protein-1β (MIP-1β) are secreted by microglia [248]. The γ-stimulation in microglia-deficient mice was found to increase tissue levels of cytokines such as macrophage colony-stimulating factor (M-CSF), which attracts microglia, and IL-10 with anti-inflammatory effects [67]. The authors conclude that these cytokines are produced by cells other than microglia. Microglia deficiency was induced using PLX3397 (an inhibitor of the colony-stimulating factor 1 (CSF1) receptor), which can kill macrophages [249]. The anti-inflammatory agent IL-10 on the one hand suppresses the production of TNF-α, but at the same time paradoxically slows the transformation of microglia from an activated phagocytic state to an intact one [250]. It can be assumed that this is the basis of the effect of γ-stimulation, which, on the one hand, reduces the severity of toxic inflammation, and on the other, activates the phagocytic activity of microglia (transferring it to the M2 state), facilitating the removal of amyloid plaques and tau protein.

IL-10 is a key cytokine that counteracts neuroinflammation. It suppresses the production of proinflammatory cytokines by microglia, thereby protecting astrocytes from excessive inflammation [251,252]. In neurons, signaling through the IL-10 receptor has been linked to increased cell survival and the regulation of neurogenesis in adults [253,254,255,256,257]. Therefore, IL-10 is a crucial mediator of intercellular interactions between microglia, astrocytes and neurons.

Notably, alongside the existing data suggesting that IL-10 regulates intercellular interactions within the CNS and immune system, there is a growing body of research indicating impaired IL-10 production or signaling in patients and animals with various neurological diseases, including neuropathic pain, multiple sclerosis, Alzheimer’s disease and Parkinson’s disease [255,258,259,260,261,262].

Furthermore, it was found that inhibiting nuclear factor kappa-light-chain-enhancer of activated B cells (NF-κB) or mitogen-activated protein kinases (MAPK) signaling pathways (which are responsible for synaptic and immune function) prior to exposure to flicker reduced cytokine expression; meanwhile, inhibiting NF-κB prevented the microglial response to 40 Hz flicker [67]. Taken together, these results suggest that flicker at different frequencies has different effects on microglia and cytokine transcription.

Another study found that exposing wild-type mice to γ-flickering light increased levels of cytokines such as IL-6 and IL-4, which enhanced microglial phagocytosis and increased the expression of chemokines, including macrophage colony-stimulating factor and interferon-gamma-induced monokines. This neuroimmune activation was mediated by γ-induced phosphorylation of proteins NF-κB and MAPK-dependent pathways in astrocytes [263,264,265].

The signaling molecules protein kinase R (PKR), c-Jun N-terminal kinase (JNK), and NF-κβ have been identified as likely participants in the regulation of TLR3-dependent IL-10 expression in astrocytes [266]. Therefore, it may be that astrocytes are able to realize the effects of γ-stimulation via the NF-κβ pathway mentioned earlier.

However, there is also an opposing point of view. In the context of Alzheimer’s disease, introducing IL-10 into the brains of transgenic mice with the amyloid precursor protein resulted in the accumulation of Aβ and a decrease in its phagocytosis by microglia [267]. The negative effect of IL-10 on Alzheimer’s disease was confirmed by the observation that the absence of IL-10 in transgenic mice with cerebral amyloidosis resulted in Aβ being phagocytized by activated microglia, leading to a reduction in Aβ levels in the mice’s brains and ultimately alleviating the disease’s symptoms to some extent [268]. Furthermore, the authors propose that a potential therapeutic strategy could involve restoring the balance of innate immunity in the brain by suppressing the action of key anti-inflammatory cytokines, such as IL-10, enabling the brain to return to a physiological state [268].

Phagocytic activity is enhanced. A reduction in the number of Aβ plaques in the visual cortex of 5XFAD mice exposed to γ-stimulation correlates with the morphological transformation of microglia [58]. Another study also found a reduction in the level of phosphorylated tau protein in mice with tauopathy with a similar transformation of microglia [62]. However, γ-oscillations induced by visual stimulation did not significantly affect the transition of microglia to a phagocytic state, the number of microglia, or markers of neuroinflammation in aged C57BL/6J mice [62]. Similarly, the response of microglia to gamma stimulation was limited in an animal model of ischemic stroke. This suggests that the effects of γ-stimulation on microglia may depend on health status or genetic predisposition [269]. This may be due to an age-related decrease in the excitability of GABAergic neurons and the level of GABA in the cortex [82,83,122].

Thus, γ-stimulation also has immunological effects. On the one hand, it reduces toxic inflammation, thereby preventing DNA damage and neuronal death. On the other hand, it activates the phagocytic activity of microglia, preventing them from returning to their original state for a prolonged period and helping to remove Aβ and tau protein. However, the extent to which these effects are observed may depend on the individual’s age and general health. For convenience, a summary table of all the data discussed in the article is provided below (Appendix A, Table A1).

## 6. Optimization of γ-Stimulation

Selection of individual parameters associated with biological age. The effectiveness of visual γ-stimulation is affected by: brightness intensity, color (wavelength), flicker frequency, time and duration of exposure [95,99,270,271,272,273]. Also, the optimal parameters of SMS for γ-synchronization in elderly people may differ from those in young people [88,274]. Age-related differences in γ-synchronization using light stimuli may be associated with age-related changes in the eyes and brain. With age, retinal illuminance becomes less effective due to a decrease in pupillary miosis and color discrimination. The decrease in illuminance associated with lens yellowing is not the same for all wavelengths, and most color defects in the elderly are of the blue-yellow type [275,276]. The central frequency of γ-rhythms gradually decreases with age [88].

Let us consider these parameters in turn. Optimal parameters of GENUS for γ-wave synchronization in humans may differ from those in mice. In humans, γ-waves synchronized by light flickering with a frequency ≤ 54 Hz propagate to the frontocentral region, while those synchronized by light flickering with a frequency > 54 Hz do not [277]. The spectral power of the involved γ-waves increased with increasing light intensity. The color (wavelength) of light can also influence γ-wave stimulation [95,278].

### 6.1. Visual γ-Stimulation in Young Adults

The optimal color, intensity and frequency of a flickering light stimulus for activating gamma waves differed between young adults (mean age 24 years) and more elderly persons [96]. When stimulating the brain with different colors (white, red, green and blue), red light was found to be the most effective in eliciting γ-waves, followed by white light. Light of higher intensity (400–700 cd/m^2^) elicited stronger γ-waves than light of lower intensity (10–100 cd/m^2^). The γ-waves were stronger and extended beyond the visual cortex when light flickered at a frequency of 34–38 Hz than when it flickered at a frequency of 40–50 Hz. Light with an intensity of 700 cd/m^2^ caused more severe side effects (eye fatigue, eye pain and glare) than light of other intensities. White light with a luminance of 400 cd/m^2^, flickering at a frequency of 34–38 Hz proved optimal for activating intrinsic γ-rhythms in young people.

The authors suggest that, although 40 Hz stimulation has been used in studies on AD mice [21,42,43], future human clinical studies should consider using 34–38 Hz stimulation instead, since 30 Hz light flickering has also been shown to prevent neurodegeneration of pyramidal neurons in the hippocampus of ischemic mouse models [47].

### 6.2. Visual γ-Stimulation in Older Adults

The optimal parameters of visual stimulation for synchronization with gamma rhythms in elderly people with normal cognitive function were studied at different brightness levels, colors and flicker frequencies [60].

The 700 cd/m^2^ flicker induced a stronger and more widespread γ-rhythm than the 400 cd/m^2^ flicker, and had no significant negative effect [60]. Furthermore, flicker at 32 Hz or 34 Hz induced a stronger and more widespread γ-rhythm than flicker at other higher frequencies. However, white and red flicker induced γ-rhythm equally in the brains of older adults.

Thus, older adults may tolerate brighter light better than younger adults, possibly due to age-related increases in miosis and lenticular cell senescence. For this reason, older adults may require brighter light stimulation to synchronize with the gamma rhythm, similar to younger adults. In patients with Alzheimer’s disease (AD), adverse effects from exposure to very low levels of light (VLS) of approximately 700 candela per square meter (cd/m^2^) were also shown to be minor [191].

The flicker frequency at which the strongest and most widespread γ-rhythm synchronized was 32 or 34 Hz in older adults and 36 or 38 Hz in young adults in a previous study [96]. The γ-rhythms synchronized to 32 or 34 Hz were approximately 1.2 times stronger at the Pz (center of head) site and 1.4 times stronger at the Fz (frontal) site than those synchronized to 40 Hz in older adults. Furthermore, γ-rhythms synchronized to 32 and 34 Hz were approximately 1.3 times stronger, covering approximately twice as many nodes as those synchronized to 40 Hz. In young adults, stimulation at 38 Hz synchronized γ-rhythms approximately 1.2 times more strongly in both Pz and Fz and covered approximately twice as many nodes, with a strength 2.8 times higher than at 40 Hz. These results indicate that the optimal flicker frequency for γ-rhythm synchronization in humans may differ significantly from that in mice and may also decrease with age.

### 6.3. The Problem of Stroboscopic Flicker and Its Solution

Using stroboscopic flicker, also known as temporally modulated light, for medical purposes is associated with certain difficulties due to its flickering nature. In human safety and feasibility trials, 28% of participants discontinued the therapy, which may indicate an issue with stroboscopic stimulation [279]. However, one study shows the safety of audiovisual gamma stimulation [191]. The side effects of stroboscopic light decrease as the temporal modulation frequency increases, until flickering is no longer perceived. The critical flicker fusion frequency is the frequency at which temporally modulated light ceases to be perceived as flickering [280]. It depends on the color and depth of the flicker modulation, as well as on the characteristics of the observer. For strobe lights with luminance of 100–500 lux, which were used in studies [24,30,37,58,59,62], the flicker fusion frequency is in the range of 55–70 Hz [280], that is, at least 15 Hz higher than the stimulation frequency of 40 Hz, making the flicker very noticeable to observers.

Heterochromatic (or simply chromatic) flicker replaces the “off” phase of stroboscopic flicker with a phase consisting of light of a different color, so that the light alternates between two different colors. Replacing stroboscopic flicker with chromatic flicker can reduce the critical flicker fusion frequency to 15–30 Hz (depending on luminance), improving neural oscillatory response and reducing potential side effects. This makes chromatic flicker a possible candidate for a 40 Hz stimulation paradigm in humans.

Spectral adjacent color pairs appear less flickering to the perceiver, so these results encourage further exploration of the limits of phase similarity that still elicit a 40 Hz response. Specifically, pairing a color at one extreme of the visible spectrum with another similar color may provide the best compromise between the perception of flicker and the magnitude of the steady-state visual evoked potentials (SVEP).

The study presented a new type of heterochromatic flicker based on spectral combinations of blue, cyan, green, light green, amber, and red colors (BCGLAR color space) [281]. The combination of amber and red flicker induced the highest SVEP, and combinations including blue and/or red colors consistently induced higher SVEP than combinations with only mid-spectrum colors.

The creation of an invisible spectral flicker (ISF) that induces neural synchronization at a frequency of 40 Hz and a spatial distribution similar to a 40 Hz stroboscopic light [282]. The type of ISF used in this study involves alternating two spectrally distinct light compositions, making the flickering imperceptible to the observer but creating the sensation of white light, similar to continuous light. The subtle flickering characteristic of ISF makes it similar to light therapy. It has already been recognized as an effective treatment for various mental disorders and dementia [283,284].

It must be acknowledged that the stroboscopic signal induced a significantly greater SVEP than ISF (approximately 4 times [282]); nevertheless, the resulting method allows for more comfortable stimulation and opens up the possibility of conducting future randomized, placebo-controlled clinical trials with an acceptable double-blind method due to the virtually imperceptible flicker, which is expected to significantly reduce discomfort compared to interventions based on stroboscopic flicker.

### 6.4. Increasing the Exposure Time by Stimulation During Sleep

As shown above, the results of γ-stimulation and the removal of neurotoxic molecules are inconclusive. The authors suggested that the limited duration of stimulation was a limiting factor [285]. They tested the feasibility of applying visual stimulation at a frequency of 40 Hz during sleep. This stimulation effectively induced γ-activity in neurons at the stimulation frequency without deteriorating sleep quality, thus confirming the feasibility of this approach. Furthermore, this approach could utilize glymphatic system activation to remove neurotoxic Aβ molecules from the brain. This neural mechanism can be exploited through stimulation at a frequency of 40 Hz during sleep [286,287]. Therefore, in addition to improving ease of use, stimulation at 40 Hz during sleep is likely to be more effective.

The well-established connection between the glymphatic system, dementia and sleep has already resulted in some fascinating developments in photobiomodulation therapy [288,289,290,291]. Photobiomodulation involves applying near-infrared light to specific areas of the brain through the scalp. This technique can be used to target several pathophysiological aspects in patients with AD, including the blood–brain barrier and glymphatic flow. It is also monitored by EEG [292]. Enhanced clearance of Aβ molecules is one of the main proposed mechanisms of this therapeutic approach, which is why it fits so well with sleep [290,291]. Early studies suggest that photobiomodulation can improve memory function and sleep quality in people with cognitive impairment [273,290,291]. Despite these promising results, the approach is still in its infancy and faces difficulties associated with light passing through the thick bone of the skull [293,294,295]. By contrast, exposure to red light through much thinner closed eyelids also stimulates the cerebral cortex, albeit via sensory pathways. This can be achieved while synchronizing oscillations at a frequency of 40 Hz, which may also promote glymphatic clearance [95,295,296]. At a frequency of 40 Hz, auditory evoked potentials were recorded during deep sleep stages [297,298]. Temporal lobe stimulation at 40 Hz prior to sleep has been shown to improve sleep quality in patients with insomnia. Taken together, the above studies clearly indicate the usefulness of using 40 Hz red light during sleep [299,300].

### 6.5. Cognitive Task During Stimulation

It was found that including a cognitive task involving attention and counting in both visual and auditory γ-stimulation sessions (sound anomaly detection) has a positive effect on the strength and duration of the γ-rhythm, promoting the spread of γ-oscillations to additional brain areas such as the frontal lobes and the hippocampus (which are not involved when participants are not required to perform a cognitive task) [123,124]. The latter is of particular interest since the hippocampus is considered a key target for AD treatment. This distinguishes γ-activity from activity in the β-range, which decreases with an increase in cognitive load [301]. The cognitive load increases both the power and coherence of γ-oscillations in the left frontotemporal and prefrontal sensor ranges during a syllable memorization task. The cortico-cortical connection between these structures increases [127]. Patterns of synchronization and cortico-cortical interactions were identified during the task of identifying sound inconsistencies, as well as visual and complex audio-visual inconsistencies [125]. Furthermore, the topography of gamma activity in the magnetoencephalogram during cognitive load largely coincided with the results of brain hemodynamics studies [126]. This indicates the significant benefit of cognitive tasks involving external γ-stimulation. Above, we examined the acetylcholine mechanism by which cognitive tasks influence γ-stimulation in more detail.

### 6.6. Motor (Behavioral) Activity During Stimulation

Recent studies in mice have shown that physical exercise combined with γ-therapy for four weeks reduces levels of amyloid and tau proteins to a greater extent than visual stimulation or exercise alone. This improves spatial learning, working memory and long-term memory. Furthermore, combining physical exercise with flickering gamma wave light improves calcium homeostasis, reduces reactive oxygen species levels and enhances cognitive abilities, mitochondrial function and neuroplasticity in mice [126,302]. Therefore, combining γ-stimulation with physical activity may be an effective way to enhance the desired effects.

### 6.7. Sound Wave Characteristics During Auditory Stimulation

As mentioned previously, a key issue is that prolonged and frequent image flickering can be hazardous to health, particularly for the eyes. In addition to the potential health risks, light flickering at 40 Hz can cause artifacts. Interestingly, human EEG results showed that the neural response evoked by synchronization with a single 40 Hz sound stimulus is weaker than the response to a visual stimulus [192]. This suggests that artifacts resulting from eye movements, such as regular blinking, are difficult to eliminate during the experiment, meaning that the associated EEG signals may be stronger than the brain signals of interest. Furthermore, keeping the eyes open may result in increased alpha activity in the occipital lobe, as well as changes in the topology and activity of different frequency bands compared to when the eyes are closed [303]. A possible solution that would make participants more comfortable and reduce artifacts would be to ask them to close their eyes. This is feasible when visual perception is not required. Eliminating medical and technical limitations could boost the popularity of auditory stimulation as a preventive measure and treatment for pathological changes in the brain, particularly given the evidence of successful γ-frequency oscillation targeting and the non-invasive nature of this method.

The effects of several new experimental conditions (sine or square wave sounds; eyes open and eyes closed conditions) using auditory stimulation to determine which condition could elicit a stronger neural response to a frequency of 40 Hz is well discribed [304]. The power of the 40 Hz signal was highest in the frontal lobes of the brain and when the eyes were closed during playback of a 40 Hz sine wave sound, compared to the other experimental conditions. Additionally, suppression of the α rhythm was observed. Furthermore, stronger suppression of the α-rhythms was observed in the prefrontal region when square signals of 40 Hz were used.

The neural response to a 40 Hz frequency evoked by a sine wave is stronger than that evoked by a square wave. This is probably because a sine wave is a simpler oscillation than a square wave, which contains more oscillatory components. The brain may find it easier to synchronize with the properties of a sine wave.

Results obtained using auditory stimulation at 40 Hz revealed increased neural activity in the parietal and prefrontal regions, where the default mode network (DMN) is located [193,194]. This suggests that the neural response during 40 Hz synchronization may be related to higher-level brain functions, such as learning, attention or memory.

### 6.8. The Influence of Glucose Metabolism on the γ-Rhythm

One manifestation of Alzheimer’s disease (AD) is disturbances in glucose metabolism, which manifest as increased oxidative stress before amyloid plaques appear [305,306]. Glucose levels are directly related to gamma oscillations. As maintaining γ-rhythms is an energy-intensive process that depends on a high rate of oxidative phosphorylation in neuronal mitochondria, which is limited by glucose availability, the authors hypothesize that insulin may be involved in the metabolic control of γ-oscillations [230]. PV+ interneurons selectively express the insulin-dependent glucose transporter GLUT4, which can provide an additional influx of glucose under conditions close to those that are limiting, such as during high-frequency γ-oscillations. Therefore, the effect of gamma stimulation may depend on glucose metabolism and the level of insulin resistance. To achieve a high-quality effect, attention must be paid to the presence of metabolic disorders in patients.

## 7. Clinical Trials

The large amount of data on the effectiveness of γ-stimulation has undoubtedly attracted the interest of the medical community. One consequence of this interest is attempts to create new medical devices and conduct clinical trials.

As of November 2025, GENUS devices have most frequently been tested or are planned to be tested in the treatment of Alzheimer’s disease and Parkinson’s disease, according to the database https://clinicaltrials.gov/ (accession date 17 November 2025).

A phase I/II randomized, controlled, single-blind multi-center clinical trial of the GammaSense Stimulation device medical device is currently underway. This device is intended for use in the treatment of prodromal AD, sever AD or Mild Cognitive Impairment (MCI) in men and women over the age of 55 years. During the trials, participants underwent daily gamma stimulation for 1 h/day for 6 months. The results of the study have already been obtained and published, according to which gamma stimulation partially relieves the symptoms of AD, improves the results of the Mental State Examination and ADSC-ADL (Instrumental, basic, and total) tests, and improves sleep quality [63,307]. However, in addition to the therapeutic effects, side effects of γ-stimulation were also found. The most common adverse effects were ear and labyrinth disorders, tinnitus, and headache (15–21%). Musculoskeletal and connective tissue, skin and subcutaneous tissue disorders occurred in 8–13% of participants, which is less frequent but higher than in the placebo group [307]. Clinical trial data indicate the need to modify the stimulation protocol to minimize the undesirable side effects of gamma stimulation.

A clinical trial of the GENUS device (NCT05655195) is also planned for individuals of both sexes diagnosed with AD aged 65–100 years. Participants will undergo daily combined (light and sound) stimulation at 40 Hz for 1 h per day, every day for 6 months, followed by analysis of changes in functional brain connectivity, cognitive functions, sleep/wake patterns and molecular biomarkers of AD. In addition to potential therapeutic effects, potential side effects (headache, changes in emotional status, etc.) and other risks associated with gamma stimulation will be evaluated.

In addition to AD, participants are being recruited for clinical trials of the use of 40 Hz frequency light, sound, and tactile stimulation in the treatment of Parkinson’s disease (restoration of motor functions) NCT05268887. Clinical trials will be conducted on individuals aged 45–90 with a confirmed diagnosis. The study is based on the results of randomized trials in patients with Parkinson’s disease undergoing deep brain stimulation at frequencies of 130–140 Hz [308,309,310]. However, in this case, it is difficult to speak of classical γ-stimulation, since both the central frequency (133–140 Hz as opposed to 40 Hz) and the method of stimulation (direct electrical stimulation of deep brain centers using an implantable device) differ significantly. In this case, there are significant risks of side effects from invasive intervention, and the highest qualifications of the operating neurosurgeon are required. Therefore, the planned medical trial will evaluate the efficacy and safety of γ-stimulation in the treatment of Parkinson’s disease.

The initial success of gamma stimulation as a potential therapeutic approach for AD and Parkinson’s disease [62] suggests its possible effectiveness in the treatment of other disorders. In particular, participants of both sexes aged 18–75 with depressive disorders are being recruited to test the therapeutic potential and safety of GENUS (NCT05680220). Unfortunately, the publicly available documentation does not allow us to evaluate all the characteristics of the stimulating effect: the wavelength of the stimulating light, the frequency and volume of the sound, and the characteristics of the tactile stimuli (if any). Therefore, the use of these clinical studies at this stage of our meta-analysis is difficult. We are confident that upon completion of the clinical studies, their results will be published and available to supplement the overall picture.

## 8. Limitations and Prospects

We attempted to systematize the literature on the effects of γ-stimulation and identify the relationship between its effectiveness and various factors such as the nature and frequency of the stimulus, the species and age of the organism, and the presence of pathology. We confirmed and expanded upon existing data regarding changes in the range of effective stimulation frequencies with age. We also identified differences in interspecies responses to γ-stimulation. We hope that our results will help future researchers to design more effective and efficient experiments on this topic.

However, like any other work in this area, this work has a number of limitations. Firstly, not all characteristics of periodic stimuli could be analyzed. In particular, there are no unified units of measurement for LED luminance in the case of a visual signal. Different studies use different characteristics and units of measurement for light sources, such as luminous flux (lm), illuminance (lx), luminous intensity (cd), luminous intensity per area (cd/cm^2^) or power (mW/cm^2^). Some units can be relatively accurately converted to each other (e.g., lx and lm, or lx and cd/cm^2^), if the setup geometry is described in detail. However, the data is often insufficient for accurate calculations [39,54,96,311]. Some pairs of units cannot be converted to each other in principle based on published data (e.g., mW/cm^2^ and cd/cm^2^). Furthermore, even if we could accurately estimate the luminous flux, luminance or power of a light source, we would still be unable to estimate the proportion of light reaching the eye in each specific case. If necessary, a more in-depth study could be conducted using additional sources, standardized units of measurement for light source characteristics, and other exposure conditions. However, this was beyond the scope of this review.

In the case of sound stimulation, data is often given either on the sound level (dB), but frequency or power stay unclear [303].

Secondly, several interesting characteristics and methodological requirements were excluded from the analysis due to insufficient data. These included whether the eyes were open or closed, the magnitude of the contrast (Michelson contrast), the change in the angle of inclination of the image when the visual stimulus was presented, and the presence of a tactile stimulus [94,95,117,138]. We admit that each of the presented additional conditions may be important for the realization of the effects of γ-stimulation. However, statistical verification of this requires either an expansion of the analyzed literature base or additional experimental work in this area.

Thirdly, we employed relatively straightforward and speedy methods to evaluate the quantitative relationships between the effectiveness of γ-stimulation and its frequency, type, duration and the age of the subjects. The use of more sensitive multivariate approaches, such as principal component analysis and multivariate correlation, as well as AI for the automated analysis of primary data, may allow us to identify additional patterns and refine existing relationships.

For future research, it is important to clarify the relationship between the effectiveness of γ-stimulation in young (under 20 years) and middle-aged (30–50 years) individuals, as there are clearly gaps in this area. As a significant proportion of research into the potential therapeutic applications of γ-stimulation is conducted in mice, it is necessary to search for new, more valid models, particularly for Alzheimer’s disease (AD). Furthermore, the reproducibility of results between mice and humans must be carefully compared. In human studies, it is crucial that electrophysiological results are validated by behavioral and/or cognitive tests. We hope that the proportion of such comprehensive studies will increase in the future.

Medical history and the use of certain medications can have a significant impact on the effects of γ-stimulation in humans. We believe that analyzing large samples of patients and healthy volunteers, taking their medical histories into account, will significantly improve our understanding of how stimulus characteristics affect γ-stimulation.

## 9. Conclusions

The γ-oscillations play a crucial role in how the brain perceives, processes and stores information. Stimulation with synchronized light and sound (or other methods) at a frequency of 40 Hz effectively induces corresponding brain activity at the same frequency. Overall, stimulation of the brain at 40 Hz is associated with reduced neuroinflammation, enhanced synaptic transmission, and increased expression of genes associated with synaptic plasticity. PV+ and SST+ interneurons are sensitive to this stimulation. This results in increased coherence and activation of neural networks, particularly DMN, executive network and salience network, leading to improved cognitive function. Exposure to 40 Hz also increases cytokine production and phagocytic activity in microglia, normalizes circadian rhythms and increases the rate of glymphatic clearance of Aβ. Collectively, these effects lead to a reduction in amyloid plaque formation.

Human studies do not always yield definitive results. There is reason to believe that γ-stimulation requires careful selection of parameters, taking into account the central γ-frequency, hue, brightness and exposure duration. This automatically takes into account age-related changes and other physiological characteristics of the individual, such as the level of GABA production, as well as the possible contribution of selective insulin resistance of PV+ interneurons to the progression of CNS diseases associated with cognitive impairment.

To enhance the effects of γ-stimulation, such as broader coverage and stimulation of deep brain regions, cognitive tasks, physical activity and a combination of visual and auditory stimulation must be used. Furthermore, gentler stimulation methods must be found to avoid unwanted reactions to strobe light. The approach of stimulation during sleep is very interesting.

Special attention should be paid to the use of medications during γ-therapy. Some substances can reduce the effect of gamma synchronization, in particular the calcium channel blocker felodipine. Conversely, NMDA receptor antagonists (of which there are many drugs) can enhance the power of abnormal γ-oscillations. The expression of chloride and sodium ion channels, and therefore the substances that block them, also influences γ-synchronization. Furthermore, glucose metabolism and insulin resistance should be considered, as these can affect the outcome of γ-stimulation.

The γ-stimulation effects have been found to vary depending on species, age, frequency and stimulus type. Visual stimulation protocols using white light are the most effective in humans. The range of effective central frequencies narrows from 20–60 Hz to 30–40 Hz as people age. While some studies have observed a decrease in the central frequency, others have shown an increase above 40 Hz for individuals aged 30–50. These findings must be taken into account when selecting an individual stimulation regimen.

AD patients and mouse models respond differently to γ-stimulation, so special attention should be paid to the selection of study subjects when assessing therapeutic potential. Additionally, cognitive and behavioral responses are less sensitive to gamma stimulation than biochemical and EEG responses. Therefore, it is advisable to supplement EEG data with behavioral studies to avoid false-positive results.

Our results may be useful in deepening the understanding of the mechanisms of gamma stimulation, when planning future experiments and in the search for potential therapeutic regimens of γ-stimulation.

## Data Availability

The data presented in this study are available in Appendix A, Table A1.

## Abbreviations

The following abbreviations are used in this manuscript:

3D	Three-dimensional
ACh	Acetylcholine
AD	Alzheimer’s disease
ANOVA	Analysis of variance
CNS	Central nervous system
CSF1	Colony-stimulating factor 1
DMN	default mode network
EEG	Electroencephalogram
GABA	γ-aminobutyric acid
GENUS	Gamma ENtrainment Using Sensory stimuli
IFN-γ	Interferon gamma
IL	Interleukin
ING	Purely inhibitory populations of neurons
iNOS	inducible nitric oxide synthase
IR	Infrared
ISF	Invisible spectral flicker
JNK	c-Jun N-terminal kinase
LED	Light-emitting diode
mAChRs	Muscarinic ACh receptors
MAPK	Mitogen-activated protein kinase
M-CSF	Macrophage colony-stimulating factor
MIP-1β	Macrophage inflammatory protein-1β
NF-κB	Nuclear factor kappa-light-chain-enhancer of activated B
NMDA	N-Methyl-D-aspartic acid
PING	Excitatory–inhibitory networks of neurons
PKR	Protein kinase R
PLX3397	Inhibitor of the colony-stimulating factor 1
PV	Parvalbumin
SST	Somatostatin
SVEP	Steady-state visual evoked potentials
TLR	Toll-like receptors
TNF-α	Tumor necrosis factor-alpha
UV	Ultraviolet
VIP	vasoactive intestinal peptide
Ym1	Heparin-binding lectin

## Appendix A

**Table A1:** The examples of γ-stimulation of the brain, types and effects.

#	Organism	Age, Years	Pathology	Type of Impact	Type of Exhibition	Duration, s	Additional Conditions	Additional Conditions Characteristics	Analyzed Parameter	Effect Module %	Ref.
1	Human	35	Healthy	Audio	Binaural	120	Eyes	Open	Central–frontal region eeg 25–50 hz oscillations	25	[304]
	Human	35	Healthy	Audio	Binaural	120	Eyes	Closed	Central–frontal region eeg 25–50 hz oscillations	40	
	Human	35	Healthy	Audio	Binaural	120	Eyes	Open	Central–frontal region eeg 25–50 hz oscillations	26	
	Human	35	Healthy	Audio	Binaural	120	Eyes	Closed	Central–frontal region eeg 25–50 hz oscillations	25	
2	Human	20	Healthy	Audio	Binaural	120	Eyes	Open	Alpha rhythm	53	[303]
	Human	20	Healthy	Audio	Binaural	120	Eyes	Closed	Alpha rhythm	29	
	Human	20	Healthy	Audio	Binaural	1920	Eyes	Open	Alpha rhythm	24	
	Human	20	Healthy	Audio	Binaural	1920	Eyes	Closed	Alpha rhythm	53	
	Human	20	Healthy	Audio	Binaural	120	Eyes	Open	Deplta rhythm	23	
	Human	20	Healthy	Audio	Binaural	120	Eyes	Closed	Deplta rhythm	5	
	Human	20	Healthy	Audio	Binaural	1920	Eyes	Open	Deplta rhythm	32	
	Human	20	Healthy	Audio	Binaural	1920	Eyes	Closed	Deplta rhythm	14	
	Human	20	Healthy	Audio	Binaural	120	Eyes	Open	Theta rhythm	22	
	Human	20	Healthy	Audio	Binaural	120	Eyes	Closed	Theta rhythm	11	
	Human	20	Healthy	Audio	Binaural	1920	Eyes	Open	Theta rhythm	33	
	Human	20	Healthy	Audio	Binaural	1920	Eyes	Closed	Theta rhythm	22	
3	Human	9	Insomnia	Light	Visually	1800	Wavelength, nm	750	Sol duration	81	[299]
	Human	9	Insomnia	Light	Visually	1800	Wavelength, nm	750	Tst duration	12	
	Human	9	Insomnia	Light	Visually	1800	Wavelength, nm	750	Se duration	15	
	Human	9	Insomnia	Light	Visually	1800	Wavelength, nm	750	Rem amount	6	
4	Human	60	BA	Light	Visually	“-”	Wavelength, nm	750	N-back test accuracy	11	[293]
	Human	60	BA	Light	Visually	“-”	Wavelength, nm	750	Reaction time in a 1-back test	8	
5	Human	22	Healthy	Light	Visually	350	Wavelength, nm	810	Score of the computerized visual memory test	29	[273]
	Human	22	Healthy	Light	Visually	350	Wavelength, nm	810	Longest correct span of the computerized visual memory test	16	
	Human	22	Healthy	Light	Visually	350	Wavelength, nm	810	Oxi-hb changedduring early phase of test	83	
	Human	22	Healthy	Light	Visually	350	Wavelength, nm	810	Oxi-hb changedduring late phase of test	38	
6	Human	70	BA	Light	Visually	126,000	Wavelength, nm	810	Mini-mental state exam results	15	[312]
	Human	70	BA	Light	Visually	126,000	Wavelength, nm	810	Alzheimer’s disease assessment scale-cognitive subscale scores	19	
	Human	70	BA	Light	Visually	63,000	Wavelength, nm	810	Mini-mental state exam results	14	
	Human	70	BA	Light	Visually	63,000	Wavelength, nm	810	Alzheimer’s disease assessment scale-cognitive subscale scores	19	
7	Human	80	BA	Light	Visually	21,600	Wavelength, nm	810	Alzheimer’s disease assessment scale-cognitive scores	5	[313]
	Human	80	BA	Light	Visually	21,600	Wavelength, nm	810	Alzheimer’s disease assessment scale-cognitive scores	14	
	Human	80	BA	Light	Visually	21,600	Wavelength, nm	810	Neuropsychiatric inventory scopes	35	
	Human	80	BA	Light	Visually	21,600	Wavelength, nm	810	Neuropsychiatric inventory scopes	61	
	Human	80	BA	Light	Visually	21,600	Wavelength, nm	810	Total perfusion	37	
	Human	80	BA	Light	Visually	21,600	Wavelength, nm	810	Superior frontal perfusion	3	
	Human	80	BA	Light	Visually	21,600	Wavelength, nm	810	Superior parietal perfusion	88	
	Human	80	BA	Light	Visually	21,600	Wavelength, nm	810	Supramarginal perfusion	28	
	Human	80	BA	Light	Visually	21,600	Wavelength, nm	810	Connectivy between posterior cingulate cortex and left lateral parietal	233	
	Human	80	BA	Light	Visually	21,600	Wavelength, nm	810	Connectivy between posterior cingulate cortex and right lateral parietal	181	
8	Human	65	Parkinson’s disease	Light	Visually	8568	Wavelength, nm	1080	Trail making a	63	[313]
	Human	65	Parkinson’s disease	Light	Visually	8568	Wavelength, nm	1080	Adas- cog ideational praxis	22	
	Human	65	Parkinson’s disease	Light	Visually	8568	Wavelength, nm	1080	Adas- cog boston naming	11	
	Human	65	Parkinson’s disease	Light	Visually	8568	Wavelength, nm	1080	Cortical perfusion	5	
9	Human	76	Dementia	Light	Visually	72,000	Wavelength, nm	700	Emotional state	71	[284]
	Human	76	Dementia	Light	Visually	72,000	Wavelength, nm	700	Psychiatric symptom	43	
	Human	76	Dementia	Light	Visually	144,000	Wavelength, nm	700	Psychiatric symptom	79	
	Human	76	Dementia	Light	Visually	72,000	Wavelength, nm	700	Sleep disturbances	73	
	Human	76	Dementia	Light	Visually	144,000	Wavelength, nm	700	Sleep disturbances	82	
	Human	76	Dementia	Light	Visually	72,000	Wavelength, nm	700	Orientation	54	
	Human	76	Dementia	Light	Visually	144,000	Wavelength, nm	700	Orientation	62	
10	Human	25	Healthy	Light	Visually	1800	Maximum wavelength, nm	630	Gamma-rhythm power	1200	[282]
	Human	25	Healthy	Light	Visually	1800	Maximum wavelength, nm	450	Gamma-rhythm power	350	
11	Human	28	Healthy	Light	Visually	300	Wavelength, nm	500	Gamma-rhythm power	300	[281]
	Human	28	Healthy	Light	Visually	300	Wavelength, nm	450	Gamma-rhythm power	600	
	Human	28	Healthy	Light	Visually	300	Wavelength, nm	550	Gamma-rhythm power	700	
12	Human	24.8	Healthy	Light	Visually	600	Wavelength, nm	555	Alpha rhythm power	200	[272]
	Human	24.8	Healthy	Light	Visually	600	Wavelength, nm	555	Alpha rhythm power	150	
13	Human	71.5	BA	Light	Visually	72,000	Wavelength, nm	555	Amyloid-*β* (a *β*) content	1	[270]
14	Human	25	Healthy	Light	Visually	300	Wavelength, nm	555	Gamma-rhythm power without cognitive task	900	[123]
	Human	25	Healthy	Light	Visually	300	Wavelength, nm	555	Gamma-rhythm power with cognitive task	700	
	Human	25	Healthy	Light	Visually	300	Wavelength, nm	555	Different in p300 amplitude	300	
	Human	25	Healthy	Light	Visually	300	Wavelength, nm	555	Different in p300 latency	4500	
15	Human	71	BA	Lighting and audio	Combination	16,470	Wavelength, nm	745	Gamma-rhythm power	1400	[192]
	Human	71	BA	Lighting and audio	Combination	16,470	Wavelength, nm	745	Ventricular volume change	60	
	Human	71	BA	Lighting and audio	Combination	16,470	Wavelength, nm	745	Hippocampal atrophy	55	
	Human	71	BA	Lighting and audio	Combination	16,470	Wavelength, nm	745	Pcc connectivity	125	
	Human	71	BA	Lighting and audio	Combination	16,470	Wavelength, nm	745	Mediam visual network connectivity	161	
	Human	71	BA	Lighting and audio	Combination	16,470	Wavelength, nm	745	Interdaily sleep stability	200	
	Human	71	BA	Lighting and audio	Combination	16,470	Wavelength, nm	745	Wake after sleep onset	1300	
	Human	71	BA	Lighting and audio	Combination	16,470	Wavelength, nm	745	Accuracy	580	
16	Human	72	BA	Lighting and audio	Combination	201,600	Wavelength, nm	555	Pcc-pcu functional connectivity	30	[191]
	Human	72	BA	Lighting and audio	Combination	201,600	Wavelength, nm	555	Tweak immune factor expression	10	
17	Human	11.5	Healthy	Image rotation	Visually	2100	Wavelength, nm	555	Gamma-rhythm power	1300	[138]
	Human	11.5	Healthy	Image rotation	Visually	2100	Wavelength, nm	555	Gamma-rhythm power	1000	
	Human	11.5	Healthy	Image rotation	Visually	2100	Wavelength, nm	555	Gamma-rhythm power	600	
	Human	11.5	Healthy	Image rotation	Visually	2100	Wavelength, nm	555	Gamma-rhythm power change in k29	250	
	Human	11.5	Healthy	Image rotation	Visually	2100	Wavelength, nm	555	Gamma-rhythm power change in k29	220	
	Human	11.5	Healthy	Image rotation	Visually	2100	Wavelength, nm	555	Gamma-rhythm power change in k29	120	
	Human	11.5	Healthy	Image rotation	Visually	2100	Wavelength, nm	555	Gamma-rhythm power change in k18	330	
	Human	11.5	Healthy	Image rotation	Visually	2100	Wavelength, nm	555	Gamma-rhythm power change in k18	290	
	Human	11.5	Healthy	Image rotation	Visually	2100	Wavelength, nm	555	Gamma-rhythm power change in k18	90	
	Human	11.5	Healthy	Image rotation	Visually	2100	Wavelength, nm	555	Gamma-rhythm power change in k19	360	
	Human	11.5	Healthy	Image rotation	Visually	2100	Wavelength, nm	555	Gamma-rhythm power change in k19	320	
	Human	11.5	Healthy	Image rotation	Visually	2100	Wavelength, nm	555	Gamma-rhythm power change in k19	205	
18	Human	27.5	Healthy	Light	Visually	600	Wavelength, nm	555	Gamma-rhythm power by fmri	14	[314]
	Human	27.5	Healthy	Light	Visually	600	Wavelength, nm	555	Gamma-rhythm power by fmri	38	
	Human	27.5	Healthy	Light	Visually	600	Wavelength, nm	555	Gamma-rhythm power by fmri	43	
	Human	27.5	Healthy	Light	Visually	600	Wavelength, nm	555	Gamma-rhythm power by fmri	46	
	Human	27.5	Healthy	Light	Visually	600	Wavelength, nm	555	Gamma-rhythm power by meg	29	
	Human	27.5	Healthy	Light	Visually	600	Wavelength, nm	555	Gamma-rhythm power by meg	6	
	Human	27.5	Healthy	Light	Visually	600	Wavelength, nm	555	Gamma-rhythm power by meg	40	
	Human	27.5	Healthy	Light	Visually	600	Wavelength, nm	555	Gamma-rhythm power by meg	43	
19	Human	33	Healthy	Light	Visually	10	Wavelength, nm	555	Gamma-rhythm power by meg in v1	100	[117]
	Human	33	Healthy	Light	Visually	10	Wavelength, nm	555	Gamma-rhythm power by meg in v1 lfp	560	
	Human	33	Healthy	Light	Visually	10	Wavelength, nm	555	Meg in v1 lfp signle spike density	567	
	Human	33	Healthy	Light	Visually	10	Michelson contrast, %	20	Gamma-rhythm power by meg	20	
	Human	33	Healthy	Light	Visually	10	Michelson contrast, %	36	Gamma-rhythm power by meg	200	
	Human	33	Healthy	Light	Visually	10	Michelson contrast, %	48	Gamma-rhythm power by meg	300	
	Human	33	Healthy	Light	Visually	10	Michelson contrast, %	66	Gamma-rhythm power by meg	340	
	Human	33	Healthy	Light	Visually	10	Michelson contrast, %	96	Gamma-rhythm power by meg	490	
20	Human	37.8	Healthy	Light	Visually	420	Wavelength, nm	370	Theta rhythm power	205	[104]
	Human	37.8	Healthy	Light	Visually	420	Wavelength, nm	555	Theta rhythm power	215	
	Human	37.8	Healthy	Light	Visually	420	Wavelength, nm	370	Visually evoked potential	215	
	Human	37.8	Healthy	Light	Visually	420	Wavelength, nm	370	Alpha-gamma coupling f5	1400	
	Human	37.8	Healthy	Light	Visually	420	Wavelength, nm	370	Alpha-gamma coupling c4	200	
21	Human	23.9	Healthy	Light	Visually	5	Wavelength, nm	390	Mean ssvep power	15	[103]
	Human	23.9	Healthy	Light	Visually	5	Wavelength, nm	430	Mean ssvep power	19	
	Human	23.9	Healthy	Light	Visually	5	Wavelength, nm	390	Mean msi power	33	
	Human	23.9	Healthy	Light	Visually	5	Wavelength, nm	430	Mean msi power	42	
	Human	23.9	Healthy	Light	Visually	5	Wavelength, nm	430	Mean of cca coeddicient	25	
22	Human	28.5	Healthy	Light	Visually	15	Wavelength, nm	440	Maximum gamma rhythm peaks values	63	[99]
	Human	28.5	Healthy	Light	Visually	15	Wavelength, nm	600	Maximum gamma rhythm peaks values	53	
	Human	28.5	Healthy	Light	Visually	15	Wavelength, nm	600	Maximum gamma rhythm peaks values	133	
	Human	28.5	Healthy	Light	Visually	15	Wavelength, nm	600	Maximum gamma rhythm peaks values	150	
	Human	28.5	Healthy	Light	Visually	15	Wavelength, nm	600	Maximum gamma rhythm peaks values	113	
	Human	28.5	Healthy	Light	Visually	15	Wavelength, nm	560	Maximum gamma rhythm peaks values	67	
23	Human	69.9	Healthy	Light	Visually	695	Wavelength, nm	560	Event-related synchronization in pz region	800	[72]
	Human	69.9	Healthy	Light	Visually	695	Wavelength, nm	560	Event-related synchronization in pz region	850	
	Human	69.9	Healthy	Light	Visually	695	Wavelength, nm	560	Event-related synchronization in pz region	830	
	Human	69.9	Healthy	Light	Visually	695	Wavelength, nm	560	Event-related synchronization in pz region	820	
	Human	69.9	Healthy	Light	Visually	695	Wavelength, nm	560	Event-related synchronization in pz region	790	
	Human	69.9	Healthy	Light	Visually	695	Wavelength, nm	560	Event-related synchronization in pz region	780	
	Human	69.9	Healthy	Light	Visually	695	Wavelength, nm	560	Event-related synchronization in fz region	430	
	Human	69.9	Healthy	Light	Visually	695	Wavelength, nm	560	Event-related synchronization in fz region	420	
	Human	69.9	Healthy	Light	Visually	695	Wavelength, nm	560	Event-related synchronization in fz region	400	
	Human	69.9	Healthy	Light	Visually	695	Wavelength, nm	560	Event-related synchronization in fz region	380	
	Human	69.9	Healthy	Light	Visually	695	Wavelength, nm	560	Event-related synchronization in fz region	370	
	Human	69.9	Healthy	Light	Visually	695	Wavelength, nm	560	Strength of connectivity	280	
	Human	69.9	Healthy	Light	Visually	695	Wavelength, nm	560	Strength of connectivity	320	
	Human	69.9	Healthy	Light	Visually	695	Wavelength, nm	560	Strength of connectivity	400	
	Human	69.9	Healthy	Light	Visually	695	Wavelength, nm	560	Strength of connectivity	370	
	Human	69.9	Healthy	Light	Visually	695	Wavelength, nm	560	Strength of connectivity	330	
	Human	69.9	Healthy	Light	Visually	695	Wavelength, nm	560	Strength of connectivity	240	
24	Human	21.4	Healthy	Light	Visually	695	Wavelength, nm	700	Event-related synchronization in pz region	230	[96]
	Human	21.4	Healthy	Light	Visually	695	Wavelength, nm	530	Event-related synchronization in pz region	200	
	Human	21.4	Healthy	Light	Visually	695	Wavelength, nm	440	Event-related synchronization in pz region	125	
	Human	21.4	Healthy	Light	Visually	695	Wavelength, nm	610	Event-related synchronization in pz region	95	
	Human	21.4	Healthy	Light	Visually	695	Wavelength, nm	700	Event-related synchronization in fz region	50	
	Human	21.4	Healthy	Light	Visually	695	Wavelength, nm	530	Event-related synchronization in fz region	40	
	Human	21.4	Healthy	Light	Visually	695	Wavelength, nm	440	Event-related synchronization in fz region	20	
	Human	21.4	Healthy	Light	Visually	695	Wavelength, nm	610	Event-related synchronization in pz region	16	
	Human	21.4	Healthy	Light	Visually	695	Wavelength, nm	610	Event-related synchronization in pz region	400	
	Human	21.4	Healthy	Light	Visually	695	Wavelength, nm	610	Event-related synchronization in pz region	570	
	Human	21.4	Healthy	Light	Visually	695	Wavelength, nm	610	Event-related synchronization in pz region	590	
	Human	21.4	Healthy	Light	Visually	695	Wavelength, nm	610	Event-related synchronization in fz region	140	
	Human	21.4	Healthy	Light	Visually	695	Wavelength, nm	610	Event-related synchronization in fz region	210	
	Human	21.4	Healthy	Light	Visually	695	Wavelength, nm	610	Event-related synchronization in pz region	230	
	Human	21.4	Healthy	Light	Visually	695	Wavelength, nm	610	Event-related synchronization in pz region	400	
	Human	21.4	Healthy	Light	Visually	695	Wavelength, nm	610	Event-related synchronization in pz region	450	
	Human	21.4	Healthy	Light	Visually	695	Wavelength, nm	610	Event-related synchronization in pz region	500	
	Human	21.4	Healthy	Light	Visually	695	Wavelength, nm	610	Event-related synchronization in pz region	530	
	Human	21.4	Healthy	Light	Visually	695	Wavelength, nm	610	Event-related synchronization in pz region	450	
	Human	21.4	Healthy	Light	Visually	695	Wavelength, nm	610	Event-related synchronization in pz region	380	
	Human	21.4	Healthy	Light	Visually	695	Wavelength, nm	610	Event-related synchronization in pz region	360	
	Human	21.4	Healthy	Light	Visually	695	Wavelength, nm	610	Event-related synchronization in pz region	360	
	Human	21.4	Healthy	Light	Visually	695	Wavelength, nm	610	Event-related synchronization in fz region	200	
	Human	21.4	Healthy	Light	Visually	695	Wavelength, nm	610	Event-related synchronization in fz region	190	
	Human	21.4	Healthy	Light	Visually	695	Wavelength, nm	610	Event-related synchronization in fz region	205	
	Human	21.4	Healthy	Light	Visually	695	Wavelength, nm	610	Event-related synchronization in fz region	185	
	Human	21.4	Healthy	Light	Visually	695	Wavelength, nm	610	Event-related synchronization in fz region	145	
	Human	21.4	Healthy	Light	Visually	695	Wavelength, nm	610	Event-related synchronization in fz region	120	
	Human	21.4	Healthy	Light	Visually	695	Wavelength, nm	610	Event-related synchronization in fz region	110	
	Human	21.4	Healthy	Light	Visually	695	Wavelength, nm	610	Event-related synchronization in fz region	111	
25	Human	20	Healthy	Light	Visually	30	Eyes	Open	Gamma-rhythm power	150	[95]
	Human	20	Healthy	Light	Visually	30	Eyes	Closed	Gamma-rhythm power	150	
	Human	20	Healthy	Light	Visually	30	Eyes	Closed	Alhpa-rhythm power	300	
	Human	20	Healthy	Light	Visually	30	Eyes	Open	Number of electrodes regisctrared gamma-rhythm enhacne	1300	
	Human	20	Healthy	Light	Visually	30	Eyes	Open	Number of electrodes regisctrared gamma-rhythm enhacne	1700	
	Human	20	Healthy	Light	Visually	30	Eyes	Open	Number of electrodes regisctrared gamma-rhythm enhacne	500	
	Human	20	Healthy	Light	Visually	30	Eyes	Open	Number of electrodes regisctrared gamma-rhythm enhacne	1500	
	Human	20	Healthy	Light	Visually	30	Eyes	Open	Number of electrodes regisctrared gamma-rhythm enhacne	300	
	Human	20	Healthy	Light	Visually	30	Eyes	Open	Number of electrodes regisctrared gamma-rhythm enhacne	200	
	Human	20	Healthy	Light	Visually	20	Eyes	Open	Number of electrodes regisctrared gamma-rhythm enhacne	2100	
	Human	20	Healthy	Light	Visually	10	Eyes	Open	Number of electrodes regisctrared gamma-rhythm enhacne	1900	
	Human	20	Healthy	Light	Visually	5	Eyes	Open	Number of electrodes regisctrared gamma-rhythm enhacne	1200	
	Human	20	Healthy	Light	Visually	2	Eyes	Open	Number of electrodes regisctrared gamma-rhythm enhacne	1000	
26	Human	25.3	Healthy	Lighting and audio	Visually	2	Tactile stimuli	Eat	Test response time	8	[94]
	Human	25.3	Healthy	Lighting and audio	Visually	2	Tactile stimuli	Eat	Imaginary gamma-rhythm cogerence	100	
27	Human	67.4	Parkinson’s disease	Magnetic	Cranial	1200	“-”	“-”	Fingers velocity	5	[315]
	Human	67.4	Parkinson’s disease	Magnetic	Cranial	1200	“-”	“-”	Fingers velocity	4	
	Human	67.4	Parkinson’s disease	Magnetic	Cranial	1200	“-”	“-”	Fingers move amplitude	7	
	Human	67.4	Parkinson’s disease	Magnetic	Cranial	1200	“-”	“-”	Fingers move amplitude	5	
	Human	67.4	Parkinson’s disease	Magnetic	Cranial	1200	“-”	“-”	Short-interval intracortical inhibition	33	
	Human	67.4	Parkinson’s disease	Magnetic	Cranial	1200	“-”	“-”	Short-interval intracortical inhibition	17	
	Human	67.4	Parkinson’s disease	Magnetic	Cranial	1200	“-”	“-”	Short-latency afferent inhibition	22	
	Human	67.4	Parkinson’s disease	Magnetic	Cranial	1200	“-”	“-”	Short-latency afferent inhibition	22	
28	Human	24.5	Healthy	Light	Visually	30	Wavelength, nm	555	First harmonic oscillation amplitude	1700	[54]
	Human	24.5	Healthy	Light	Visually	30	Wavelength, nm	555	Second harmonic oscillation amplitude	200	
	Human	24.5	Healthy	Light	Visually	30	Wavelength, nm	555	First harmonic oscillation amplitude	600	
	Human	24.5	Healthy	Light	Visually	30	Wavelength, nm	555	Second harmonic oscillation amplitude	200	
	Human	24.5	Healthy	Light	Visually	30	Wavelength, nm	555	First harmonic oscillation amplitude	500	
	Human	24.5	Healthy	Light	Visually	30	Wavelength, nm	555	Second harmonic oscillation amplitude	100	
	Human	24.5	Healthy	Light	Visually	30	Wavelength, nm	555	First harmonic oscillation amplitude	240	
	Human	24.5	Healthy	Light	Visually	30	Wavelength, nm	555	First harmonic oscillation amplitude	440	
	Human	24.5	Healthy	Light	Visually	30	Wavelength, nm	555	First harmonic oscillation amplitude	220	
	Human	24.5	Healthy	Light	Visually	30	Wavelength, nm	555	First harmonic oscillation amplitude	1100	
	Human	24.5	Healthy	Light	Visually	30	Wavelength, nm	555	Other harmonics oscillation amplitude	300	
	Human	24.5	Healthy	Light	Visually	30	Wavelength, nm	555	First harmonic oscillation amplitude	3100	
	Human	24.5	Healthy	Light	Visually	30	Wavelength, nm	555	Other harmonics oscillation amplitude	5900	
	Human	24.5	Healthy	Light	Visually	30	Wavelength, nm	555	Other harmonics oscillation amplitude	3900	
	Human	24.5	Healthy	Light	Visually	30	Wavelength, nm	555	Other harmonics oscillation amplitude	2900	
29	Human	20.6	Healthy	Audio	Binourally	300	“-”	“-”	Gamma rhythm power in f4	20	[64]
	Human	20.6	Healthy	Audio	Binourally	600	“-”	“-”	Gamma rhythm power in f4	12	
	Human	20.6	Healthy	Audio	Binourally	900	“-”	“-”	Gamma rhythm power in f4	36	
	Human	20.6	Healthy	Audio	Binourally	1200	“-”	“-”	Gamma rhythm power in f4	18	
	Human	20.6	Healthy	Audio	Binourally	1500	“-”	“-”	Gamma rhythm power in f4	2	
	Human	20.6	Healthy	Audio	Binourally	1800	“-”	“-”	Gamma rhythm power in f4	4	
	Human	20.6	Healthy	Audio	Binourally	300	“-”	“-”	Gamma rhythm power in fp2	32	
	Human	20.6	Healthy	Audio	Binourally	600	“-”	“-”	Gamma rhythm power in fp2	11	
	Human	20.6	Healthy	Audio	Binourally	900	“-”	“-”	Gamma rhythm power in fp2	23	
	Human	20.6	Healthy	Audio	Binourally	1200	“-”	“-”	Gamma rhythm power in fp2	18	
	Human	20.6	Healthy	Audio	Binourally	1500	“-”	“-”	Gamma rhythm power in fp2	5	
	Human	20.6	Healthy	Audio	Binourally	1800	“-”	“-”	Gamma rhythm power in fp2	7	
	Human	20.6	Healthy	Audio	Binourally	300	“-”	“-”	Beta rhythm power in f4	15	
	Human	20.6	Healthy	Audio	Binourally	600	“-”	“-”	Beta rhythm power in f4	35	
	Human	20.6	Healthy	Audio	Binourally	900	“-”	“-”	Beta rhythm power in f4	23	
	Human	20.6	Healthy	Audio	Binourally	1200	“-”	“-”	Beta rhythm power in f4	12	
	Human	20.6	Healthy	Audio	Binourally	1500	“-”	“-”	Beta rhythm power in f4	15	
	Human	20.6	Healthy	Audio	Binourally	1800	“-”	“-”	Beta rhythm power in f4	8	
	Human	20.6	Healthy	Audio	Binourally	300	“-”	“-”	Beta rhythm power in fp2	27	
	Human	20.6	Healthy	Audio	Binourally	600	“-”	“-”	Beta rhythm power in fp2	34	
	Human	20.6	Healthy	Audio	Binourally	900	“-”	“-”	Beta rhythm power in fp2	41	
	Human	20.6	Healthy	Audio	Binourally	1200	“-”	“-”	Beta rhythm power in fp2	32	
	Human	20.6	Healthy	Audio	Binourally	1500	“-”	“-”	Beta rhythm power in fp2	18	
	Human	20.6	Healthy	Audio	Binourally	1800	“-”	“-”	Beta rhythm power in fp2	15	
	Human	20.6	Healthy	Audio	Binourally	300	“-”	“-”	Alpha rhythm power in f4	42	
	Human	20.6	Healthy	Audio	Binourally	600	“-”	“-”	Alpha rhythm power in f4	39	
	Human	20.6	Healthy	Audio	Binourally	900	“-”	“-”	Alpha rhythm power in f4	39	
	Human	20.6	Healthy	Audio	Binourally	1200	“-”	“-”	Alpha rhythm power in f4	18	
	Human	20.6	Healthy	Audio	Binourally	1500	“-”	“-”	Alpha rhythm power in f4	11	
	Human	20.6	Healthy	Audio	Binourally	1800	“-”	“-”	Alpha rhythm power in f4	11	
	Human	20.6	Healthy	Audio	Binourally	300	“-”	“-”	Alpha rhythm power in fp2	30	
	Human	20.6	Healthy	Audio	Binourally	600	“-”	“-”	Alpha rhythm power in fp2	7	
	Human	20.6	Healthy	Audio	Binourally	900	“-”	“-”	Alpha rhythm power in fp2	11	
	Human	20.6	Healthy	Audio	Binourally	1200	“-”	“-”	Alpha rhythm power in fp2	17	
	Human	20.6	Healthy	Audio	Binourally	1500	“-”	“-”	Alpha rhythm power in fp2	3	
	Human	20.6	Healthy	Audio	Binourally	1800	“-”	“-”	Alpha rhythm power in fp2	3	
	Human	20.6	Healthy	Audio	Binourally	1800	“-”	“-”	Word recall test result	100	
	Human	20.6	Healthy	Audio	Binourally	1800	“-”	“-”	Brunel mood scale worried	51	
	Human	20.6	Healthy	Audio	Binourally	1800	“-”	“-”	Brunel mood scale nervous	44	
	Human	20.6	Healthy	Audio	Binourally	1800	“-”	“-”	Brunel mood scale annoyed	104	
	Human	20.6	Healthy	Audio	Binourally	1800	“-”	“-”	Brunel mood scale miserable	56	
	Human	20.6	Healthy	Audio	Binourally	1800	“-”	“-”	Brunel mood scale sleepy	25	
	Human	20.6	Healthy	Audio	Binourally	1800	“-”	“-”	Brunel mood energy scale	39	
	Human	20.6	Healthy	Audio	Binourally	1800	“-”	“-”	Brunel mood scale muddled	32	
30	Human	66.5	BA	Lighting and audio	Visually	648,000	“-”	“-”	Active periods during nighttime	10	[63]
	Human	66.5	BA	Lighting and audio	Visually	648,000	“-”	“-”	Normalized active periods during nighttime	4	
	Human	66.5	BA	Lighting and audio	Visually	648,000	“-”	“-”	Activities of daily living	133	
31	Mouse	0.5	BA	Light	Cranial	216,000	Training day	2	Escape latency	7	[316]
	Mouse	0.5	BA	Light	Cranial	216,000	Training day	3	Escape latency	38	
	Mouse	0.5	BA	Light	Cranial	216,000	Training day	4	Escape latency	42	
	Mouse	0.5	BA	Light	Cranial	216,000	Training day	5	Escape latency	64	
	Mouse	0.5	BA	Light	Cranial	216,000	Training day	5	Step-through latency time	67	
	Mouse	0.5	BA	Light	Cranial	216,000	Training day	5	Time in probe quadrant	75	
	Mouse	0.5	BA	Light	Cranial	216,000	Training day	5	P-act expression level	50	
	Mouse	0.5	BA	Light	Cranial	216,000	Training day	5	P-gsk3beta expression level	40	
	Mouse	0.5	BA	Light	Cranial	216,000	Training day	5	P-tay expression level	30	
	Mouse	0.5	BA	Light	Cranial	216,000	Training day	5	App expression level	47	
	Mouse	0.5	BA	Light	Cranial	216,000	Training day	5	Number of abeta-positive cells	57	
	Mouse	0.5	BA	Light	Cranial	216,000	Training day	5	Number of gfap-positive astrocytes in ca	19	
	Mouse	0.5	BA	Light	Cranial	216,000	Training day	5	Number of gfap-positive astrocytes in dentate gyrus	25	
	Mouse	0.5	BA	Light	Cranial	216,000	Training day	5	Number of iba-1-positive astrocytes in ca	25	
	Mouse	0.5	BA	Light	Cranial	216,000	Training day	5	Number of iba-1-positive astrocytes in dentate gyrus	33	
	Mouse	0.5	BA	Light	Cranial	216,000	Training day	5	Tnfapha secretion	28	
	Mouse	0.5	BA	Light	Cranial	216,000	Training day	5	Il-6 secretion	27	
	Mouse	0.5	BA	Light	Cranial	216,000	Training day	5	Mitochondrial Ca^2+^ retention capacity	70	
	Mouse	0.5	BA	Light	Cranial	216,000	Training day	5	Mitochondrial ptp inhibition	67	
	Mouse	0.5	BA	Light	Cranial	216,000	Training day	5	Mitochondrial H_2_O_2_ generation	12	
	Mouse	0.5	BA	Light	Cranial	216,000	Training day	5	Bax protein expression level	33	
	Mouse	0.5	BA	Light	Cranial	216,000	Training day	5	Cyt c protein expression level	27	
	Mouse	0.5	BA	Light	Cranial	216,000	Training day	5	Caspase-3 protein expression level	31	
	Mouse	0.5	BA	Light	Cranial	216,000	Training day	5	Bcl-2 protein expression level	25	
	Mouse	0.5	BA	Light	Cranial	216,000	Training day	5	Tunel-positive cells number in dentate gyrus	21	
	Mouse	0.5	BA	Light	Cranial	216,000	Training day	5	Dcx-positive cells number in dentate gyrus	38	
	Mouse	0.5	BA	Light	Cranial	216,000	Training day	5	Neun/brdu-positive cells number in dentate gyrus	90	
32	Mouse	0.5	Healthy	Light	Cranial	1800	Wavelength, nm	545	Intracellular adenosine concentration in v1	400	[299]
	Mouse	0.5	Healthy	Light	Cranial	1800	Wavelength, nm	545	Intracellular adenosine concentration in v2	900	
	Mouse	0.5	Healthy	Light	Cranial	1800	Wavelength, nm	545	Intracellular adenosine concentration in v3	1100	
	Mouse	0.5	Healthy	Light	Cranial	1800	Wavelength, nm	545	Caicium infux	100	
	Mouse	0.5	Healthy	Light	Cranial	1800	Wavelength, nm	545	Caicium infux	180	
	Mouse	0.5	Healthy	Light	Cranial	1800	Wavelength, nm	545	Local field potential oscillations	20	
	Mouse	0.5	Healthy	Light	Cranial	1800	Wavelength, nm	545	Local field potential oscillations	30	
	Mouse	0.5	Healthy	Light	Cranial	14,400	Wavelength, nm	545	Ado concentration in neurons	50	
	Mouse	0.5	Healthy	Light	Cranial	14,400	Wavelength, nm	545	Adp concentration in neurons	25	
	Mouse	0.5	Healthy	Light	Cranial	14,400	Wavelength, nm	545	Amp concentration in neurons	88	
	Mouse	0.5	Healthy	Light	Cranial	14,400	Wavelength, nm	545	Imp concentration in neurons	57	
	Mouse	0.5	Healthy	Light	Cranial	14,400	Wavelength, nm	545	Hcy concentration in neurons	50	
	Mouse	0.5	Healthy	Light	Cranial	14,400	Wavelength, nm	545	Met concentration in neurons	92	
	Mouse	0.5	Healthy	Light	Cranial	14,400	Wavelength, nm	545	P-amk-alpha2 concentration	20	
	Mouse	0.5	Healthy	Light	Cranial	14,400	Wavelength, nm	545	Slow-wave sleep amount	133	
33	Mouse	0.25	Healthy	Light	Cranial	72,000	Wavelength, nm	630	Crossing numbers	40	[278]
	Mouse	0.25	SAMP8 aging model	Light	Cranial	72,000	Wavelength, nm	630	Crossing numbers	36	
	Mouse	0.25	Healthy	Light	Cranial	72,000	Wavelength, nm	630	Escape latency	16	
	Mouse	0.25	Healthy	Light	Cranial	72,000	Wavelength, nm	630	Brain fdh protein expression	20	
	Mouse	0.25	SAMP8 aging model	Light	Cranial	72,000	Wavelength, nm	630	Brain fdh protein expression	117	
	Mouse	0.25	Healthy	Light	Cranial	72,000	Wavelength, nm	630	Brain catalase protein expression	80	
	Mouse	0.25	SAMP8 aging model	Light	Cranial	72,000	Wavelength, nm	630	Brain catalase protein expression	255	
34	Mouse	0.2	Healthy	Light	Visually	3600	Wavelength, nm	555	M-cgf cytokine expression	100	[263]
	Mouse	0.2	Healthy	Light	Visually	3600	Wavelength, nm	555	Il-6 cytokine expression	500	
	Mouse	0.2	Healthy	Light	Visually	3600	Wavelength, nm	555	Mig cytokine expression	52	
	Mouse	0.2	Healthy	Light	Visually	3600	Wavelength, nm	555	Il-4 cytokine expression	77	
	Mouse	0.2	Healthy	Light	Visually	3600	Wavelength, nm	555	Ifn-gamma cytokine expression	2400	
	Mouse	0.2	Healthy	Light	Visually	3600	Wavelength, nm	555	Il-12 p70 cytokine expression	50	
	Mouse	0.2	Healthy	Light	Visually	3600	Wavelength, nm	555	Gm-csf cytokine expression	79	
	Mouse	0.2	Healthy	Light	Visually	3600	Wavelength, nm	555	Il-7 cytokine expression	100	
	Mouse	0.2	Healthy	Light	Visually	3600	Wavelength, nm	555	Il-1beta cytokine expression	22	
	Mouse	0.2	Healthy	Light	Visually	3600	Wavelength, nm	555	Gm-csf secretion	100	
	Mouse	0.2	Healthy	Light	Visually	3600	Wavelength, nm	555	Il-2 secretion	30	
	Mouse	0.2	Healthy	Light	Visually	3600	Wavelength, nm	555	Il-12 p70 secretion	60	
	Mouse	0.2	Healthy	Light	Visually	3600	Wavelength, nm	555	Eotaxin secretion	40	
	Mouse	0.2	Healthy	Light	Visually	3600	Wavelength, nm	555	Tnfapha secretion	100	
	Mouse	0.2	Healthy	Light	Visually	3600	Wavelength, nm	555	Mcp1 secretion	180	
35	Mouse	0.5	Healthy	Light	Visually	108,000	“-”	“-”	Number of shocks to learning	75	[206]
	Mouse	0.5	Healthy	Light	Visually	108,000	“-”	“-”	Brdu + cells number in dg region	89	
	Mouse	0.5	Healthy	Light	Visually	108,000	“-”	“-”	Dcx + cells number in dg region	86	
	Mouse	0.5	Healthy	Light	Visually	108,000	“-”	“-”	Brdu + dcx + cells number in dg region	70	
36	Mouse	0.1 7	BA	Lighting and audio	Visually	10	“-”	“-”	Abeta1-42 amyloid concentration solible	55	[37]
	Mouse	0.1 7	BA	Lighting and audio	Visually	10	“-”	“-”	Abeta1-42 amyloid concentration insolible	35	
	Mouse	0.1 7	BA	Lighting and audio	Visually	10	“-”	“-”	Plague numbers in ac	41	
	Mouse	0.1 7	BA	Audio	“-”	10	“-”	“-”	Plague numbers in ca1	50	
	Mouse	0.1 7	BA	Audio	“-”	10	“-”	“-”	Plague core area in ac	55	
	Mouse	0.1 7	BA	Audio	“-”	10	“-”	“-”	Plague core area in ca1	45	
	Mouse	0.1 7	BA	Audio	“-”	10	“-”	“-”	Abeta 12f4 amyloid concentration in ac	45	
	Mouse	0.1 7	BA	Audio	“-”	10	“-”	“-”	Abeta 12f4 amyloid concentration in ca1	40	
	Mouse	0.1 7	BA	Audio	“-”	10	“-”	“-”	Sb100b + astrocytes count in ac	26	
	Mouse	0.1 7	BA	Audio	“-”	10	“-”	“-”	Sb100b + astrocytes count in ca1	12	
	Mouse	0.1 7	BA	Audio	“-”	10	“-”	“-”	Gfab + astrocyte count in ac	38	
	Mouse	0.1 7	BA	Audio	“-”	10	“-”	“-”	Gfab + astrocyte count in ca1	17	
	Mouse	0.1 7	BA	Audio	“-”	10	“-”	“-”	Lrp1-abeta colocalization	157	
	Mouse	0.1 7	BA	Lighting and audio	Visually	10	“-”	“-”	Microglia cell body diameter	110	
	Mouse	0.1 7	BA	Lighting and audio	Visually	10	“-”	“-”	Microglia avarage processes leunght	25	
	Mouse	0.1 7	BA	Lighting and audio	Visually	10	“-”	“-”	Microglia count	95	
	Mouse	0.1 7	BA	Lighting and audio	Visually	10	“-”	“-”	Migroglia per plague rate	33	
37	Mouse	0.25	BA	Light	Visually	3600	Wavelength, nm	725	Microglia cell body diameter	75	[39]
	Mouse	0.25	BA	Light	Visually	3600	Wavelength, nm	725	Process length	40	
	Mouse	0.25	BA	Light	Visually	3600	Wavelength, nm	725	Alpha-beta + microglia	42	
	Mouse	0.25	BA	Light	Visually	3600	Wavelength, nm	725	Plague count	69	
	Mouse	0.25	BA	Light	Visually	3600	Wavelength, nm	725	Plague core area	65	
38	Mouse	0.8	BA	Light	Cranial	5,184,000	Wavelength, nm	1070	Discrimination index	22	[40]
	Mouse	0.8	BA	Light	Cranial	5,184,000	Wavelength, nm	1070	Latency in behavior test	26	
	Mouse	0.8	BA	Light	Cranial	5,184,000	Wavelength, nm	1070	Latency in behavior test	10	
	Mouse	0.8	BA	Light	Cranial	5,184,000	Wavelength, nm	1070	Number of plagues in hpc	25	
	Mouse	0.8	BA	Light	Cranial	5,184,000	Wavelength, nm	1070	Number of plagues in hpc	20	
	Mouse	0.8	BA	Light	Cranial	5,184,000	Wavelength, nm	1070	Number of plagues in ca1	43	
	Mouse	0.8	BA	Light	Cranial	5,184,000	Wavelength, nm	1070	Aplhabeta load in hpc	53	
	Mouse	0.8	BA	Light	Cranial	5,184,000	Wavelength, nm	1070	Aplhabeta load in ca1	33	
	Mouse	0.8	BA	Light	Cranial	5,184,000	Wavelength, nm	1070	Vessel-associated microglia	7	
	Mouse	0.8	BA	Light	Cranial	5,184,000	Wavelength, nm	1070	Vessel-associated microglia	11	
	Mouse	0.8	BA	Light	Cranial	5,184,000	Wavelength, nm	1070	Perivascilar microglia	9	
	Mouse	0.8	BA	Light	Cranial	5,184,000	Wavelength, nm	1070	Perivascilar microglia	9	
	Mouse	0.8	BA	Light	Cranial	5,184,000	Wavelength, nm	1070	Pverg concentration	40	
	Mouse	0.8	BA	Light	Cranial	5,184,000	Wavelength, nm	1070	Perk concentration	45	
39	Mouse	0.25	Stroke	Light	Cranial	2880	Wavelength, nm	473	Total eeg power in m1	150	[41]
	Mouse	0.25	Stroke	Light	Cranial	2880	Wavelength, nm	473	Total eeg power in pta	39	
	Mouse	0.25	Stroke	Light	Cranial	2880	Wavelength, nm	473	Theta rhythm power in m1	120	
	Mouse	0.25	Stroke	Light	Cranial	2880	Wavelength, nm	473	Theta rhythm power in pta	300	
	Mouse	0.25	Stroke	Light	Cranial	2880	Wavelength, nm	473	Beta rhythm power in m1	150	
	Mouse	0.25	Stroke	Light	Cranial	2880	Wavelength, nm	473	Beta rhythm power in pta	700	
	Mouse	0.25	Stroke	Light	Cranial	2880	Wavelength, nm	473	Gamma rhythm power in m1	150	
	Mouse	0.25	Stroke	Light	Cranial	2880	Wavelength, nm	473	Gamma rhythm power in pta	233	
	Mouse	0.25	Stroke	Light	Cranial	2880	Wavelength, nm	473	Blood flow	33	
	Mouse	0.25	Stroke	Light	Cranial	2880	Wavelength, nm	473	Brain swelling	125	
	Mouse	0.25	Stroke	Light	Cranial	2880	Wavelength, nm	473	Contralesional foot faults	60	
	Mouse	0.25	Stroke	Light	Cranial	2880	Wavelength, nm	473	Contralesional foot faults	80	
	Mouse	0.25	Stroke	Light	Cranial	2880	Wavelength, nm	473	Neurodeficite scope	50	
40	Mouse	0.25	BA	Light	Cranial	3600	Wavelength, nm	4793	Alpha beta1-40 amyloid content	55	[58]
	Mouse	0.25	BA	Light	Cranial	3600	Wavelength, nm	4793	Alpha beta1-42 amyloid content	45	
	Mouse	0.25	BA	Light	Cranial	3600	Wavelength, nm	4793	App ntf content	25	
	Mouse	0.25	BA	Light	Cranial	3600	Wavelength, nm	4793	App ctfs content	20	
	Mouse	0.25	BA	Light	Cranial	3600	Wavelength, nm	4793	Aplha beta maen intensity value	40	
	Mouse	0.25	BA	Light	Cranial	3600	Wavelength, nm	4793	Eea1 mean intensity value	35	
	Mouse	0.25	BA	Light	Cranial	3600	Wavelength, nm	4793	Csf1 gene expression	340	
	Mouse	0.25	BA	Light	Cranial	3600	Wavelength, nm	4793	Csf11r gene expression	230	
	Mouse	0.25	BA	Light	Cranial	3600	Wavelength, nm	4793	Csf2ra gene expression	50	
	Mouse	0.25	BA	Light	Cranial	3600	Wavelength, nm	4793	Icam1 gene expression	40	
	Mouse	0.25	BA	Light	Cranial	3600	Wavelength, nm	4793	B2m	250	
	Mouse	0.25	BA	Light	Cranial	3600	Wavelength, nm	4793	Spp1 gene expression	120	
	Mouse	0.25	BA	Light	Cranial	3600	Wavelength, nm	4793	Lyz2 gene expression	290	
	Mouse	0.25	BA	Light	Cranial	3600	Wavelength, nm	4793	Cd68 gene expression	210	
	Mouse	0.25	BA	Light	Cranial	3600	Wavelength, nm	4793	Irf7 gene expression	220	
	Mouse	0.25	BA	Light	Cranial	3600	Wavelength, nm	4793	Bst2 gene expression	130	
	Mouse	0.25	BA	Light	Cranial	3600	Wavelength, nm	4793	Microglia count	114	
	Mouse	0.25	BA	Light	Cranial	3600	Wavelength, nm	4793	Microglia aβ+	187	
	Mouse	0.25	BA	Light	Cranial	3600	Wavelength, nm	4793	Microglia cell body diameter	130	
	Mouse	0.25	BA	Light	Cranial	3600	Wavelength, nm	4793	Process length	60	
	Mouse	0.25	BA	Light	Visually	3600	Wavelength, nm	555	Alpha beta1-40 amyloid content	65	
	Mouse	0.25	BA	Light	Visually	3600	Wavelength, nm	555	Alpha beta1-42 amyloid content	70	
	Mouse	0.25	BA	Light	Visually	3600	Wavelength, nm	555	Microglia cell body diameter	70	
	Mouse	0.25	BA	Light	Visually	3600	Wavelength, nm	555	Process length	40	
	Mouse	0.25	BA	Light	Visually	3600	Wavelength, nm	555	Microglia aβ+	57	
	Mouse	0.25	BA	Light	Visually	3600	Wavelength, nm	555	Plaque number	64	
	Mouse	0.25	BA	Light	Visually	3600	Wavelength, nm	555	Plaque core area	65	
41	Rhesus macaque	3	Healthy	Light	Visually	10	Michelson contrast, %	2	Gamma-rhythm power by meg	5	[117]
	Rhesus macaque	3	Healthy	Light	Visually	10	Michelson contrast, %	3.5	Gamma-rhythm power by meg	5	
	Rhesus macaque	3	Healthy	Light	Visually	10	Michelson contrast, %	6	Gamma-rhythm power by meg	10	
	Rhesus macaque	3	Healthy	Light	Visually	10	Michelson contrast, %	9.7	Gamma-rhythm power by meg	20	
	Rhesus macaque	3	Healthy	Light	Visually	10	Michelson contrast, %	16.3	Gamma-rhythm power by meg	160	
	Rhesus macaque	3	Healthy	Light	Visually	10	Michelson contrast, %	35.9	Gamma-rhythm power by meg	390	
	Rhesus macaque	3	Healthy	Light	Visually	10	Michelson contrast, %	50.3	Gamma-rhythm power by meg	500	
	Rhesus macaque	3	Healthy	Light	Visually	10	Michelson contrast, %	72	Gamma-rhythm power by meg	630	
42	Rhesus macaque	3	Healthy	Light	Visually	10	Michelson contrast, %	50.3	Gamma-rhythm power by meg in v1 lfp	600	[116]
	Rhesus macaque	3	Healthy	Light	Visually	10	Michelson contrast, %	35.9	Gamma-rhythm power by meg in v1 lfp	1000	
	Rhesus macaque	3	Healthy	Light	Visually	10	Michelson contrast, %	16.3	Gamma-rhythm power by meg in v1 lfp	550	
	Rhesus macaque	3	Healthy	Light	Visually	10	Michelson contrast, %	9.7	Gamma-rhythm power by meg in v1 lfp	200	
	Rhesus macaque	3	Healthy	Light	Visually	10	Michelson contrast, %	6.1	Gamma-rhythm power by meg in v1 lfp	150	
	Rhesus macaque	3	Healthy	Light	Visually	10	Michelson contrast, %	3.7	Gamma-rhythm power by meg in v1 lfp	20	

## References

[1] Gudkov S.V., Sarimov R.M., Astashev M.E., Pishchalnikov R.Y., Yanykin D.V., Simakin A.V., Shkirin A.V., Serov D.A., Konchekov E.M., Gusein-zade Namik Guseynaga O. (2023). Modern physical methods and technologies in agriculture. Physics-Uspekhi.

[2] Semenova N.A., Burmistrov D.E., Shumeyko S.A., Gudkov S.V. (2024). Fertilizers Based on Nanoparticles as Sources of Macro- and Microelements for Plant Crop Growth: A Review. Agronomy.

[3] Burmistrov D.E., Shumeyko S.A., Semenova N.A., Dorokhov A.S., Gudkov S.V. (2025). Selenium Nanoparticles (Se NPs) as Agents for Agriculture Crops with Multiple Activity: A Review. Agronomy.

[4] Burmistrov D.E., Gudkov S.V., Franceschi C., Vedunova M.V. (2024). Sex as a Determinant of Age-Related Changes in the Brain. Int. J. Mol. Sci..

[5] Gudkov S.V., Burmistrov D.E., Kondakova E.V., Sarimov R.M., Yarkov R.S., Franceschi C., Vedunova M.V. (2023). An emerging role of astrocytes in aging/neuroinflammation and gut-brain axis with consequences on sleep and sleep disorders. Ageing Res. Rev..

[6] Nichols E., Steinmetz J.D., Vollset S.E., Fukutaki K., Chalek J., Abd-Allah F., Abdoli A., Abualhasan A., Abu-Gharbieh E., Akram T.T. (2022). Estimation of the global prevalence of dementia in 2019 and forecasted prevalence in 2050: An analysis for the Global Burden of Disease Study 2019. Lancet Public Health.

[7] Cummings J., Lee G., Zhong K., Fonseca J., Taghva K. (2021). Alzheimer’s disease drug development pipeline: 2021. Alzheimer’s Dement. Transl. Res. Clin. Interv..

[8] Xu J., Zhang Y., Qiu C., Cheng F. (2017). Global and regional economic costs of dementia: A systematic review. Lancet.

[9] Cole S.R., Voytek B. (2017). Brain Oscillations and the Importance of Waveform Shape. Trends Cogn. Sci..

[10] Mathalon D.H., Sohal V.S. (2015). Neural Oscillations and Synchrony in Brain Dysfunction and Neuropsychiatric Disorders. JAMA Psychiatry.

[11] Zhang H., Watrous A.J., Patel A., Jacobs J. (2018). Theta and Alpha Oscillations Are Traveling Waves in the Human Neocortex. Neuron.

[12] Guan A., Wang S., Huang A., Qiu C., Li Y., Li X., Wang J., Wang Q., Deng B. (2022). The role of gamma oscillations in central nervous system diseases: Mechanism and treatment. Front. Cell. Neurosci..

[13] Başar E., Başar-Eroğlu C., Karakaş S., Schürmann M. (2000). Brain oscillations in perception and memory. Int. J. Psychophysiol..

[14] Roux F., Wibral M., Mohr H.M., Singer W., Uhlhaas P.J. (2012). Gamma-Band Activity in Human Prefrontal Cortex Codes for the Number of Relevant Items Maintained in Working Memory. J. Neurosci..

[15] Swanson O.K., Maffei A. (2019). From Hiring to Firing: Activation of Inhibitory Neurons and Their Recruitment in Behavior. Front. Mol. Neurosci..

[16] Eckhorn R., Bauer R., Jordan W., Brosch M., Kruse W., Munk M., Reitboeck H.J. (1988). Coherent oscillations: A mechanism of feature linking in the visual cortex?. Biol. Cybern..

[17] Ahmed O.J., Cash S.S. (2013). Finding synchrony in the desynchronized EEG: The history and interpretation of gamma rhythms. Front. Integr. Neurosci..

[18] Wu L., Wei Y., He K., Gao Q. (2025). The Effects and Safety of Gamma Rhythm Stimulation on Cognitive Function in Alzheimer’s Disease: A Systematic Review and Meta-Analysis. Neurorehabil. Neural Repair.

[19] Deng Q., Wu C., Parker E., Zhu J., Liu T.C.-Y., Duan R., Yang L. (2024). Mystery of gamma wave stimulation in brain disorders. Mol. Neurodegener..

[20] Tiesinga P.H., Fellous J.-M., Salinas E., José J.V., Sejnowski T.J. (2004). Inhibitory synchrony as a mechanism for attentional gain modulation. J. Physiol.-Paris.

[21] Antonoudiou P., Tan Y.L., Kontou G., Upton A.L., Mann E.O. (2020). Parvalbumin and Somatostatin Interneurons Contribute to the Generation of Hippocampal Gamma Oscillations. J. Neurosci..

[22] Buzsáki G., Wang X.-J. (2012). Mechanisms of Gamma Oscillations. Annu. Rev. Neurosci..

[23] Takada N., Pi H.J., Sousa V.H., Waters J., Fishell G., Kepecs A., Osten P. (2014). A developmental cell-type switch in cortical interneurons leads to a selective defect in cortical oscillations. Nat. Commun..

[24] Adaikkan C., Tsai L.-H. (2020). Gamma Entrainment: Impact on Neurocircuits, Glia, and Therapeutic Opportunities. Trends Neurosci..

[25] Beyna T., Gerges C. (2020). Clinical Management of Bile Duct Diseases: Role of Endoscopic Ultrasound in a Personalized Approach. J. Pers. Med..

[26] Lasztóczi B., Klausberger T. (2014). Layer-Specific GABAergic Control of Distinct Gamma Oscillations in the CA1 Hippocampus. Neuron.

[27] Muller M., Rouleau E.A.M.Y., van der Gaag S., Zutt R., Hoffmann C.F., van der Gaag N.A., van Essen T.A., Schouten A.C., Contarino M.F. (2025). Decoding finely-tuned gamma oscillations in chronic deep brain stimulation for Parkinson’s disease. medRxiv.

[28] Olaru M., Hahn A., Shcherbakova M., Little S., Neumann W.-J., Abbasi-Asl R., Starr P.A. (2025). Deep brain stimulation-entrained gamma oscillations in chronic home recordings in Parkinson’s disease. Brain Stimul..

[29] Park J.M., Tsai L.-H. (2025). Innovations in noninvasive sensory stimulation treatments to combat Alzheimer’s disease. PLoS Biol..

[30] Traikapi A., Konstantinou N. (2021). Gamma Oscillations in Alzheimer’s Disease and Their Potential Therapeutic Role. Front. Syst. Neurosci..

[31] Rojas D.C., Wilson L.B. (2014). γ-Band Abnormalities as Markers of Autism Spectrum Disorders. Biomark. Med..

[32] Metzner C., Mäki-Marttunen T., Karni G., McMahon-Cole H., Steuber V. (2022). The effect of alterations of schizophrenia-associated genes on gamma band oscillations. Schizophrenia.

[33] McNally J.M., McCarley R.W. (2016). Gamma band oscillations. Curr. Opin. Psychiatry.

[34] Palop J.J., Mucke L. (2016). Network abnormalities and interneuron dysfunction in Alzheimer disease. Nat. Rev. Neurosci..

[35] Strüber D., Herrmann C.S. (2020). Modulation of gamma oscillations as a possible therapeutic tool for neuropsychiatric diseases: A review and perspective. Int. J. Psychophysiol..

[36] Manippa V., Palmisano A., Filardi M., Vilella D., Nitsche M.A., Rivolta D., Logroscino G. (2022). An update on the use of gamma (multi)sensory stimulation for Alzheimer’s disease treatment. Front. Aging Neurosci..

[37] Martorell A.J., Paulson A.L., Suk H.-J., Abdurrob F., Drummond G.T., Guan W., Young J.Z., Kim D.N.-W., Kritskiy O., Barker S.J. (2019). Multi-sensory Gamma Stimulation Ameliorates Alzheimer’s-Associated Pathology and Improves Cognition. Cell.

[38] Soula M., Martín-Ávila A., Zhang Y., Dhingra A., Nitzan N., Sadowski M.J., Gan W.-B., Buzsáki G. (2023). Forty-hertz light stimulation does not entrain native gamma oscillations in Alzheimer’s disease model mice. Nat. Neurosci..

[39] Singer A.C., Martorell A.J., Douglas J.M., Abdurrob F., Attokaren M.K., Tipton J., Mathys H., Adaikkan C., Tsai L.-H. (2018). Noninvasive 40-Hz light flicker to recruit microglia and reduce amyloid beta load. Nat. Protoc..

[40] Tao L., Liu Q., Zhang F., Fu Y., Zhu X., Weng X., Han H., Huang Y., Suo Y., Chen L. (2021). Microglia modulation with 1070-nm light attenuates Aβ burden and cognitive impairment in Alzheimer’s disease mouse model. Light Sci. Appl..

[41] Balbi M., Xiao D., Jativa Vega M., Hu H., Vanni M.P., Bernier L.-P., LeDue J., MacVicar B., Murphy T.H. (2021). Gamma frequency activation of inhibitory neurons in the acute phase after stroke attenuates vascular and behavioral dysfunction. Cell Rep..

[42] Obleser J., Kayser C. (2019). Neural Entrainment and Attentional Selection in the Listening Brain. Trends Cogn. Sci..

[43] Sheer D.E. (1989). Focused arousal and the cognitive 40-Hz event-related potentials: Differential diagnosis of Alzheimer’s disease. Prog. Clin. Biol. Res..

[44] Bressler S.L., Freeman W.J. (1980). Frequency analysis of olfactory system EEG in cat, rabbit, and rat. Electroencephalogr. Clin. Neurophysiol..

[45] Galambos R., Makeig S., Talmachoff P.J. (1981). A 40-Hz auditory potential recorded from the human scalp. Proc. Natl. Acad. Sci. USA.

[46] Ferster D. (1986). Orientation selectivity of synaptic potentials in neurons of cat primary visual cortex. J. Neurosci..

[47] Bouyer J.J., Montaron M.F., Vahnée J.M., Albert M.P., Rougeul A. (1987). Anatomical localization of cortical beta rhythms in cat. Neuroscience.

[48] Gray C.M., Singer W. (1989). Stimulus-specific neuronal oscillations in orientation columns of cat visual cortex. Proc. Natl. Acad. Sci. USA.

[49] Gray C.M., König P., Engel A.K., Singer W. (1989). Oscillatory responses in cat visual cortex exhibit inter-columnar synchronization which reflects global stimulus properties. Nature.

[50] Engel A.K., König P., Kreiter A.K., Schillen T.B., Singer W. (1992). Temporal coding in the visual cortex: New vistas on integration in the nervous system. Trends Neurosci..

[51] Traub R.D., Whittington M.A., Stanford I.M., Jefferys J.G.R. (1996). A mechanism for generation of long-range synchronous fast oscillations in the cortex. Nature.

[52] Traub R.D., Jefferys J.G.R., Whittington M.A. (1997). Simulation of Gamma Rhythms in Networks of Interneurons and Pyramidal Cells. J. Comput. Neurosci..

[53] Elliott M.A., Müller H.J. (1998). Synchronous Information Presented in 40-HZ Flicker Enhances Visual Feature Binding. Psychol. Sci..

[54] Herrmann C.S. (2001). Human EEG responses to 1–100 Hz flicker: Resonance phenomena in visual cortex and their potential correlation to cognitive phenomena. Exp. Brain Res..

[55] Hadler M.D., Tzilivaki A., Schmitz D., Alle H., Geiger J.R.P. (2024). Gamma oscillation plasticity is mediated via parvalbumin interneurons. Sci. Adv..

[56] Onorato I., Tzanou A., Schneider M., Uran C., Broggini A.C., Vinck M. (2025). Distinct roles of PV and Sst interneurons in visually induced gamma oscillations. Cell Rep..

[57] Zhang N., Hu B.-W., Li X.-M., Huang H. (2025). Rethinking parvalbumin: From passive marker to active modulator of hippocampal circuits. IBRO Neurosci. Rep..

[58] Iaccarino H.F., Singer A.C., Martorell A.J., Rudenko A., Gao F., Gillingham T.Z., Mathys H., Seo J., Kritskiy O., Abdurrob F. (2016). Gamma frequency entrainment attenuates amyloid load and modifies microglia. Nature.

[59] Rajji T.K., Chan D., Suk H.-J., Jackson B.L., Milman N.P., Stark D., Klerman E.B., Kitchener E., Fernandez Avalos V.S., de Weck G. (2022). Gamma frequency sensory stimulation in mild probable Alzheimer’s dementia patients: Results of feasibility and pilot studies. PLoS ONE.

[60] Tian T., Qin X., Wang Y., Shi Y., Yang X. (2021). 40 Hz Light Flicker Promotes Learning and Memory via Long Term Depression in Wild-Type Mice. J. Alzheimer’s Dis..

[61] Blanco-Duque C., Chan D., Kahn M.C., Murdock M.H., Tsai L.H. (2023). Audiovisual gamma stimulation for the treatment of neurodegeneration. J. Intern. Med..

[62] Adaikkan C., Middleton S.J., Marco A., Pao P.-C., Mathys H., Kim D.N.-W., Gao F., Young J.Z., Suk H.-J., Boyden E.S. (2019). Gamma Entrainment Binds Higher-Order Brain Regions and Offers Neuroprotection. Neuron.

[63] Cimenser A., Hempel E., Travers T., Strozewski N., Martin K., Malchano Z., Hajós M. (2021). Sensory-Evoked 40-Hz Gamma Oscillation Improves Sleep and Daily Living Activities in Alzheimer’s Disease Patients. Front. Syst. Neurosci..

[64] Jirakittayakorn N., Wongsawat Y. (2017). Brain responses to 40-Hz binaural beat and effects on emotion and memory. Int. J. Psychophysiol..

[65] Li K.T., Liang J., Zhou C., Su J. (2021). Gamma Oscillations Facilitate Effective Learning in Excitatory-Inhibitory Balanced Neural Circuits. Neural Plast..

[66] Park S.-S., Park H.-S., Kim C.-J., Kang H.-S., Kim D.-H., Baek S.-S., Kim T.-W. (2020). Physical exercise during exposure to 40-Hz light flicker improves cognitive functions in the 3xTg mouse model of Alzheimer’s disease. Alzheimer’s Res. Ther..

[67] Prichard A., Garza K.M., Shridhar A., He C., Bitarafan S., Pybus A., Wang Y., Snyder E., Goodson M.C., Franklin T.C. (2023). Brain rhythms control microglial response and cytokine expression via NF-κB signaling. Sci. Adv..

[68] Ross B., Fujioka T. (2016). 40-Hz oscillations underlying perceptual binding in young and older adults. Psychophysiology.

[69] Zheng L., Yu M., Lin R., Wang Y., Zhuo Z., Cheng N., Wang M., Tang Y., Wang L., Hou S.-T. (2020). Rhythmic light flicker rescues hippocampal low gamma and protects ischemic neurons by enhancing presynaptic plasticity. Nat. Commun..

[70] Murdock M.H., Yang C.-Y., Sun N., Pao P.-C., Blanco-Duque C., Kahn M.C., Kim T., Lavoie N.S., Victor M.B., Islam M.R. (2024). Multisensory gamma stimulation promotes glymphatic clearance of amyloid. Nature.

[71] Sarimov R.M., Serov D.A., Gudkov S.V. (2023). Biological Effects of Magnetic Storms and ELF Magnetic Fields. Biology.

[72] Park Y., Lee K., Park J., Bae J.B., Kim S.-S., Kim D.-W., Woo S.J., Yoo S., Kim K.W. (2022). Optimal flickering light stimulation for entraining gamma rhythms in older adults. Sci. Rep..

[73] Hashim A.A., Majid M.A., Mustafa B.A. Legibility of Web Page on Full High Definition Display. Proceedings of the 2013 International Conference on Advanced Computer Science Applications and Technologies.

[74] Sánchez-Lacambra M., Orduna-Hospital E., Arcas-Carbonell M., Sánchez-Cano A. (2024). Effects of Light on Visual Function, Alertness, and Cognitive Performance: A Computerized Test Assessment. Appl. Sci..

[75] Vetter C., Pattison P.M., Houser K., Herf M., Phillips A.J.K., Wright K.P., Skene D.J., Brainard G.C., Boivin D.B., Glickman G. (2021). A Review of Human Physiological Responses to Light: Implications for the Development of Integrative Lighting Solutions. Leukos.

[76] Dittmar M. (2001). Changing Colour Preferences with Ageing: A Comparative Study on Younger and Older Native Germans Aged 19–90 Years. Gerontology.

[77] Spitschan M., Yokoyama S., Tanaka Y., Kojima T., Horai R., Kato Y., Nakamura H., Sato H., Mitamura M., Tanaka K. (2021). Age-related changes of color visual acuity in normal eyes. PLoS ONE.

[78] Klimesch W. (2013). An algorithm for the EEG frequency architecture of consciousness and brain body coupling. Front. Hum. Neurosci..

[79] Fries P. (2015). Rhythms for Cognition: Communication through Coherence. Neuron.

[80] Murty D.V.P.S., Shirhatti V., Ravishankar P., Ray S. (2018). Large Visual Stimuli Induce Two Distinct Gamma Oscillations in Primate Visual Cortex. J. Neurosci..

[81] Arutiunian V., Arcara G., Buyanova I., Gomozova M., Dragoy O. (2022). The age-related changes in 40 Hz Auditory Steady-State Response and sustained Event-Related Fields to the same amplitude-modulated tones in typically developing children: A magnetoencephalography study. Hum. Brain Mapp..

[82] Grachev I.D., Apkarian A.V. (2008). Aging alters regional multichemical profile of the human brain: An in vivo1H-MRS study of young versus middle-aged subjects. J. Neurochem..

[83] Muthukumaraswamy S.D., Singh K.D. (2008). Spatiotemporal frequency tuning of BOLD and gamma band MEG responses compared in primary visual cortex. NeuroImage.

[84] Bennett P.J., Sekuler R., Sekuler A.B. (2007). The effects of aging on motion detection and direction identification. Vis. Res..

[85] Betts L.R., Taylor C.P., Sekuler A.B., Bennett P.J. (2005). Aging Reduces Center-Surround Antagonism in Visual Motion Processing. Neuron.

[86] Leventhal A.G., Wang Y., Pu M., Zhou Y., Ma Y. (2003). GABA and Its Agonists Improved Visual Cortical Function in Senescent Monkeys. Science.

[87] Schmolesky M.T., Wang Y., Pu M., Leventhal A.G. (2000). Degradation of stimulus selectivity of visual cortical cells in senescent rhesus monkeys. Nat. Neurosci..

[88] Murty D.V.P.S., Manikandan K., Kumar W.S., Ramesh R.G., Purokayastha S., Javali M., Rao N.P., Ray S. (2020). Gamma oscillations weaken with age in healthy elderly in human EEG. NeuroImage.

[89] Gudkov S.V., Pustovoy V.I., Sarimov R.M., Serov D.A., Simakin A.V., Shcherbakov I.A. (2025). Diversity of Effects of Mechanical Influences on Living Systems and Aqueous Solutions. Int. J. Mol. Sci..

[90] Davey Z., Gupta P.B., Li D.R., Nayak R.U., Govindarajan P. (2022). Rapid Response EEG: Current State and Future Directions. Curr. Neurol. Neurosci. Rep..

[91] Rohenkohl G., Bosman C.A., Fries P. (2018). Gamma Synchronization between V1 and V4 Improves Behavioral Performance. Neuron.

[92] Astashev M., Serov D., Gudkov S. (2023). Application of Spectral Methods of Analysis for Description of Ultradian Biorhythms at the Levels of Physiological Systems, Cells and Molecules (Review). Mathematics.

[93] Pack C., Han C., Wang T., Yang Y., Wu Y., Li Y., Dai W., Zhang Y., Wang B., Yang G. (2021). Multiple gamma rhythms carry distinct spatial frequency information in primary visual cortex. PLoS Biol..

[94] Misselhorn J., Schwab B.C., Schneider T.R., Engel A.K. (2019). Synchronization of Sensory Gamma Oscillations Promotes Multisensory Communication. eNeuro.

[95] Jones M., McDermott B., Oliveira B.L., O’Brien A., Coogan D., Lang M., Moriarty N., Dowd E., Quinlan L., Mc Ginley B. (2019). Gamma Band Light Stimulation in Human Case Studies: Groundwork for Potential Alzheimer’s Disease Treatment. J. Alzheimer’s Dis..

[96] Lee K., Park Y., Suh S.W., Kim S.-S., Kim D.-W., Lee J., Park J., Yoo S., Kim K.W. (2021). Optimal flickering light stimulation for entraining gamma waves in the human brain. Sci. Rep..

[97] Min B.-K., Dähne S., Ahn M.-H., Noh Y.-K., Müller K.-R. (2016). Decoding of top-down cognitive processing for SSVEP-controlled BMI. Sci. Rep..

[98] Regan D. (1966). An Effect of Stimulus Colour on Average Steady-state Potentials evoked in Man. Nature.

[99] Tello R.J.M.G., Müller S.M.T., Ferreira A., Bastos T.F. (2015). Comparison of the influence of stimuli color on Steady-State Visual Evoked Potentials. Res. Biomed. Eng..

[100] Roorda A., Williams D.R. (1999). The arrangement of the three cone classes in the living human eye. Nature.

[101] Roorda A., Metha A.B., Lennie P., Williams D.R. (2001). Packing arrangement of the three cone classes in primate retina. Vis. Res..

[102] Stauch B.J., Peter A., Ehrlich I., Nolte Z., Fries P. (2022). Human visual gamma for color stimuli. eLife.

[103] Chu L., Fernández-Vargas J., Kita K., Yu W. (2017). Influence of Stimulus Color on Steady State Visual Evoked Potentials. Intelligent Autonomous Systems 14.

[104] Noda Y., Takano M., Hayano M., Li X., Wada M., Nakajima S., Mimura M., Kondo S., Tsubota K. (2021). Photobiological Neuromodulation of Resting-State EEG and Steady-State Visual-Evoked Potentials by 40 Hz Violet Light Optical Stimulation in Healthy Individuals. J. Pers. Med..

[105] Osorio D., Wuerger S. (2013). Colour Constancy Across the Life Span: Evidence for Compensatory Mechanisms. PLoS ONE.

[106] Wijk H., Berg S., Sivik L., Steen B. (1999). Color discrimination, color naming and color preferences in 80-year olds. Aging Clin. Exp. Res..

[107] Raven F., Aton S.J. (2021). The Engram’s Dark Horse: How Interneurons Regulate State-Dependent Memory Processing and Plasticity. Front. Neural Circuits.

[108] Kloc M., Maffei A. (2014). Target-Specific Properties of Thalamocortical Synapses onto Layer 4 of Mouse Primary Visual Cortex. J. Neurosci..

[109] Abubaker M., Al Qasem W., Pilátová K., Ježdík P., Kvašňák E. (2024). Theta-gamma-coupling as predictor of working memory performance in young and elderly healthy people. Mol. Brain.

[110] Cardin J.A., Carlén M., Meletis K., Knoblich U., Zhang F., Deisseroth K., Tsai L.-H., Moore C.I. (2009). Driving fast-spiking cells induces gamma rhythm and controls sensory responses. Nature.

[111] Carlén M., Meletis K., Siegle J.H., Cardin J.A., Futai K., Vierling-Claassen D., Rühlmann C., Jones S.R., Deisseroth K., Sheng M. (2011). A critical role for NMDA receptors in parvalbumin interneurons for gamma rhythm induction and behavior. Mol. Psychiatry.

[112] Gulyás A.I., Szabó G.G., Ulbert I., Holderith N., Monyer H., Erdélyi F., Szabó G., Freund T.F., Hájos N. (2010). Parvalbumin-Containing Fast-Spiking Basket Cells Generate the Field Potential Oscillations Induced by Cholinergic Receptor Activation in the Hippocampus. J. Neurosci..

[113] Whittington M.A., Traub R.D., Kopell N., Ermentrout B., Buhl E.H. (2000). Inhibition-based rhythms: Experimental and mathematical observations on network dynamics. Int. J. Psychophysiol..

[114] Segneri M., Bi H., Olmi S., Torcini A. (2020). Theta-Nested Gamma Oscillations in Next Generation Neural Mass Models. Front. Comput. Neurosci..

[115] Friedman-Hill S. (2000). Dynamics of Striate Cortical Activity in the Alert Macaque: I. Incidence and Stimulus-dependence of Gamma-band Neuronal Oscillations. Cereb. Cortex.

[116] Ermentrout B., Lowet E., Roberts M., Hadjipapas A., Peter A., van der Eerden J., De Weerd P. (2015). Input-Dependent Frequency Modulation of Cortical Gamma Oscillations Shapes Spatial Synchronization and Enables Phase Coding. PLoS Comput. Biol..

[117] Hadjipapas A., Lowet E., Roberts M.J., Peter A., De Weerd P. (2015). Parametric variation of gamma frequency and power with luminance contrast: A comparative study of human MEG and monkey LFP and spike responses. NeuroImage.

[118] Roberts M.J., Lowet E., Brunet N.M., Ter Wal M., Tiesinga P., Fries P., De Weerd P. (2013). Robust Gamma Coherence between Macaque V1 and V2 by Dynamic Frequency Matching. Neuron.

[119] Ferando I., Mody I. (2015). In vitro gamma oscillations following partial and complete ablation of δ subunit-containing GABAA receptors from parvalbumin interneurons. Neuropharmacology.

[120] Mann E.O., Mody I. (2009). Control of hippocampal gamma oscillation frequency by tonic inhibition and excitation of interneurons. Nat. Neurosci..

[121] Robson S.E., Muthukumarawswamy S.D., John Evans C., Shaw A., Brealy J., Davis B., McNamara G., Perry G., Singh K.D. (2015). Structural and neurochemical correlates of individual differences in gamma frequency oscillations in human visual cortex. J. Anat..

[122] Gaetz W., Edgar J.C., Wang D.J., Roberts T.P.L. (2011). Relating MEG measured motor cortical oscillations to resting γ-Aminobutyric acid (GABA) concentration. NeuroImage.

[123] Khachatryan E., Wittevrongel B., Reinartz M., Dauwe I., Carrette E., Meurs A., Van Roost D., Boon P., Van Hulle M.M. (2022). Cognitive tasks propagate the neural entrainment in response to a visual 40 Hz stimulation in humans. Front. Aging Neurosci..

[124] Jeffries K.J., Fritz J.B., Braun A.R. (2003). Words in melody: An H215O PET study of brain activation during singing and speaking. NeuroReport.

[125] Kaiser J., Hertrich I., Ackermann H., Mathiak K., Lutzenberger W. (2005). Hearing Lips: Gamma-band Activity During Audiovisual Speech Perception. Cereb. Cortex.

[126] Kaiser J., Lutzenberger W. (2003). Induced Gamma-Band Activity and Human Brain Function. Neurosci..

[127] Kaiser J., Ripper B., Birbaumer N., Lutzenberger W. (2003). Dynamics of gamma-band activity in human magnetoencephalogram during auditory pattern working memory. NeuroImage.

[128] Lu Y., Sarter M., Zochowski M., Booth V. (2020). Phasic cholinergic signaling promotes emergence of local gamma rhythms in excitatory–inhibitory networks. Eur. J. Neurosci..

[129] Howe W.M., Gritton H.J., Lusk N.A., Roberts E.A., Hetrick V.L., Berke J.D., Sarter M. (2017). Acetylcholine Release in Prefrontal Cortex Promotes Gamma Oscillations and Theta–Gamma Coupling during Cue Detection. J. Neurosci..

[130] Bianciardi B., Uhlhaas P.J. (2021). Do NMDA-R antagonists re-create patterns of spontaneous gamma-band activity in schizophrenia? A systematic review and perspective. Neurosci. Biobehav. Rev..

[131] Cornford J.H., Mercier M.S., Leite M., Magloire V., Häusser M., Kullmann D.M. (2019). Dendritic NMDA receptors in parvalbumin neurons enable strong and stable neuronal assemblies. eLife.

[132] Jadi M.P., Behrens M.M., Sejnowski T.J. (2016). Abnormal Gamma Oscillations in N-Methyl-D-Aspartate Receptor Hypofunction Models of Schizophrenia. Biol. Psychiatry.

[133] Chen W., Luo B., Gao N., Li H., Wang H., Li L., Cui W., Zhang L., Sun D., Liu F. (2021). Neddylation stabilizes Nav1.1 to maintain interneuron excitability and prevent seizures in murine epilepsy models. J. Clin. Investig..

[134] Martinez-Losa M., Tracy T.E., Ma K., Verret L., Clemente-Perez A., Khan A.S., Cobos I., Ho K., Gan L., Mucke L. (2018). Nav1.1-Overexpressing Interneuron Transplants Restore Brain Rhythms and Cognition in a Mouse Model of Alzheimer’s Disease. Neuron.

[135] Bressler S.L., Menon V. (2010). Large-scale brain networks in cognition: Emerging methods and principles. Trends Cogn. Sci..

[136] Menon V. (2011). Large-scale brain networks and psychopathology: A unifying triple network model. Trends Cogn. Sci..

[137] Kuptsov P.V., Stankevich N.V. (2025). Reconstruction of neuromorphic dynamics from a single scalar time series using variational autoencoder and neural network map. Chaos Solitons Fractals.

[138] Orekhova E.V., Butorina A.V., Sysoeva O.V., Prokofyev A.O., Nikolaeva A.Y., Stroganova T.A. (2015). Frequency of gamma oscillations in humans is modulated by velocity of visual motion. J. Neurophysiol..

[139] Bressler S. (2008). Neurocognitive networks. Scholarpedia.

[140] Pechenkova E., Rachinskaya M., Vasilenko V., Blazhenkova O., Mershina E. (2025). Brain Functional Connectivity During First- and Third-Person Visual Imagery. Vision.

[141] Kiviniemi V., Tervonen O., Timonen M., Nissilä J., Nikkinen J., Starck T., Remes J., Littow H., Abou Elseoud A. (2011). Group-ICA Model Order Highlights Patterns of Functional Brain Connectivity. Front. Syst. Neurosci..

[142] Sinitsyn D.O., Bakulin I.S., Poydasheva A.G., Legostaeva L.A., Kremneva E.I., Lagoda D.Y., Chernyavskiy A.Y., Medyntsev A.A., Suponeva N.A., Piradov M.A. (2020). Brain Activations and Functional Connectivity Patterns Associated with Insight-Based and Analytical Anagram Solving. Behav. Sci..

[143] Thomas Yeo B.T., Krienen F.M., Sepulcre J., Sabuncu M.R., Lashkari D., Hollinshead M., Roffman J.L., Smoller J.W., Zöllei L., Polimeni J.R. (2011). The organization of the human cerebral cortex estimated by intrinsic functional connectivity. J. Neurophysiol..

[144] Griffiths K.R., Braund T.A., Kohn M.R., Clarke S., Williams L.M., Korgaonkar M.S. (2021). Structural brain network topology underpinning ADHD and response to methylphenidate treatment. Transl. Psychiatry.

[145] Puchkov N.A., Tarumov D.A., Markin K.V., Prochik Y.E., Tyomniy A.V., Maslov V.E. (2021). Pathology of microstructural brain connectivity in paranoid schizophrenia (according to diffusion-tensor tractography data). Russ. Mil. Med. Acad. Rep..

[146] Rubinov M., Bullmore E. (2022). Schizophrenia and abnormal brain network hubs. Dialogues Clin. Neurosci..

[147] Li C., Li Y., Zheng L., Zhu X., Shao B., Fan G., Liu T., Wang J. (2019). Abnormal Brain Network Connectivity in a Triple-Network Model of Alzheimer’s Disease. J. Alzheimer’s Dis..

[148] Dickerson B.C., Sperling R.A. (2009). Large-scale functional brain network abnormalities in Alzheimer’s disease: Insights from functional neuroimaging. Behav. Neurol..

[149] Binnewijzend M.A.A., Schoonheim M.M., Sanz-Arigita E., Wink A.M., van der Flier W.M., Tolboom N., Adriaanse S.M., Damoiseaux J.S., Scheltens P., van Berckel B.N.M. (2012). Resting-state fMRI changes in Alzheimer’s disease and mild cognitive impairment. Neurobiol. Aging.

[150] Greicius M.D., Srivastava G., Reiss A.L., Menon V. (2004). Default-mode network activity distinguishes Alzheimer’s disease from healthy aging: Evidence from functional MRI. Proc. Natl. Acad. Sci. USA.

[151] He X., Qin W., Liu Y., Zhang X., Duan Y., Song J., Li K., Jiang T., Yu C. (2014). Abnormal salience network in normal aging and in amnestic mild cognitive impairment and Alzheimer’s disease. Hum. Brain Mapp..

[152] Teipel S.J. (2010). S5-02-06: DTI and resting state fMRI as biomarker of Alzheimer’s disease: Present state and perspectives. Alzheimer’s Dement..

[153] Wang K., Liang M., Wang L., Tian L., Zhang X., Li K., Jiang T. (2006). Altered functional connectivity in early Alzheimer’s disease: A resting-state fMRI study. Hum. Brain Mapp..

[154] Wang P., Zhou B., Yao H., Zhan Y., Zhang Z., Cui Y., Xu K., Ma J., Wang L., An N. (2015). Aberrant intra- and inter-network connectivity architectures in Alzheimer’s disease and mild cognitive impairment. Sci. Rep..

[155] Zhong Y., Huang L., Cai S., Zhang Y., von Deneen K.M., Ren A., Ren J. (2014). Altered effective connectivity patterns of the default mode network in Alzheimer’s disease: An fMRI study. Neurosci. Lett..

[156] Gavron A.A., Deza-Araujo Y.I., Sharova E.V., Smirnov A.S., Knyazev G.G., Chelyapina M.V., Fadeeva L.M., Abdulaev A.A., Kulikov M.A., Zhavoronkova L.A. (2020). Group and Individual fMRI Analysis of the Main Resting State Networks in Healthy Subjects. Neurosci. Behav. Physiol..

[157] Lim H.K., Nebes R., Snitz B., Cohen A., Mathis C., Price J., Weissfeld L., Klunk W., Aizenstein H.J. (2014). Regional amyloid burden and intrinsic connectivity networks in cognitively normal elderly subjects. Brain.

[158] Zhou J., Greicius M.D., Gennatas E.D., Growdon M.E., Jang J.Y., Rabinovici G.D., Kramer J.H., Weiner M., Miller B.L., Seeley W.W. (2010). Divergent network connectivity changes in behavioural variant frontotemporal dementia and Alzheimer’s disease. Brain.

[159] Onoda K., Ishihara M., Yamaguchi S. (2012). Decreased Functional Connectivity by Aging Is Associated with Cognitive Decline. J. Cogn. Neurosci..

[160] Li R., Wu X., Chen K., Fleisher A.S., Reiman E.M., Yao L. (2013). Alterations of Directional Connectivity among Resting-State Networks in Alzheimer Disease. Am. J. Neuroradiol..

[161] Liu X., Chen X., Zheng W., Xia M., Han Y., Song H., Li K., He Y., Wang Z. (2018). Altered Functional Connectivity of Insular Subregions in Alzheimer’s Disease. Front. Aging Neurosci..

[162] Tanashyan M.M., Lagoda O.V., Medvedev R.B., Krotenkova M.V., Konovalov R.N., Ponomareva N.V., Fokin V.F. (2020). Resting-state neural networks in cognitive decline in patients with vascular encephalopathy. Ann. Clin. Exp. Neurol..

[163] Galanin I.V., Naryshkin A.G., Liaskina I.Y., Skoromets T.A. (2022). Morphometric and Functional Changes of the Brain in Mental Disorders and Their Dynamics during Drug Treatment. Hum. Physiol..

[164] Whitfield-Gabrieli S., Nieto-Castanon A. (2012). Conn: A Functional Connectivity Toolbox for Correlated and Anticorrelated Brain Networks. Brain Connect..

[165] Lee T.W., Northoff G., Wu Y.T. (2014). Resting network is composed of more than one neural pattern: An fMRI study. Neuroscience.

[166] Mulders P.C., van Eijndhoven P.F., Schene A.H., Beckmann C.F., Tendolkar I. (2015). Resting-state functional connectivity in major depressive disorder: A review. Neurosci. Biobehav. Rev..

[167] Stoyanov D., Khorev V., Paunova R., Kandilarova S., Simeonova D., Badarin A., Hramov A., Kurkin S. (2022). Resting-State Functional Connectivity Impairment in Patients with Major Depressive Episode. Int. J. Environ. Res. Public Health.

[168] Raichle M.E. (2015). The Brain’s Default Mode Network. Annu. Rev. Neurosci..

[169] Lee T.W., Xue S.W. (2018). Functional connectivity maps based on hippocampal and thalamic dynamics may account for the default-mode network. Eur. J. Neurosci..

[170] Gusnard D.A., Akbudak E., Shulman G.L., Raichle M.E. (2001). Medial prefrontal cortex and self-referential mental activity: Relation to a default mode of brain function. Proc. Natl. Acad. Sci. USA.

[171] Hayasaka S., Konishi M., McLaren D.G., Engen H., Smallwood J. (2015). Shaped by the Past: The Default Mode Network Supports Cognition that Is Independent of Immediate Perceptual Input. PLoS ONE.

[172] Østby Y., Walhovd K.B., Tamnes C.K., Grydeland H., Westlye L.T., Fjell A.M. (2012). Mental time travel and default-mode network functional connectivity in the developing brain. Proc. Natl. Acad. Sci. USA.

[173] Sali A.W., Courtney S.M., Yantis S. (2016). Spontaneous Fluctuations in the Flexible Control of Covert Attention. J. Neurosci..

[174] Vatansever D., Manktelow A.E., Sahakian B.J., Menon D.K., Stamatakis E.A. (2016). Cognitive Flexibility: A Default Network and Basal Ganglia Connectivity Perspective. Brain Connect..

[175] Davey C.G., Pujol J., Harrison B.J. (2016). Mapping the self in the brain’s default mode network. NeuroImage.

[176] Qin P., Grimm S., Duncan N.W., Fan Y., Huang Z., Lane T., Weng X., Bajbouj M., Northoff G. (2016). Spontaneous activity in default-mode network predicts ascription of self-relatedness to stimuli. Soc. Cogn. Affect. Neurosci..

[177] Li W., Mai X., Liu C. (2014). The default mode network and social understanding of others: What do brain connectivity studies tell us. Front. Hum. Neurosci..

[178] Mars R.B., Neubert F.-X., Noonan M.P., Sallet J., Toni I., Rushworth M.F.S. (2012). On the relationship between the “default mode network” and the “social brain”. Front. Hum. Neurosci..

[179] Andreasen N. (2011). A journey into chaos: Creativity and the unconsciousFNx08. Mens Sana Monogr..

[180] Beaty R.E., Benedek M., Wilkins R.W., Jauk E., Fink A., Silvia P.J., Hodges D.A., Koschutnig K., Neubauer A.C. (2014). Creativity and the default network: A functional connectivity analysis of the creative brain at rest. Neuropsychologia.

[181] Knyazev G.G., Ushakov V.L., Orlov V.A., Malakhov D.G., Kartashov S.I., Savostyanov A.N., Bocharov A.V., Velichkovsky B.M. (2021). EEG and fMRI Correlates of Insight: A Pilot Study. Symmetry.

[182] Ino T., Nakai R., Azuma T., Kimura T., Fukuyama H. (2011). Brain Activation During Autobiographical Memory Retrieval with Special Reference to Default Mode Network. Open Neuroimaging J..

[183] Yang J., Weng X., Zang Y., Xu M., Xu X. (2010). Sustained activity within the default mode network during an implicit memory task. Cortex.

[184] Whitfield-Gabrieli S., Ford J.M. (2012). Default Mode Network Activity and Connectivity in Psychopathology. Annu. Rev. Clin. Psychol..

[185] Northoff G., Bermpohl F. (2004). Cortical midline structures and the self. Trends Cogn. Sci..

[186] Hou X., Liu R., Zhou Y., Guan L., Zhou J., Liu J., Liu M., Yuan X., Feng Y., Chen X. (2024). Shared and unique alterations of large-scale network connectivity in drug-free adolescent-onset and adult-onset major depressive disorder. Transl. Psychiatry.

[187] Kaiser R.H., Andrews-Hanna J.R., Wager T.D., Pizzagalli D.A. (2015). Large-Scale Network Dysfunction in Major Depressive Disorder. JAMA Psychiatry.

[188] Sun H., Cui H., Sun Q., Li Y., Bai T., Wang K., Zhang J., Tian Y., Wang J. (2024). Individual large-scale functional network mapping for major depressive disorder with electroconvulsive therapy. J. Affect. Disord..

[189] Dong D., Wang Y., Chang X., Luo C., Yao D. (2018). Dysfunction of Large-Scale Brain Networks in Schizophrenia: A Meta-analysis of Resting-State Functional Connectivity. Schizophr. Bull..

[190] Kraguljac N.V., White D.M., Hadley J.A., Visscher K., Knight D., ver Hoef L., Falola B., Lahti A.C. (2016). Abnormalities in large scale functional networks in unmedicated patients with schizophrenia and effects of risperidone. NeuroImage Clin..

[191] He Q., Colon-Motas K.M., Pybus A.F., Piendel L., Seppa J.K., Walker M.L., Manzanares C.M., Qiu D., Miocinovic S., Wood L.B. (2021). A feasibility trial of gamma sensory flicker for patients with prodromal Alzheimer’s disease. Alzheimer’s Dement. Transl. Res. Clin. Interv..

[192] Chan D., Suk H.-J., Jackson B., Milman N.P., Stark D., Klerman E.B., Kitchener E., Avalos V.S.F., Banerjee A., Beach S.D. (2021). 40Hz sensory stimulation induces gamma entrainment and affects brain structure, sleep and cognition in patients with Alzheimer’s dementia. medRxiv.

[193] Gusnard D.A., Raichle M.E. (2001). Searching for a baseline: Functional imaging and the resting human brain. Nat. Rev. Neurosci..

[194] Raichle M.E., MacLeod A.M., Snyder A.Z., Powers W.J., Gusnard D.A., Shulman G.L. (2001). A default mode of brain function. Proc. Natl. Acad. Sci. USA.

[195] Scolari M., Seidl-Rathkopf K.N., Kastner S. (2015). Functions of the human frontoparietal attention network: Evidence from neuroimaging. Curr. Opin. Behav. Sci..

[196] Uddin L.Q., Yeo B.T.T., Spreng R.N. (2019). Towards a Universal Taxonomy of Macro-scale Functional Human Brain Networks. Brain Topogr..

[197] Kornblith S., Buschman T.J., Miller E.K. (2016). Stimulus Load and Oscillatory Activity in Higher Cortex. Cereb. Cortex.

[198] Palva S., Kulashekhar S., Hämäläinen M., Palva J.M. (2011). Localization of Cortical Phase and Amplitude Dynamics during Visual Working Memory Encoding and Retention. J. Neurosci..

[199] Tseng P., Chang Y.-T., Chang C.-F., Liang W.-K., Juan C.-H. (2016). The critical role of phase difference in gamma oscillation within the temporoparietal network for binding visual working memory. Sci. Rep..

[200] Schultz W. (2015). Brain Mapping.

[201] Steimke R., Nomi J.S., Calhoun V.D., Stelzel C., Paschke L.M., Gaschler R., Goschke T., Walter H., Uddin L.Q. (2017). Salience network dynamics underlying successful resistance of temptation. Soc. Cogn. Affect. Neurosci..

[202] Bertolero M., Bassett D.S., Studios M.R. (2024). How Matter Becomes Mind. Sci. Am..

[203] Buckner R.L. (2012). The serendipitous discovery of the brain’s default network. NeuroImage.

[204] Riedl V., Utz L., Castrillón G., Grimmer T., Rauschecker J.P., Ploner M., Friston K.J., Drzezga A., Sorg C. (2015). Metabolic connectivity mapping reveals effective connectivity in the resting human brain. Proc. Natl. Acad. Sci. USA.

[205] Bailey S.K., Aboud K.S., Nguyen T.Q., Cutting L.E. (2018). Applying a network framework to the neurobiology of reading and dyslexia. J. Neurodev. Disord..

[206] Yan H., Deng X., Wang Y., Wu S., Du J., Yu M., Liu B., Wang H., Pan Y., Zhang Z. (2024). Enhancement of spatial learning by 40 Hz visual stimulation requires parvalbumin interneuron-dependent hippocampal neurogenesis. bioRxiv.

[207] Brown J., Cooper-Kuhn C.M., Kempermann G., Van Praag H., Winkler J., Gage F.H., Kuhn H.G. (2003). Enriched environment and physical activity stimulate hippocampal but not olfactory bulb neurogenesis. Eur. J. Neurosci..

[208] Nilsson M., Perfilieva E., Johansson U., Orwar O., Eriksson P.S. (1999). Enriched environment increases neurogenesis in the adult rat dentate gyrus and improves spatial memory. J. Neurobiol..

[209] van Praag H., Christie B.R., Sejnowski T.J., Gage F.H. (1999). Running enhances neurogenesis, learning, and long-term potentiation in mice. Proc. Natl. Acad. Sci. USA.

[210] Kempermann G., Kuhn H.G., Gage F.H. (1998). Experience-Induced Neurogenesis in the Senescent Dentate Gyrus. J. Neurosci..

[211] Song J., Olsen R.H.J., Sun J., Ming G.-l., Song H. (2016). Neuronal Circuitry Mechanisms Regulating Adult Mammalian Neurogenesis. Cold Spring Harb. Perspect. Biol..

[212] Hu H., Gan J., Jonas P. (2014). Fast-spiking, parvalbumin+ GABAergic interneurons: From cellular design to microcircuit function. Science.

[213] Lodge M., Bischofberger J. (2019). Synaptic properties of newly generated granule cells support sparse coding in the adult hippocampus. Behav. Brain Res..

[214] Song J., Sun J., Moss J., Wen Z., Sun G.J., Hsu D., Zhong C., Davoudi H., Christian K.M., Toni N. (2013). Parvalbumin interneurons mediate neuronal circuitry–neurogenesis coupling in the adult hippocampus. Nat. Neurosci..

[215] Catavero C., Bao H., Song J. (2017). Neural mechanisms underlying GABAergic regulation of adult hippocampal neurogenesis. Cell Tissue Res..

[216] Markwardt S.J., Wadiche J.I., Overstreet-Wadiche L.S. (2009). Input-Specific GABAergic Signaling to Newborn Neurons in Adult Dentate Gyrus. J. Neurosci..

[217] Sibbe M., Kulik A. (2016). GABAergic Regulation of Adult Hippocampal Neurogenesis. Mol. Neurobiol..

[218] Tozuka Y., Fukuda S., Namba T., Seki T., Hisatsune T. (2005). GABAergic Excitation Promotes Neuronal Differentiation in Adult Hippocampal Progenitor Cells. Neuron.

[219] Pallotto M., Deprez F. (2014). Regulation of adult neurogenesis by GABAergic transmission: Signaling beyond GABAA-receptors. Front. Cell. Neurosci..

[220] Pontes A., Zhang Y., Hu W. (2013). Novel functions of GABA signaling in adult neurogenesis. Front. Biol..

[221] Lord L.-D., Expert P., Huckins J.F., Turkheimer F.E. (2013). Cerebral Energy Metabolism and the Brain’s Functional Network Architecture: An Integrative Review. J. Cereb. Blood Flow Metab..

[222] Freeman R.D., Li B. (2016). Neural–metabolic coupling in the central visual pathway. Philos. Trans. R. Soc. B Biol. Sci..

[223] Niessing J.r., Ebisch B., Schmidt K.E., Niessing M., Singer W., Galuske R.A.W. (2005). Hemodynamic Signals Correlate Tightly with Synchronized Gamma Oscillations. Science.

[224] Borea P.A., Gessi S., Merighi S., Vincenzi F., Varani K. (2018). Pharmacology of Adenosine Receptors: The State of the Art. Physiol. Rev..

[225] Chu S., Xiong W., Zhang D., Soylu H., Sun C., Albensi B.C., Parkinson F.E. (2012). Regulation of adenosine levels during cerebral ischemia. Acta Pharmacol. Sin..

[226] Garcia-Gil M., Camici M., Allegrini S., Pesi R., Tozzi M.G. (2021). Metabolic Aspects of Adenosine Functions in the Brain. Front. Pharmacol..

[227] Chen J.-F., Eltzschig H.K., Fredholm B.B. (2013). Adenosine receptors as drug targets—What are the challenges?. Nat. Rev. Drug Discov..

[228] Cunha R.A. (2016). How does adenosine control neuronal dysfunction and neurodegeneration?. J. Neurochem..

[229] Shlyakhto E.V., Barantsevich E.P., Shcherbak N.S., Galagudza M.M. (2012). Molecular Mechanisms of Development of Cerebral Tolerance to Ischemia. Part 1. Ann. Russ. Acad. Med. Sci..

[230] Pomytkin I.A., Karkischenko N.N. (2019). Metabolic Control of High-Frequency Gamma Oscillations in the Brain. Biomeditsina.

[231] Fielder E., Tweedy C., Wilson C., Oakley F., LeBeau F.E.N., Passos J.F., Mann D.A., von Zglinicki T., Jurk D. (2020). Anti-inflammatory treatment rescues memory deficits during aging in nfkb1−/− mice. Aging Cell.

[232] Schilling S., Chausse B., Dikmen H.O., Almouhanna F., Hollnagel J.O., Lewen A., Kann O. (2021). TLR2- and TLR3-activated microglia induce different levels of neuronal network dysfunction in a context-dependent manner. Brain Behav. Immun..

[233] Ta T.-T., Dikmen H.O., Schilling S., Chausse B., Lewen A., Hollnagel J.-O., Kann O. (2019). Priming of microglia with IFN-γ slows neuronal gamma oscillations in situ. Proc. Natl. Acad. Sci. USA.

[234] Assogna M., Casula E.P., Borghi I., Bonnì S., Samà D., Motta C., Di Lorenzo F., D’Acunto A., Porrazzini F., Minei M. (2020). Effects of Palmitoylethanolamide Combined with Luteoline on Frontal Lobe Functions, High Frequency Oscillations, and GABAergic Transmission in Patients with Frontotemporal Dementia. J. Alzheimer’s Dis..

[235] Chausse B., Lewen A., Poschet G., Kann O. (2020). Selective inhibition of mitochondrial respiratory complexes controls the transition of microglia into a neurotoxic phenotype in situ. Brain Behav. Immun..

[236] Salter M.W., Stevens B. (2017). Microglia emerge as central players in brain disease. Nat. Med..

[237] Ferro A., Auguste Y.S.S., Cheadle L. (2021). Microglia, Cytokines, and Neural Activity: Unexpected Interactions in Brain Development and Function. Front. Immunol..

[238] Tremblay M.-È., Stevens B., Sierra A., Wake H., Bessis A., Nimmerjahn A. (2011). The Role of Microglia in the Healthy Brain: Figure 1. J. Neurosci..

[239] Lobo-Silva D., Carriche G.M., Castro A.G., Roque S., Saraiva M. (2016). Balancing the immune response in the brain: IL-10 and its regulation. J. Neuroinflamm..

[240] Martinez F.O., Helming L., Gordon S. (2009). Alternative Activation of Macrophages: An Immunologic Functional Perspective. Annu. Rev. Immunol..

[241] Fernandes A., Miller-Fleming L., Pais T.F. (2014). Microglia and inflammation: Conspiracy, controversy or control?. Cell. Mol. Life Sci..

[242] Nakagawa Y., Chiba K. (2015). Diversity and plasticity of microglial cells in psychiatric and neurological disorders. Pharmacol. Ther..

[243] Tang Y., Le W. (2015). Differential Roles of M1 and M2 Microglia in Neurodegenerative Diseases. Mol. Neurobiol..

[244] Lyamtsev A.S., Sentyabreva A.V., Kosyreva A.M. (2025). The Role of Prenatal Microglial Activation and Its Sex Differences in the Development of Neuropsychiatric Disorders and Neurodegenerative Diseases. Int. J. Mol. Sci..

[245] Miron V.E., Boyd A., Zhao J.-W., Yuen T.J., Ruckh J.M., Shadrach J.L., van Wijngaarden P., Wagers A.J., Williams A., Franklin R.J.M. (2013). M2 microglia and macrophages drive oligodendrocyte differentiation during CNS remyelination. Nat. Neurosci..

[246] Hines D.J., Hines R.M., Mulligan S.J., Macvicar B.A. (2009). Microglia processes block the spread of damage in the brain and require functional chloride channels. Glia.

[247] Spittau B. (2017). Aging Microglia—Phenotypes, Functions and Implications for Age-Related Neurodegenerative Diseases. Front. Aging Neurosci..

[248] Hanisch U.K. (2002). Microglia as a source and target of cytokines. Glia.

[249] Lyons Y.A., Pradeep S., Wu S.Y., Haemmerle M., Hansen J.M., Wagner M.J., Villar-Prados A., Nagaraja A.S., Dood R.L., Previs R.A. (2017). Macrophage depletion through colony stimulating factor 1 receptor pathway blockade overcomes adaptive resistance to anti-VEGF therapy. Oncotarget.

[250] Anderson W.D., Greenhalgh A.D., Takwale A., David S., Vadigepalli R. (2017). Novel Influences of IL-10 on CNS Inflammation Revealed by Integrated Analyses of Cytokine Networks and Microglial Morphology. Front. Cell. Neurosci..

[251] Balasingam V., Yong V.W. (1996). Attenuation of Astroglial Reactivity by Interleukin-10. J. Neurosci..

[252] Ledeboer A., Brevé J.J.P., Wierinckx A., Van Der Jagt S., Bristow A.F., Leysen J.E., Tilders F.J.H., Van Dam A.M. (2002). Expression and regulation of interleukin-10 and interleukin-10 receptor in rat astroglial and microglial cells. Eur. J. Neurosci..

[253] Zhou Z., Peng X., Insolera R., Fink D.J., Mata M. (2009). IL-10 promotes neuronal survival following spinal cord injury. Exp. Neurol..

[254] Pereira L., Font-Nieves M., Van den Haute C., Baekelandt V., Planas A.M., Pozas E. (2015). IL-10 regulates adult neurogenesis by modulating ERK and STAT3 activity. Front. Cell. Neurosci..

[255] Perez-Asensio F.J., Perpiñá U., Planas A.M., Pozas E. (2013). Interleukin-10 regulates progenitor differentiation and modulates neurogenesis on adult brain. J. Cell Sci..

[256] Zhou Z., Peng X., Insolera R., Fink D.J., Mata M. (2009). Interleukin-10 provides direct trophic support to neurons. J. Neurochem..

[257] Synchikova A.P., Korneva E.A. (2022). Morphological and functional characteristics of LPS-stimulated microglial cells under the action of orexin A. Russ. J. Immunol..

[258] Kiyota T., Ingraham K.L., Swan R.J., Jacobsen M.T., Andrews S.J., Ikezu T. (2011). AAV serotype 2/1-mediated gene delivery of anti-inflammatory interleukin-10 enhances neurogenesis and cognitive function in APP+PS1 mice. Gene Ther..

[259] Van Boxel-Dezaire A.H.H., Hoff S.C.J., Van Oosten B.W., Verweij C.L., Dräger A.M., Adèr H.J., Van Houwelingen J.C., Barkhof F., Polman C.H., Nagelkerken L. (1999). Decreased interleukin-10 and increased interleukin-12p40 mRNA are associated with disease activity and characterize different disease stages in multiple sclerosis. Ann. Neurol..

[260] Hesse D., Krakauer M., Lund H., Søndergaard H.B., Limborg S.J.W., Soelberg Sørensen P., Sellebjerg F. (2011). Disease protection and interleukin-10 induction by endogenous interferon-β in multiple sclerosis?. Eur. J. Neurol..

[261] Arimoto T., Choi D.-Y., Lu X., Liu M., Nguyen X.V., Zheng N., Stewart C.A., Kim H.-C., Bing G. (2007). Interleukin-10 protects against inflammation-mediated degeneration of dopaminergic neurons in substantia nigra. Neurobiol. Aging.

[262] George A., Kleinschnitz C., Zelenka M., Brinkhoff J., Stoll G., Sommer C. (2004). Wallerian degeneration after crush or chronic constriction injury of rodent sciatic nerve is associated with a depletion of endoneurial interleukin-10 protein. Exp. Neurol..

[263] Garza K.M., Zhang L., Borron B., Wood L.B., Singer A.C. (2020). Gamma Visual Stimulation Induces a Neuroimmune Signaling Profile Distinct from Acute Neuroinflammation. J. Neurosci..

[264] Chistyakov D.V., Astakhova A.A., Azbukina N.V., Goriainov S.V., Chistyakov V.V., Sergeeva M.G. (2019). Cellular Model of Endotoxin Tolerance in Astrocytes: Role of Interleukin 10 and Oxylipins. Cells.

[265] Chistyakov D.V., Azbukina N.V., Astakhova A.A., Goriainov S.V., Chistyakov V.V., Sergeeva M.G. (2018). Sex-Mediated Differences in LPS Induced Alterations of TNFα, IL-10 Expression, and Prostaglandin Synthesis in Primary Astrocytes. Int. J. Mol. Sci..

[266] Park C., Lee S., Cho I.H., Lee H.K., Kim D., Choi S.Y., Oh S.B., Park K., Kim J.S., Lee S.J. (2005). TLR3-mediated signal induces proinflammatory cytokine and chemokine gene expression in astrocytes: Differential signaling mechanisms of TLR3-induced IP-10 and IL-8 gene expression. Glia.

[267] Chakrabarty P., Li A., Ceballos-Diaz C., Eddy J.A., Funk C.C., Moore B., DiNunno N., Rosario A.M., Cruz P.E., Verbeeck C. (2015). IL-10 Alters Immunoproteostasis in APP Mice, Increasing Plaque Burden and Worsening Cognitive Behavior. Neuron.

[268] Guillot-Sestier M.-V., Doty K.R., Gate D., Rodriguez J., Leung B.P., Rezai-Zadeh K., Town T. (2015). Il10 Deficiency Rebalances Innate Immunity to Mitigate Alzheimer-Like Pathology. Neuron.

[269] Kettenmann H., Hanisch U.-K., Noda M., Verkhratsky A. (2011). Physiology of Microglia. Physiol. Rev..

[270] Ismail R., Hansen A.K., Parbo P., Brændgaard H., Gottrup H., Brooks D.J., Borghammer P. (2018). The Effect of 40-Hz Light Therapy on Amyloid Load in Patients with Prodromal and Clinical Alzheimer’s Disease. Int. J. Alzheimer’s Dis..

[271] Notbohm A., Herrmann C.S. (2016). Flicker Regularity Is Crucial for Entrainment of Alpha Oscillations. Front. Hum. Neurosci..

[272] Notbohm A., Kurths J., Herrmann C.S. (2016). Modification of Brain Oscillations via Rhythmic Light Stimulation Provides Evidence for Entrainment but Not for Superposition of Event-Related Responses. Front. Hum. Neurosci..

[273] Chan A.S., Lee T.-l., Hamblin M.R., Cheung M.-c. (2021). Photobiomodulation Enhances Memory Processing in Older Adults with Mild Cognitive Impairment: A Functional Near-Infrared Spectroscopy Study. J. Alzheimer’s Dis..

[274] Yao D., Horwitz A., Dyhr Thomsen M., Wiegand I., Horwitz H., Klemp M., Nikolic M., Rask L., Lauritzen M., Benedek K. (2017). Visual steady state in relation to age and cognitive function. PLoS ONE.

[275] Schneck M.E., Haegerstrom-Portnoy G., Lott L.A., Brabyn J.A. (2014). Comparison of Panel D-15 Tests in a Large Older Population. Optom. Vis. Sci..

[276] Nguyen-Tri D., Overbury O., Faubert J. (2003). The Role of Lenticular Senescence in Age-Related Color Vision Changes. Investig. Opthalmol. Vis. Sci..

[277] Tsoneva T., Garcia-Molina G., Desain P. (2015). Neural dynamics during repetitive visual stimulation. J. Neural Eng..

[278] Zhang J., Yue X., Luo H., Jiang W., Mei Y., Ai L., Gao G., Wu Y., Yang H., An J. (2019). Illumination with 630 nm Red Light Reduces Oxidative Stress and Restores Memory by Photo-Activating Catalase and Formaldehyde Dehydrogenase in SAMP8 Mice. Antioxid. Redox Signal..

[279] Megerian J., Hajos M., Hempel E., Cimenser A., Boasso A., Cotter C., Williams M., Kwan K., Nicodemus-Johnson J., Hendrix S. (2022). Feasibility, Safety, and Efficacy of Gamma Sensory Stimulation as a Novel Therapeutic Intervention for Alzheimer’s Disease (N1.001). Neurology.

[280] Shady S., MacLeod D.I.A., Fisher H.S. (2004). Adaptation from invisible flicker. Proc. Natl. Acad. Sci. USA.

[281] Henney M.A., Carstensen M., Thorning-Schmidt M., Kubińska M., Grønberg M.G., Nguyen M., Madsen K.H., Clemmensen L.K.H., Petersen P.M. (2024). Brain stimulation with 40 Hz heterochromatic flicker extended beyond red, green, and blue. Sci. Rep..

[282] Agger M.P., Carstensen M.S., Henney M.A., Hansen L.S., Baandrup A.O., Nguyen M., Petersen P.M., Madsen K.H., Kjær T.W. (2022). Novel Invisible Spectral Flicker Induces 40 Hz Neural Entrainment with Similar Spatial Distribution as 40 Hz Stroboscopic Light. J. Alzheimer’s Dis..

[283] Terman M., Terman J.S. (2014). Light Therapy for Seasonal and Nonseasonal Depression: Efficacy, Protocol, Safety, and Side Effects. CNS Spectr..

[284] Liu C.-R., Liou Y.M., Jou J.-H. (2021). Pilot Study of the Effects of Bright Ambient Therapy on Dementia Symptoms and Cognitive Function. Front. Psychol..

[285] Hainke L., Dowsett J., Spitschan M., Priller J. (2025). 40 Hz visual stimulation during sleep evokes neuronal gamma activity in NREM and REM stages. Sleep.

[286] Cordone S., Annarumma L., Rossini P.M., De Gennaro L. (2019). Sleep and β-Amyloid Deposition in Alzheimer Disease: Insights on Mechanisms and Possible Innovative Treatments. Front. Pharmacol..

[287] Xie L., Kang H., Xu Q., Chen M.J., Liao Y., Thiyagarajan M., O’Donnell J., Christensen D.J., Nicholson C., Iliff J.J. (2013). Sleep Drives Metabolite Clearance from the Adult Brain. Science.

[288] Semyachkina-Glushkovskaya O., Fedosov I., Penzel T., Li D., Yu T., Telnova V., Kaybeleva E., Saranceva E., Terskov A., Khorovodov A. (2023). Brain Waste Removal System and Sleep: Photobiomodulation as an Innovative Strategy for Night Therapy of Brain Diseases. Int. J. Mol. Sci..

[289] Christensen J., Yamakawa G.R., Shultz S.R., Mychasiuk R. (2021). Is the glymphatic system the missing link between sleep impairments and neurological disorders? Examining the implications and uncertainties. Prog. Neurobiol..

[290] Terskov A., Evsukova A., Blokhina I., Tzoy M., Zlatogorskaya D., Adushkina V. (2024). Photo-sleep therapy of Alzheimer’s disease. Eur. Phys. J. Spec. Top..

[291] Semyachkina-Glushkovskaya O., Penzel T., Poluektov M., Fedosov I., Tzoy M., Terskov A., Blokhina I., Sidorov V., Kurths J. (2023). Phototherapy of Alzheimer’s Disease: Photostimulation of Brain Lymphatics during Sleep: A Systematic Review. Int. J. Mol. Sci..

[292] Lim L. (2024). Modifying Alzheimer’s disease pathophysiology with photobiomodulation: Model, evidence, and future with EEG-guided intervention. Front. Neurol..

[293] Zhao X., Du W., Jiang J., Han Y., Zhu L.-Q. (2022). Brain Photobiomodulation Improves Sleep Quality in Subjective Cognitive Decline: A Randomized, Sham-Controlled Study. J. Alzheimer’s Dis..

[294] Mitrofanis J., Valverde A., Hamilton C., Moro C., Billeres M., Magistretti P. (2023). Lights at night: Does photobiomodulation improve sleep?. Neural Regen. Res..

[295] van Hattem T., Verkaar L., Krugliakova E., Adelhöfer N., Zeising M., Drinkenburg W.H.I.M., Claassen J.A.H.R., Bódizs R., Dresler M., Rosenblum Y. (2025). Targeting Sleep Physiology to Modulate Glymphatic Brain Clearance. Physiology.

[296] Murphy M., Öngür D. (2019). Decreased peak alpha frequency and impaired visual evoked potentials in first episode psychosis. NeuroImage Clin..

[297] Khuwaja G.A., Haghighi S.J., Hatzinakos D. (2015). 40-Hz ASSR fusion classification system for observing sleep patterns. EURASIP J. Bioinform. Syst. Biol..

[298] Lustenberger C., Patel Y.A., Alagapan S., Page J.M., Price B., Boyle M.R., Fröhlich F. (2018). High-density EEG characterization of brain responses to auditory rhythmic stimuli during wakefulness and NREM sleep. NeuroImage.

[299] Zhou X., He Y., Xu T., Wu Z., Guo W., Xu X., Liu Y., Zhang Y., Shang H., Huang L. (2024). 40 Hz light flickering promotes sleep through cortical adenosine signaling. Cell Res..

[300] Liu Y., Li X., Liu S., Liang T., Wu Y., Wang X., Li Y., Xu Y. (2024). Study on Gamma sensory flicker for Insomnia. Int. J. Neurosci..

[301] Gross D.W., Gotman J. (1999). Correlation of high-frequency oscillations with the sleep–wake cycle and cognitive activity in humans. Neuroscience.

[302] Park S.-S., Park H.-S., Kim C.-J., Baek S.-S., Park S.-Y., Anderson C., Kim M.-K., Park I.-R., Kim T.-W. (2022). Combined effects of aerobic exercise and 40-Hz light flicker exposure on early cognitive impairments in Alzheimer’s disease of 3×Tg mice. J. Appl. Physiol..

[303] Barry R.J., Clarke A.R., Johnstone S.J., Magee C.A., Rushby J.A. (2007). EEG differences between eyes-closed and eyes-open resting conditions. Clin. Neurophysiol..

[304] Han C., Zhao X., Li M., Haihambo N., Teng J., Li S., Qiu J., Feng X., Gao M. (2022). Enhancement of the neural response during 40 Hz auditory entrainment in closed-eye state in human prefrontal region. Cogn. Neurodyn..

[305] Perry G., Cash A.D., Smith M.A. (2002). Alzheimer Disease and Oxidative Stress. BioMed Res. Int..

[306] Schubert D. (2005). Glucose metabolism and Alzheimer’s disease. Ageing Res. Rev..

[307] Hajós M., Boasso A., Hempel E., Shpokayte M., Konisky A., Seshagiri C.V., Fomenko V., Kwan K., Nicodemus-Johnson J., Hendrix S. (2024). Safety, tolerability, and efficacy estimate of evoked gamma oscillation in mild to moderate Alzheimer’s disease. Front. Neurol..

[308] Deuschl G., Schade-Brittinger C., Krack P., Volkmann J., Schäfer H., Bötzel K., Daniels C., Deutschländer A., Dillmann U., Eisner W. (2006). A Randomized Trial of Deep-Brain Stimulation for Parkinson’s Disease. N. Engl. J. Med..

[309] Muthuraman M., Bange M., Koirala N., Ciolac D., Pintea B., Glaser M., Tinkhauser G., Brown P., Deuschl G., Groppa S. (2020). Cross-frequency coupling between gamma oscillations and deep brain stimulation frequency in Parkinson’s disease. Brain.

[310] Okun M.S., Fernandez H.H., Wu S.S., Kirsch-Darrow L., Bowers D., Bova F., Suelter M., Jacobson C.E., Wang X., Gordon C.W. (2009). Cognition and mood in Parkinson’s disease in subthalamic nucleus versus globus pallidus interna deep brain stimulation: The COMPARE Trial. Ann. Neurol..

[311] Baptista M.A.T., Kimura N., Lacerda I.B., Silva F.d.O., Dourado M.C.N., Caramelli P. (2021). Domains of Awareness in Young and Late Onset Dementia. J. Alzheimer’s Dis..

[312] Saltmarche A.E., Naeser M.A., Ho K.F., Hamblin M.R., Lim L. (2017). Significant Improvement in Cognition in Mild to Moderately Severe Dementia Cases Treated with Transcranial Plus Intranasal Photobiomodulation: Case Series Report. Photomed. Laser Surg..

[313] Chao L.L. (2019). Effects of Home Photobiomodulation Treatments on Cognitive and Behavioral Function, Cerebral Perfusion, and Resting-State Functional Connectivity in Patients with Dementia: A Pilot Trial. Photobiomodul. Photomed. Laser Surg..

[314] Ewbank M.P., Andrews T.J. (2008). Differential sensitivity for viewpoint between familiar and unfamiliar faces in human visual cortex. NeuroImage.

[315] Guerra A., Colella D., Giangrosso M., Cannavacciuolo A., Paparella G., Fabbrini G., Suppa A., Berardelli A., Bologna M. (2022). Driving motor cortex oscillations modulates bradykinesia in Parkinson’s disease. Brain.

[316] Senefeld J.W., Hunter S.K., Coleman D., Joyner M.J. (2023). Case Studies in Physiology: Male to female transgender swimmer in college athletics. J. Appl. Physiol..

